# Genomic encyclopedia of sugar utilization pathways in the *Shewanella *genus

**DOI:** 10.1186/1471-2164-11-494

**Published:** 2010-09-13

**Authors:** Dmitry A Rodionov, Chen Yang, Xiaoqing Li, Irina A Rodionova, Yanbing Wang, Anna Y Obraztsova, Olga P Zagnitko, Ross Overbeek, Margaret F Romine, Samantha Reed, James K Fredrickson, Kenneth H Nealson, Andrei L Osterman

**Affiliations:** 1 Burnham Institute for Medical Research, La Jolla, California 92037, USA; 2 Institute for Information Transmission Problems, Russian Academy of Sciences, Moscow 127994, Russia; 3 Key Laboratory of Synthetic Biology, Institute of Plant Physiology and Ecology, Shanghai Institutes for Biological Sciences, Chinese Academy of Sciences, Shanghai 200032, China; 4 Department of Earth Sciences, University of Southern California, Los Angeles, California 90089, USA; 5 Fellowship for Interpretation of Genomes, Burr Ridge, Illinois 60527, USA; 6 Biological Sciences Division, Pacific Northwest National Laboratory, Richland, Washington 99352, USA; 7 J. Craig Venter Institute, San Diego, California 92121, USA

## Abstract

**Background:**

Carbohydrates are a primary source of carbon and energy for many bacteria. Accurate projection of known carbohydrate catabolic pathways across diverse bacteria with complete genomes constitutes a substantial challenge due to frequent variations in components of these pathways. To address a practically and fundamentally important challenge of reconstruction of carbohydrate utilization machinery in any microorganism directly from its genomic sequence, we combined a subsystems-based comparative genomic approach with experimental validation of selected bioinformatic predictions by a combination of biochemical, genetic and physiological experiments.

**Results:**

We applied this integrated approach to systematically map carbohydrate utilization pathways in 19 genomes from the *Shewanella *genus. The obtained genomic encyclopedia of sugar utilization includes ~170 protein families (mostly metabolic enzymes, transporters and transcriptional regulators) spanning 17 distinct pathways with a mosaic distribution across *Shewanella *species providing insights into their ecophysiology and adaptive evolution. Phenotypic assays revealed a remarkable consistency between predicted and observed phenotype, an ability to utilize an individual sugar as a sole source of carbon and energy, over the entire matrix of tested strains and sugars.

Comparison of the reconstructed catabolic pathways with *E. coli *identified multiple differences that are manifested at various levels, from the presence or absence of certain sugar catabolic pathways, nonorthologous gene replacements and alternative biochemical routes to a different organization of transcription regulatory networks.

**Conclusions:**

The reconstructed sugar catabolome in *Shewanella *spp includes 62 novel isofunctional families of enzymes, transporters, and regulators. In addition to improving our knowledge of genomics and functional organization of carbohydrate utilization in Shewanella, this study led to a substantial expansion of our current version of the Genomic Encyclopedia of Carbohydrate Utilization. A systematic and iterative application of this approach to multiple taxonomic groups of bacteria will further enhance it, creating a knowledge base adequate for the efficient analysis of any newly sequenced genome as well as of the emerging metagenomic data.

## Background

Carbohydrates comprise a key natural source of carbon and energy for a variety of heterotrophic microbes. The diversity of carbohydrates (polymers, oligo- and mono-saccharides) in various ecosystems as well as the diversity of microbial lifestyles is reflected in substantial variations of sugar catabolic machinery even between phylogenetically closely-related microorganisms. An ability to confidently reconstruct this machinery directly from genomic sequence is crucial to an understanding of microbial ecophysiology, evolution, adaptation, and even interactions between microorganisms and their animal, insect, or plant hosts. However, despite many studies, the bulk of our knowledge about sugar utilization pathways is limited to a handful of model bacteria such as *E. coli*. An accurate projection of this knowledge across the growing variety of divergent bacteria with completely sequenced genomes constitutes a substantial challenge. This is largely due to the aforementioned variations including numerous cases of non-orthologous gene replacements, families of paralogous proteins with varying substrate specificity as well as alternative and presently unknown biochemical routes. Due to this complexity, genomic annotations of carbohydrate utilization genes derived solely from sequence similarity analysis are often imprecise and incomplete, especially for relatively distant and poorly studied bacteria.

Shewanellaceae are a versatile family of Gram-negative γ-proteobacteria that have adapted for survival in highly varied aquatic and sedimentary environments that exhibit extremes in salinity, temperature, redox chemistry, and hydrostatic pressure [1]. Although *Shewanella *can respire with a diverse array of electron acceptors that include organic compounds and metal oxides [2], they were thought to possess a relatively narrow capacity for utilizing electron donors, preferring simple carbon compounds such as formate, lactate, and acetate. The recent results from comparative and functional genomic analysis [3], physiological experiments [4] and microarray analysis [5] demonstrated that *S. oneidensis *MR-1 can use a wide array of complex carbon compounds including nucleotides, amino acids, mono- and polysaccharides.

A subsystems-based approach to genome analysis allows us to substantially improve the accuracy of genomic annotations and to predict functions of previously unknown gene families [6,7]. This approach includes a combination of comparative genomic techniques such as the analysis of conserved operons and regulons, with pathway reconstruction across a large variety of genomes. Recently, we used a subsystems-based approach to predict and experimentally verify novel pathways of *N-*acetylglucosamine and lactate utilization in *Shewanella *[8,9]. In the current study we have expanded this analysis towards genomic reconstruction of the entire repertoire of carbohydrate utilization pathways in a group of 19 species from the genus *Shewanella *with completely sequenced genomes.

## Results

### Reconstruction of carbohydrate utilization machinery in *Shewanella *spp

To address the challenge of genomic reconstruction of the carbohydrate utilization machinery we took advantage of several features characteristic of many sugar utilization pathways, such as:

#### Uniform functional organization

A typical sugar utilization pathway includes a *transport system *for sugar uptake and a set of intracellular enzymes that perform *biochemical transformations*. Although the latter processes vary from sugar to sugar (and, to a degree, from organism to organism), most of them are performed by a rather narrow spectrum of enzymatic activities such as kinases, isomerases, oxidoreductases, hydrolases, and aldolases. Sugar kinases or phosphoenolpyruvate:sugar phosphotransferase systems (PTS) catalyze an essential phosphorylation step (as all intermediates of central carbon pathways to the point of pyruvate are anchored by one or two phosphates). We also included in this analysis associated *transcriptional regulators *of sugar utilization pathways that mediate specific induction of a catabolic pathway. For utilization of various natural polysaccharides, many pathways are equipped by upstream hydrolytic enzymes producing oligo-, di- or mono-saccharides that are then transported into the cell and further metabolized. These and other *upstream and auxiliary *(e.g. involved in sensing and chemotaxis) *components *of the carbohydrate utilization machinery were typically excluded from our analysis, except for those that share genomic context with other sugar utilization components.

#### Ubiquitousprotein families

Despite their functional diversity, many characterized components of sugar utilization pathways occur in a limited number of protein families containing multiple paralogs with varying substrate specificities. Representatives of these families can be recognized by homology-based genomic searches, thus providing a source of gene candidates for pathway reconstruction. On the other hand, homology-based methods, taken alone, often fail to reliably identify the exact substrate specificity. This can be accomplished by additional reasoning based on the analysis of functional and genomic context (functional coupling) [10].

#### Strong functional coupling

Bacterial genes involved in sugar catabolism are often organized in compact operons and/or regulated by committed transcription factors. Therefore, genome context analysis techniques [6] based on the identification of conserved chromosomal clusters (operons) and shared regulatory sites (regulons) are particularly efficient for accurate functional assignment of previously uncharacterized genes of sugar utilization pathways.

**Figure 1:**
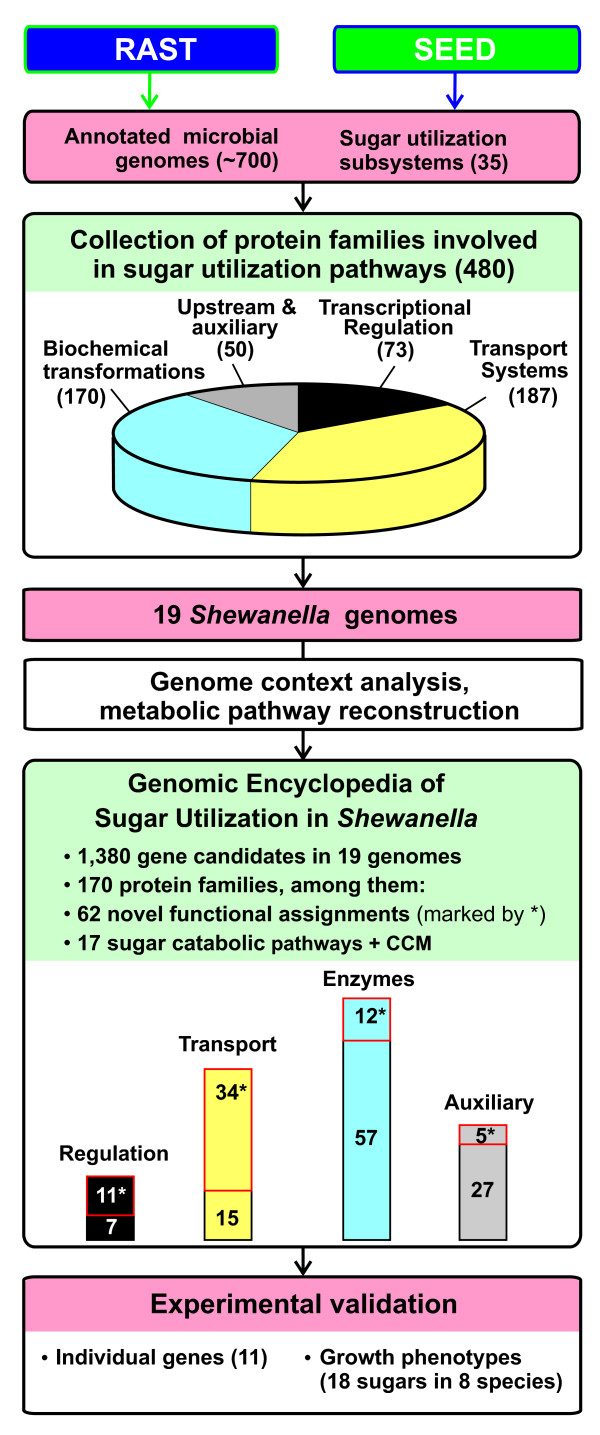
**Workflow for genomic reconstruction of carbohydrate utilization machinery in *Shewanella *spp**.

Based on the above considerations we developed the bioinformatic workflow that was applied for the analysis of 19 complete *Shewanella *genomes (Fig. 1). The key starting point of this workflow was a collection of manually curated subsystems in the SEED genomic database [7] capturing a substantial fraction of known sugar utilization pathways projected across many bacterial species. A compilation of ~480 groups of isofunctional homologs from this collection (see additional data file 1) was used as a source of queries for homology-based scanning of 19 *Shewanella *genomes integrated in the SEED database. The underlying assumption was that any sugar utilization pathway, even extremely divergent or fully unknown, should contain at least one protein recognizably homologous to some of the query sequences from this collection. Therefore, all of the revealed homologs would be considered potential "kernels" of sugar utilization pathways, even though a specific function of some of them might be unclear (and distinct from the query). During further analysis many of the initially identified gene candidates whose functional roles were deemed unrelated to sugar utilization (e.g. involved in biosynthetic pathways) or remained dubious (lacking any evidence for functional assignment) were rejected.

By applying this workflow we tentatively defined the first draft of the Genomic Encyclopedia of Sugar Utilization (or, shortly, sugar catabolome) in 19 *Shewanella *spp as comprised of 170 isofunctional protein families (FIGfams) [11]. The complete list and some statistics of the analyzed genomes are provided in Table 1. The detailed results of this analysis are captured in the SEED subsystem 'Sugar catabolome in *Shewanella *species' available online http://theseed.uchicago.edu/FIG/subsys.cgi and summarized in additional files 2 and 3. Functional annotation of the identified protein families was conducted using simultaneous genome context analyses, and homology searches combined with the metabolic reconstruction and identification of missing steps in the putative catabolic pathway.

**Table 1:** Genomic properties and isolation site characteristics of analyzed *Shewanella *species.

Genome	Alias	**Total genes**^**1**^	**Sugar genes**^**2**^	Sugar **pathways**^**3**^	Isolation site characteristics
*S.oneidensis *MR-1	MR1	4318	56	4	Lake Oneida, NY, USA (sediment)
*S.putrefaciens *CN-32	CN32	3972	64	4	Albuquerque, NM, USA (subsurface)
*S.putrefaciens *W3-18-1	W3181	4044	64	4	Washington Coast, Pacific Ocean (sediment)
*S*.sp. ANA-3	ANA3	4111	87	7	Eel Pond, Woods Hole, MA, USA (brackish water)
*S*.sp. MR-4	MR4	3924	86	7	Black Sea (sea water - 5 m)
*S*.sp. MR-7	MR7	4006	92	8	Black Sea (sea water - 60 m)
*S.baltica *OS155	Sbal	4307	77	7	Baltic Sea (sea water - 90 m)
*S.baltica *OS185	OS185	4323	84	8	Baltic Sea (sea water - 120 m)
*S.baltica *OS195	OS195	4499	80	7	Baltic Sea (sea water - 140 m)
*S.baltica *OS223	OS223	4250	110	10	Baltic Sea (sea water - 120 m)
*S.denitrificans *OS217	Sden	3754	58	4	Baltic Sea (sea water - 120 m)
*S.loihica *PV-4	PV4	3859	59	5	Loihi Seamount, Pacific Ocean (hydrothermal vent)
*S.amazonensis *SB2B	Sama	3645	89	6	Amazon River Delta, Brazil (sediment)
*S.frigidimarina *NCIMB 400	Sfri	4029	70	8	North Sea, coast of Aberdeen, UK (sea water)
*S.piezotolerans *WP-2	Spie	4933	68	6	West Pacific site (sediment - 1914 m)
*S.pealeana *ANG-SQ1	Spea	4241	71	6	Woods Hole harbor, MA, USA (squid gland)
*S.halifaxens *HAW-EB4	Shal	4278	59	5	Halifax Harbor, Nova Scotia, CA (sediment - 215 m)
*S.sediminis *HAW-EB3	Ssed	4497	47	3	Halifax Harbor, Nova Scotia, CA (sediment - 215 m)
*S.woodyi *ATCC 51908	Swoo	4880	84	7	Strait of Gibraltar, Mediterranean Sea (370 m)

^1 ^protein coding genes

^2 ^number of genes involved in the reconstructed sugar utilization pathways.

^3 ^number of the reconstructed sugar utilization pathways.

**Figure 2:**
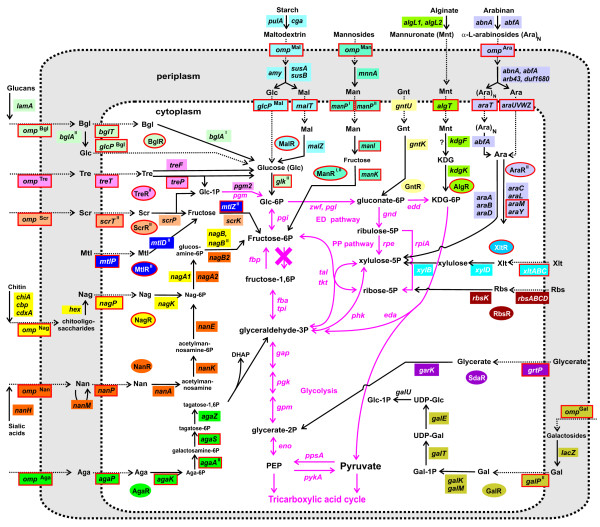
**Reconstructed pathways of carbohydrate utilization in *Shewanella *pan-genome**. Abbreviations for carbohydrates are listed in Table 2. Functional roles implicated in the same catabolic pathway are shown by matching background colors. Novel functional roles predicted in this work are in red boxes. Enzymatic and transport routes are shown by solid and dotted lines, respectively. Transcriptional factors predicted to control sugar utilization pathways are shown in ovals of matching colors. Reactions and enzymes of the central carbohydrate metabolism are shown in magenta. Note that none of the individual *Shewanella *species contain all (or even most) of the shown pathways.

Specific functional assignments have been proposed for 157 FIGfams comprising 17 peripheral sugar utilization pathways and the key components of the central carbon metabolism (CCM) pathways (Table 2, Fig. 2). A total of 21 FIGfams involved in CCM upstream of pyruvate are conserved in all analyzed *Shewanella *spp. They comprise complete canonical pentose phosphate (PP) and Entner-Doudoroff (ED) pathways. However, an essential enzyme of glycolysis, 6-phosphofructokinase (Pfk), is missing in all of these species suggesting that sugar utilization in *Shewanella *can proceed only through the ED or PP pathways. This is in agreement with previous metabolic reconstruction reports [1,3] and biochemical studies of CCM enzymes in cell extracts of certain *Shewanella *strains [12]. All other canonical enzymes of glycolysis/gluconeogenesis are present in all analyzed *Shewanella *genomes pointing to the presence of intact gluconeogenetic route. The peripheral pathways are comprised of 136 FIGfams showing a mosaic distribution among 19 compared species. The total number of proteins that comprise the peripheral sugar utilization machinery of individual species varies broadly, from 18 proteins comprising 3 pathways in *S. sediminis *up to 74 proteins comprising 10 pathways in *S. baltica *OS223.

**Table 2:** Reconstructed sugar utilization pathways and newly assigned genes in *Shewanella *spp.

Sugar utilization pathway (subsystem)		Number of genomes	Number of genes	Newly assigned genes			
			
Carbohydrates	**Abbrev**.			regulation	transport	enzymes	auxiliary
Central carbon metabolism	CCM	19	21	-	-	-	-
*N-*acetylglucosamine, chitin	Nag	18	15	*nagR*	***nagP***, *omp*^*Nag*^	***nagB***^*II*^, ***nagK*,**	*mcp^Nag^, nagX*
Glycerate	Grt	17	3	-	***grtP***	-	-
β-glucosides, cellobiose	Bgl	9	8	*bglR*	***bglT***, *glcP*^*Bgl *^*omp*^*Bgl*^	***glk***^*II*^	-
Sucrose	Scr	8	5	*scrR*^II^	***scrT*^II^***, omp*^Scr^	-	-
Maltodextrins	Mal	14	13	*malR*	*malT*, ***glcP*****^Mal ^***omp*^Mal^(2)	-	-
Arabinose, arabinosides	Ara	6	18	*araR*^II^	*araUVWZ, araT, omp*^Ara^	*araM, araY*	*araX*
Galactose, galactosides	Gal	8	10	-	*galP*^II, ^*omp*^Gal^	-	-
Gluconate	Gnt	4	3	-	-	-	-
*N-*acetylgalactosamine	Aga	4	8	-	*agaP, omp*^Aga^	*agaA*^II^*, agaK, agaS*	*agaO*
Mannosides	Man	4	14	*manR*^I^*, manR*^II^	*manP*^I^*, manP*^II^*, omp*^Man^	*manI, manK*	-
Trehalose	Tre	3	6	*treR*^II^	*treT, omp*^Tre^	*treP*	-
Xylitol	Xlt	2	6	*xltR*	*xltABC*	-	-
Ribose	Rbs	2	6	-	-	-	-
Sialic acids	Nan	1	10	-	*nanP, omp*^Nan^	-	-
Alginate	Alg	1	6	*algR*	*algT*	-	-
Mannitol	Mtl	1	5	*mtlR*^II^	*mtlP*	*mtlZ*^II^	*mtlX*
Unassigned	-	19	13	-	-	-	-
**Total number:**	**17**	**19**	**170**	**11**	**34**	**12**	**5**

Predicted novel functional assignments verified by targeted experiments are marked by bold type and underlined. The details of all functional assignments summarized in this table are provided in additional files 2 and 3.

13 FIGfams have not been assigned a specific function in any sugar utilization pathway due to the lack of any suggestive genomic context (see additional data files 2 and 3). Among them there are three genes encoding putative sugar kinases of unknown specificity, and genes from a hypothetical sugar utilization gene cluster (for more details see additional data file 4).

Using the genome context analysis combined with metabolic reconstruction we inferred specific functional assignments for 62 families of isofunctional homologs whose functions were previously unknown or defined only at the level of general class (Table 2, see also additional file 5). The novel functional assignments, including 34 components of transport systems, 11 transcriptional regulators (and corresponding DNA motifs), 12 metabolic enzymes, and 5 auxiliary proteins, are supported by the overall internal consistency of the entire reconstruction where all of the pathways are complete (no gaps or missing genes), and all of the annotated genes are associated with complete pathways (no genes out of context). Selected functional assignments mostly for novel sugar transporters from those pathways that are present in most of the analyzed *Shewanella *species were tested by targeted biochemical and genetic experiments (for details see next section and additional file 6). An ultimate validation of the entire reconstruction was attained by the experimental testing of growth phenotypes (an ability of a given strain to grow on a specific sugar substrate as a sole source of carbon and energy) predicted by the presence or absence of respective reconstructed pathways. The pathway presence was defined based on the presence of all components of the pathway in a particular genome. This phenotype profiling was performed using a "matrix" approach where 14 representative *Shewanella *species were profiled against a panel of 18 diagnostic sugar substrates (Table 3).

**Table 3:** Consistency between the predicted and experimentally determined growth phenotypes of *Shewanella *spp.

Strain	Glc	Nag	Grt	Bgl	Scr	Mal	Ara	Gal	Gnt	Aga	Tre	Mtl
MR1	+/n*	+/p	+/p	-/n	-/n	+/n*	-/n	-/n	-/n	-/n	-/n	-/n
CN32	-/n	+/p	+/p	-/n	-/n	-/n	+/p	-/n	-/n	-/n	-/n	-/n
W3181	-/n	+/p	+/p	-/n	-/n	-/n	+/nd	-/n	-/nd	-/nd	-/n	-/nd
OS155	+/p	+/p	+/p	+/p	+/p	+/p	-/n	-/n	+/p	-/n	-/n	-/n
OS185	+/p	+/p	+/p	+/p	+/p	+/p	-/nd	+/p	+/nd	-/nd	-/n	-/nd
OS195	+/p	+/p	+/p	+/p	+/p	+/p	-/nd	-/n	+/nd	-/nd	-/n	-/nd
OS223	+/p	+/p	+/p	+/p	+/p	+/p	-/nd	+/p	+/nd	-/nd	+/p	-/nd
MR7	+/p	+/p	+/p	-/n	+/p	+/p	+/p	-/n	-/n	+/p	-/n	-/n
MR4	+/p	+/p	+/p	-/n	+/p	+/p	+/p	-/n	-/n	+/p	-/n	-/n
ANA3	+/p	+/p	+/p	-/n	+/p	+/p	+/p	-/n	-/n	+/p	-/n	-/n
Sden	+/w	+/p	-/n	+/p	-/n	+/p	-/n	-/n	-/n	-/n	-/n	-/n
Sfri	+/p	-/n	+/p	+/p	+/p	+/p	-/n	-/n	-/n	-/n	+/p	+/n*
PV4	+/w	+/p	+/p	-/n	-/n	+/w	-/n	+/w	-/n	-/n	-/n	-/n
Sama	+/p	+/p	-/n	+/p	-/n	+/p	-/n	-/n	-/n	+/p	-/n	-/n

Aliases for analyzed *Shewanella *strains are described in Table 1. Each cell in the table combines the data on the predicted and experimentally determined sugar utilization phenotype. The ability of *Shewanella *species to grow on a panel of sugar substrates was predicted based on the presence (+) or absence (-) of the respective reconstructed pathways in their genomes. The experimental results of growth experiments from this study (see additional data files 7 and 8) are: 'p', positive growth; 'w', weak growth; 'n', no growth. Inconsistencies between the predicted and experimentally determined growth phenotypes are in bold and marked by asterisk.

### Peripheral sugar utilization pathways

In this section we describe the key novel aspects of eight reconstructed sugar utilization pathways that were found to be relatively widely distributed in *Shewanella *genus. The "core" subset includes pathways for utilization of D-glucose, *N-*acetylglucosamine, D-glycerate, β-glucosides (present in 14 or more species). The "intermediate" subset includes the sucrose, maltodextrins, D-galactose, and L-arabinose catabolic pathways (conserved in 6 or more species). We also provide the results of experimental validation of selected novel functional roles in five sugar catabolic pathways. The details of the other nine reconstructed pathways for utilization of alginate, gluconate, mannitol, mannosides, *N-*acetylgalactosamine, ribose, sialic acids, trehalose, and xylitol that were found only in one to four *Shewanella *strains ("rare" pathways) are described in the additional file 4. We also report the results of growth phenotype characterization for most of the respective sugar substrates.

#### D-glucose

**Figure 3:**
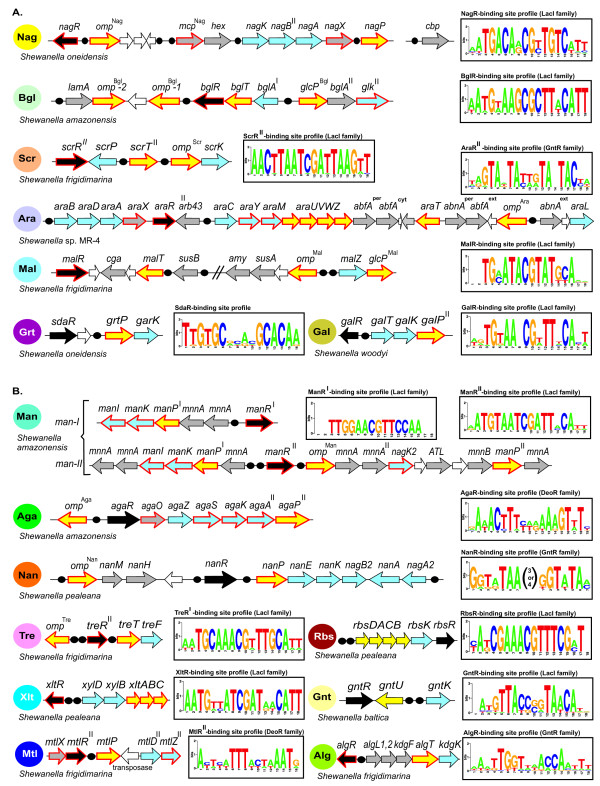
**Genomic context and regulons of genes involved in sugar utilization in representative *Shewanella *genomes**. Panel A contains metabolic pathways discussed in the main text as the "core" and intermediate" subsets. Panel B contains "rare' pathways described in the additional file 4. Genes are colored by their functional classification: sugar transport systems, yellow; cytoplasmic enzymes catalyzing biochemical transformations, blue; transcriptional regulators, black; extracytoplasmic sugar hydrolytic enzymes and auxiliary proteins functionally linked to the pathway, gray. Genes with novel functional roles predicted in this work are outlined in red. For each sugar-specific transcription factor, predicted DNA binding sites are shown by black dots and a consensus DNA-binding motif is shown as a sequence logo.

(Glc) is utilized by bacteria using either (i) hexose permease and glucokinase, or (ii) PTS^Glc ^system. The inability to ferment glucose as a carbon source under aerobic conditions was originally attributed to the *Shewanella *genus, whereas later studies have identified several species that were able to utilize glucose such as *S. baltica *and *S. frigidimarina *[13-15]. In this study we have tentatively identified respective genes, glucose transporters *glcP*^Bgl ^and *glcP*^Mal ^and glucokinase *glk*^II^, that are conserved in most *Shewanella *genomes, where they are clustered on the chromosome with the genes from β-glucoside (Bgl) and maltodextrin (Mal) utilization pathways, respectively (Fig. 3A). These catabolic pathways include several secreted glucosidases (e.g. BglA^II^, CgA, SusB) generating extracytoplasmic glucose that can be utilized via associated glucose transporters and the glucokinase (see below). The predicted glucose transporters belong to the glucose-galactose permease (GGP) family, and are most similar to the glucose and galactose transporter GluP from *Brucella abortus *[16] and fucose permease FucP from *E. coli*. The predicted glucokinase Glk^II ^belongs to the ROK family of sugar kinases [17], and is similar to fructokinase Mak, D-allose kinase AlsK, and *N-*acetylglucosamine kinase NagK from *E. coli*, and glucokinase GlcK from *B. subtilis*. Orthologs of both *glcP*^Mal^/*glcP*^Bgl ^and *glk*^II ^are absent from *E. coli *and other Enterobacteria. The GlcP^Bgl ^and GlcP^Mal ^transporters in *Shewanella *were found only in the context of the Bgl and Mal pathways, whereas Glk^II ^seems to play a general housekeeping role being conserved in all analyzed *Shewanella *genomes (see additional data files 2 and 3). In 9 of 19 genomes *glk*^II ^was also found within the Bgl utilization gene cluster, and in 6 of these cases it is a second copy of the gene in the genome.

To confirm the functional assignment of GlcP, we constructed the Δ*glcP*^Mal ^knockout mutant in *Shewanella *sp. ANA-3, and tested it for glucose-dependent growth. In contrast to the wild-type ANA-3, the Δ*glcP*^*Mal *^strain was unable to grow on D-glucose as a sole carbon and energy source, confirming the glucose transporter functional assignment (see additional data file 6 for original experimental data). A representative of the predicted glucokinase subfamily (Glk^II^) from *S. baltica *OS155 was experimentally characterized as a part of our analysis of the Bgl pathway (see below). To assess the functionality of the predicted glucose catabolic pathway we performed phenotypic characterization of *Shewanella *for growth on D-glucose as a sole carbon and energy source (see additional data files 7 and 8 for the original physiological growth data). Among 14 *Shewanella *strains tested only *S. oneidensis *and two *S. putrefaciens *strains were unable to grow on glucose. These results are consistent with the distribution of *glcP *transporters in these *Shewanella *genomes (Table 3, see also additional file 2). The inability of *S. oneidensis *MR-1 to grow on glucose is most likely due to a frameshift in the *glcP*^*Mal *^gene[18].

The second (PTS-driven) route of glucose utilization is not expected to support the growth of bacteria (such as *Shewanella *spp) with the incomplete glycolytic pathway due to the inability of the ED or PP pathways to generate enough phosphoenolpyruvate to compensate for its consumption in the phosphotransferase reaction. This assertion is supported by the observed impaired growth of the *E. coli pfk *mutant on glucose using PTS^Glc ^[19]. Therefore, the actual physiological role of *Shewanella *genes orthologous to the components of the *E. coli *PTS^Glc ^system (*ptsHI-crr *and *ptsG*) remains a mystery. It would be tempting to speculate that this PTS system is used by *Shewanella *for glucose consumption in the presence of other substrates contributing to nonglycolytic phosphoenolpyruvate generation. However, our attempts to grow MR-1 on the mixture of lactate and glucose did not confirm this conjecture (data not shown).

#### N-acetylglucosamine

(Nag) and chitin catabolic pathway (Fig. 2, see also additional file 9) conserved in most *Shewanella *spp contains several novel functional roles (Table 2). Previously we have experimentally confirmed the predicted enzymatic activities of novel Nag kinase (NagK) and glucosamine-6-phosphate deaminase (NagB^II^) and reconstituted the entire three-step biochemical conversion of Nag to fructose-6-phosphate *in vitro *[9]. We have also confirmed that all of the tested *Shewanella *strains can grow on Nag as a single source of carbon and energy with the exception of *S. frigidimarina*, which does not contain these genes (Table 3). Here we present additional experimental results supporting functional assignments of the predicted transporter NagP in *S. oneidensis *MR-1. NagP belongs to the GGP family of transporters and has a limited similarity to the glucose permeases GlcP^Bgl ^and GlcP^Mal^. We have constructed the *S. oneidensis *Δ*nagP *targeted deletion mutant and demonstrated the loss of its ability to grow on Nag (see additional data file 6).

The Nag catabolic genes shows their wide distribution in the Alteromonadales lineage, suggesting the presence of the Nag pathway in the common ancestor of this lineage. The absence of Nag pathway in *S. frigidimarina *is attributed to a loss of the entire chromosomal gene cluster. The observed wide distribution of Nag utilization pathway in *Shewanella *could be related to their ability to utilize chitin, a highly abundant constituent of aquatic invertebral exoskeleton.

#### D-glycerate

(Grt) utilization pathway in *Shewanella *species involves D-glycerate kinase GarK, transcriptional regulator SdaR, and a novel D-glycerate permease termed here GrtP (Fig. 2, see also additional file 9). In *E. coli*, GarK is involved in the glucarate/galactarate catabolic pathway, generating 2-phosphoglycerate as product, and SdaR is a common transcriptional regulator of this pathway [20]. Most of the glucarate/galactarate utilization genes are absent in *Shewanella *species. The only exception is a *garK *gene ortholog, which was found in 17 *Shewanella *strains in a conserved operon with a hypothetical gene encoding the predicted D-glycerate transporter GrtP (see additional data files 2 and 3). The *grtP-garK *operon is clustered on the chromosome with an *sdaR *gene ortholog. A comparative genomic reconstruction of the SdaR regulon in γ-proteobacteria allowed us to predict a candidate SdaR-binding site located upstream of the *grtP-garK *operon in *Shewanella *genomes (Fig. 3A).

The predicted D-glycerate uptake transporter GrtP belongs to the gluconate permease family and has orthologs in Pseudomonadales and Vibrionales but not in *E. coli *or other Enterobacteria. The predicted function of GrtP was confirmed by the loss of ability of the *S. oneidensis *Δ*grtP *strain to grow on D-glycerate as a sole carbon and energy source (see additional data file 6). Of the 14 *Shewanella *strains, all but 2 (*S. denitrificans *and *S. amazonensis*) could grow on D-glycerate (see additional data files 7 and 8), which is fully consistent with the distribution of the *grtP-garK *operon among the analyzed *Shewanella *genomes (Table 3). The natural source of D-glycerate in aquatic environments is yet to be elucidated.

#### β-glucoside

'Bgl' utilization pathway revealed in 9 *Shewanella *species involves β-glucanase LamA, two β-glucosidases BglA^I ^and BglA^II^, two novel β-glucoside transporters BglT and Omp^Bgl^, a novel LacI-type transcriptional regulator BglR, glucose permease GlcP^Bgl ^and ROK-type glucokinase Glk^II ^(Fig. 2, see additional data files 2, 3 and 9). The previously described Bgl catabolic pathways use PTS-type transport systems and 6-phospho-β-glucosidases (as in *B. subtilis *and *E. coli*) or ABC-type transport systems and β-glucosidases (as in *Streptomyces *spp and Archaea) [21]. The predicted β-glucoside uptake transporter BglT is a member of the glycoside-pentoside-hexuronide (GPH) transporter family, and has orthologs in other Alteromonadales species. Homologs of BglT in Enterobacteria (e.g. YicJ and YagG from *E. coli *that show 40-44% sequence similarity to BglT) are predicted xyloside transporters regulated by the xylose activator XylR [22]. Omp^Bgl ^belongs to the TonB-dependent outer membrane transporter (TBDT) family, which includes proteins involved in high-affinity binding and energy-dependent uptake of various substrates (including oligosaccharides) into the periplasm [23]. The predicted Bgl-specific transporter Omp^Bgl ^is a nonorthologous replacement of the Bgl-specific outer membrane porin BglH from *E. coli*. Comparative genomic reconstruction of the BglR regulon allowed us to predict candidate BglR-binding sites located upstream of the divergently transcribed *bglA^I^-bglT-bglR *and *glcP*^*Bgl*^*-bglA*^*II*^operons, and upstream of the *omp*^*Bgl *^gene (Fig. 3A). Thus the predicted novel transporters are positionally clustered and co-regulated with other Bgl catabolic genes.

The reconstructed Bgl pathway in *Shewanella *involves three glucosidases, two of which, LamA and BglA^II^, are predicted to be secreted outside of the cell and to the periplasm, respectively, whereas BglA^I ^is likely a cytoplasmic enzyme. We propose that β-glucoside-containing glucans are first degraded by extracellular endo-β-1,3-glucanase LamA, the resulting oligo-β-glucosides are transported to the periplasm by Omp^Bgl^, and subsequently utilized by BglA^II ^to produce D-glucose and shorter β-glucosides (e.g., cellobiose, gentibiose). The latter products are taken up by the predicted BglT transporter into the cytoplasm where they are finally hydrolyzed by the BglA^I ^enzyme. D-glucose is taken up by the predicted glucose transporter GlcP^Bgl ^and phosphorylated by the Glk^II ^glucokinase (Fig. 2).

The newly predicted β-glucoside BglT transporter was experimentally confirmed by genetic complementation in *E. coli *Δ*bglF *mutant (a knockout of cellobiose PTS system). This strain, when transformed by an expression vector containing an operon *bglA^I^*- *bglT *cloned from *S. baltica *OS155 gained the ability to grow on minimal medium with cellobiose as the sole carbon and energy source. In the same conditions, no growth was observed for this strain transformed by vector only or by the plasmids containing single genes, *bglA^I ^*or *bglT *(see additional data file 6).

The predicted glucokinase activity of the *S. baltica *OS155 *glk*^II ^gene product was confirmed by an *in vitro *enzyme assay. Recombinant purified Glk^II ^exhibited a broad substrate specificity at 37°C with the highest activity with D-glucose (22 μmol/mg/min), and an appreciable activity with some other tested hexoses including D-mannose, D-fructose, D-glucosamine, and D-mannosamine (see additional data file 6).

The results of the phenotype profiling of 14 *Shewanella *strains showed that only half of them (four *S. baltica *species, *S. amazonensis*, *S. frigidimarina*, and *S. denitrificans*) were able to grow on cellobiose as a sole carbon and energy source (see additional data files 7 and 3). This result is consistent with the distribution of the respective genes in the analyzed *Shewanella *genomes (Table 3).

**Figure 4:**
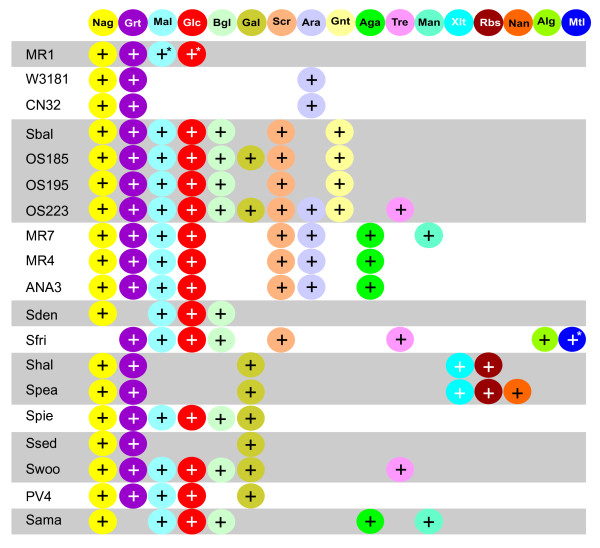
**Distribution of 17 sugar utilization pathways encoded in 19 *Shewanella *genomes**. *Shewanella *species abbreviations are as described in Table 1. Asterisks indicate cases when a pathway is impaired by the presence of an insertion sequence element or a frameshift mutation and, thus, deemed nonfunctional.

The Bgl catabolic genes are sparsely distributed among *Shewanella *species (Fig. 4) and many other species from the Alteromonadales lineage. Therefore, it might be an ancestral pathway predating the speciation of Alteromonadales that could have been independently lost several times within the *Shewanella *genus. The species-specific loss of Bgl pathway by many *Shewanella *could be linked to a particular ecophysiology of their habitat. For example, *S. pealeana *which colonizes the accessory nidamental gland of the squid may not have access to Bgl substrate and thus lost the respective gene cluster from its chromosome [15].

#### Sucrose

(Scr) utilization pathway in 8 *Shewanella *species involves sucrose phosphorylase ScrP, fructokinase ScrK, two novel sucrose transporters (ScrT^II ^and Omp^Scr^), and a novel LacI-type transcriptional regulator ScrR^II ^(Fig. 2; see also see additional data files 2, 3 and 9). This pathway variant is quite different from the canonical pathway known in Enterobacteria, which includes the sucrose-specific PTS-driven uptake and phosphorylation followed by sucrose-6-phosphate hydrolase [24]. An alternative variant of the Scr pathway previously described in *Bifidobacterium *spp includes sucrose permease ScrT from the MelB melibiose transporter family and sucrose phosphorylase [25]. The major disctinction in *Shewanella *is a nonorthologous replacement of ScrT by a predicted transporter ScrT^II ^of the GGP family of sugar transporters. The novel sucrose regulator ScrR^II ^in *Shewanella *is another nonorthologous replacement of the previously characterized ScrR repressor from other bacteria. A genomic reconstruction of the ScrR^II ^regulon allowed us to predict its candidate binding sites upstream of the divergently transcribed *scrP *and *scrT^II ^*genes, as well as upstream of the *omp^Scr^-scrK *operon (Fig. 3A). The predicted Scr-specific TBDT Omp^Scr ^is functionally equivalent to the Scr-specific outer membrane porin ScrY from Enterobacteria, and is presumably involved in the uptake of sucrose into the periplasm[26].

The ScrT^II ^transporter was validated by complementation in *E. coli *K-12, which lacks the Scr pathway and is unable to utilize sucrose (see additional data file 6). Two divergently transcribed genes from *S. frigidimarina*, *scrT^II ^*and *scrP*, were expressed in *E. coli *under the control of an endogenous promoter in their common intergenic region. As a negative control, we used an empty vector as well as single *scrT^II ^*or s*crP *genes expressed in the same strain. The cell growth was monitored in a minimal medium with sucrose as the only carbon and energy source. We have found that only the cells carrying both *scrT^II ^*and s*crP *were able to grow on sucrose providing an experimental verification of the reconstructed Scr utilization pathway.

The growth phenotype profiling of 14 *Shewanella *species demonstrated that eight of them (*Shewanella *sp. ANA-3, MR-4, MR-7, four *S. baltica *strains, *S. frigidimarina*) are able to grow on sucrose (see additional data files 7 and 8). These results are in agreement with the presence of the respective genes in these 8 *Shewanella *genomes (Table 3).

The Scr catabolic genes are present in a large group of *Shewanella *species (Fig. 4) and in some other species from the Alteromonadales lineage. The most parsimonious scenario suggests that the Scr pathway was present in a common ancestor of the *Shewanella *genus and that it has been independently lost several times in the evolutionary history of *Shewanella*. Distribution of the Scr pathway among the *Shewanella *species provides another possible link to their ecophysiology, the availability of plant-derived sucrose in their respective ecologic niches.

#### Maltodextrin

(Mal) utilization gene locus identified in 14 *Shewanella *species contains the *amy*, *cga*, *susA*, *susB*, and *malZ *genes encoding orthologs of previously characterized starch and maltodextrin hydrolytic enzymes, as well as genes encoding novel transporters termed MalT, GlcP^Mal ^and Omp^Mal^, and a novel LacI-type transcriptional regulator MalR (see additional data file 2). The reconstructed Mal catabolic pathway in *Shewanella *(Fig. 2) uses a predicted novel sodium:solute symporter MalT (a homolog of the melibiose permease MelB of *E. coli*) instead of the maltose ABC transporter MalEFGK previously described in *E. coli *[27]. The outer membrane maltose/maltodextrin uptake in *Shewanella *is presumably mediated by a novel TBDT Omp^Mal^, the functional equivalent of *E. coli *maltoporin LamB[26]. The novel glucose transporter GlcP^Mal ^is a close paralog (67% identity) of the predicted glucose transporter GlcP^Bgl ^from the Bgl utilization pathway in *Shewanella*. We propose that D-glucose is produced in the periplasm by the action of maltodextrin α-glucosidases and transported into the cytoplasm by GlcP^Mal^. A comparative genomic reconstruction of the MalR regulon allowed us to predict candidate MalR-binding sites located upstream of the divergently transcribed *omp*^Mal ^and *malZ-glcP*^Mal ^operons, as well as upstream of the *susB *and *malT-cga *operons (Fig. 3A).

The growth phenotype characterization of 14 *Shewanella *species demonstrated that 11 of them (all except MR-1 and two *S. putrefaciens *strains) are able to grow on maltodextrin as a sole carbon and energy source (see additional data files 7 and 8). These phenotypic results are in agreement with the genomic reconstruction of the Mal pathway (Table 3). The inability of *S. oneidensis *MR-1 to grow on maltodextrin is attributed to significant genetic perturbations within the Mal utilization gene loci (e.g. *glcP*^Mal ^and *susA *are pseudogenes with frameshifts, whereas *omp*^Mal ^is interrupted by an insertion element [18]).

The Mal catabolic genes are widely distributed among *Shewanella *species (Fig. 4) and several other species from the Alteromonadales lineage. Such phyletic pattern suggests the presence of an ancestral pathway in the common ancestor of the *Shewanella *genus and independent species-specific pathway losses. Interestingly, while the Mal pathway in *S. oneidensis *MR-1 demonstrates genetic signs of decay (see above), two closely related *S. putrefaciens *strains have completely lost the Mal pathway genes. The loss of the Mal catabolic genes in some *Shewanella *spp. is most likely caused by a significant shift in available nutrient composition in their habitat, e.g. the absence of plant materials as a source of maltodexrins.

#### L-arabinose

(Ara) and arabinoside utilization gene locus identified in six *Shewanella *species contains more than 20 genes. Nearly half of these genes are similar to previously characterized Ara catabolic genes whereas others are novel (see additional data files 2 and 9). The reconstructed Ara catabolic pathway in *Shewanella *involves the following predicted functional roles: arabinose ABC transporter AraUVWZ, two arabinoside transporters AraT and Omp^Ara^, a novel GntR-type transcriptional regulator AraR^II^, L-arabinose mutarotase AraM, and Ara 1-dehydrogenase AraY (Table 2, Fig. 2).

Two alternative routes of Ara utilization are known in bacteria. The major one present in *E. coli *and *B. subtilis *depends on L-arabinose isomerase AraA, L-ribulokinase AraB, and L-ribulose-phosphate epimerase AraD, whereas the alternative Ara pathway characterized in *Azospirillum brasiliense *proceeds through L-arabinose dehydrogenase, arabinolactonase, and L-arabonate dehydratase [28]. In addition to the *araBAD *genes encoding the conventional Ara utilization pathway through L-ribulose, the Ara metabolic locus in *Shewanella *contains two genes from the alternative Ara pathway, namely L-arabonate dehydratase *araC *and arabinolactonase *araL*, although other genes from the alternative pathway are missing (see additional data file 9). Based on genome context and distant homology analysis we have predicted the gene *araY*, which is similar to D-xylose 1-dehydrogenase from *Caulobacter crescentus *(45% similarity), to be the missing L-arabinose 1-dehydrogenase. A member of aldose 1-epimerase family encoded in the Ara gene cluster was tentatively assigned the functional role Ara mutarotase (AraM), which interconverts alpha and beta anomers of L-arabinose.

The predicted ABC-type arabinose uptake transporter system AraUVWZ is similar to the hypothetical sugar transporter YtfQRST from *E. coli *(58% similarity) and to the ribose transport systems in *E. coli *and *B. subtilis*. The predicted TBDT Omp^Ara ^and the GPH-family transporter AraT are presumably involved in the uptake of arabinosides through the outer and inner membrane, respectively. Comparative genomic reconstruction of the AraR^II ^regulon allowed us to predict its candidate binding sites located in the likely regulatory regions of most operons in the *ara *locus (Fig. 3A). Thus, the predicted novel transporters are both positionally clustered and co-regulated with the other Ara catabolic genes (see additional data files 2 and 3).

The functionality of the predicted arabinose utilization pathway is supported by the growth phenotype profile of *Shewanella *spp on L-arabinose (see additional data files 7 and 8), which correlates perfectly with the presence/absence of the Ara catabolic genes (Table 3).

The Ara catabolic genes are present in a large group of *Shewanella *species (Fig. 4), among which MR-1 and three *S. baltica *strains have lost the complete *ara *gene cluster. Outside of the *Shewanella *genus the most similar *ara *catabolic gene clusters were found in two other γ-proteobacteria, the polysaccharide-degrading marine bacterium *Saccharophagus degradans *and the plant cell wall-degrading soil bacterium *Cellvibrio japonicus*. Arabinose is an important component of plant cell wall. Therefore, the distribution of the Ara catabolic pathway among Shewanella species may also reflect differences in the availability of plant-derived arabinose in their environments.

#### D-galactose

(Gal) metabolism genes encoding galactokinase GalK, mutarotase GalM, UDP-glucose epimerase GalE, and UTP-glucose-1-phosphate uridylyltransferase GalU are conserved in all *Shewanella *genomes (see additional data file 2). Utilization of extracellular galactose or galactosides requires specific transport systems and sugar hydrolases. The *galKM *locus in *S. halifaxensis*, *S. loihica*, *S. pealeana*, *S. piezotolerans*, and *S. sediminis *involves two additional genes, *galP^II ^*and *lacZ*, that are presumably involved in galactose and lactose catabolism. The predicted galactose permease GalP^II ^belongs to the SSF superfamily and is similar to the myo-inositol and glucose transporters from mammals (34% identity). The β-galactosidase LacZ has a candidate signal peptide cleavage site and is presumably a secreted enzyme. We propose that β-galactosides are degraded in the periplasm by LacZ, and the resulting D-galactose residues are taken up by GalP^II ^transporter (Fig. 2). The *omp*^*Gal*^*-lacZ-galTK-galP*^II^*-galE *operon in two *S. baltica *strains (OS185 and OS223) encodes an additional novel transporter from TBDT family, named Omp^Gal^, which is likely involved in lactose uptake through the outer membrane.

The *galTKP^II ^*operon identified in *S. woodyi *is presumably involved in D-galactose monosaccharide utilization, because of the absence of candidate galactoside hydrolase genes in this genome. An ortholog of the transcriptional regulator gene *galR *from *E. coli *is clustered with the *galTKP^II ^*operon in *S. woodyi *and with the *omp*^Gal^*-lacZ-galTK-galP*^II^*-galE *operon in two *S. baltica *strains, and candidate GalR-binding sites were identified in their corresponding upstream regions (Fig. 3).

The growth phenotype characterization of 14 *Shewanella *species demonstrated that only three strains (*S. loihica *PV-4, and two *S.batica *strains) are able to grow on D-galactose (see additional data files 7 and 8). This pattern is consistent with the distribution of the predicted galactose permease gene *galP*^*II *^in the analyzed *Shewanella *genomes (Table 3).

A mosaic distribution of the *galP*^*II *^genes in two groups of the *Shewanella *spp., and the presence of their orthologs in several other marine bacteria from the Alteromonadales lineage suggest multiple gene loss events in the evolutionary history of the Gal catabolic pathway in the *Shewanella *genus. The Gal utilization genes could have been lost in those *Shewanella *species that do not share the same ecological niche with some of the marine animals that are thought to provide a natural source of galactose and β-galactosides.

### Novel sugar utilization pathway variants in *Shewanella*

The reconstructed peripheral pathways in *Shewanella *spp contain 62 variations distinguishing them from those previously described in model species, thus providing a vivid illustration of the aforementioned intrinsic variability of the sugar utilization machinery (see additional data file 5). Most common are numerous cases of nonorthologous gene replacements (corresponding to novel FIGfams), when a functional role is encoded by a gene that is not orthologous (and, in many cases, not homologous) to any of the previously known genes of the same function [29]. In our analysis, such deviations as well as a few cases of alternative biochemical routes were initially recognized as inconsistencies or gaps (*missing genes*) in reconstructed pathways. Such gaps were filled-in by the most likely gene candidates revealed by genome context analysis (e.g. those functionally coupled with canonical genes of the respective pathways via operons and/or regulons). This analysis also allowed us to extend some of the pathways by adding components that would not be commonly perceived as genuine gaps, such as transcriptional regulators and transporters.

Althogh this study is restricted to *Shewanella *spp., most of the newly assigned protein families contain multiple representatives outside the *Shewanella *genus, thus contributing to the reconstruction of respective pathways in a variety of species. It is worth noting that the addition of 62 novel FIGfams led to an appreciable (> 12%) expansion of our original collection of 480 FIGfams associated with sugar utilization subsystems (Fig. 1). Similar analysis applied to new groups of species may reveal additional FIGfams and their combinations involved in the carbohydrate utilization machinery, thus iteratively expanding the entire collection. Of no less importance is the fact that nearly all of the newly assigned genes were originally identified as distant homologs of previously characterized genes with distinct but related functions. For example, a novel Aga kinase was identified as a distant homolog of other known sugar kinases; the novel D-glycerate transporter - as a homolog of *E. coli *D-gluconate permease; the novel BglR repressor - as a homolog of many sugar-specific regulators from the LacI family. Although this observation obviously reflects intrinsic limitations of homology-based predictions, it also delivers a more important message that our knowledge of protein families involved in the carbohydrate utilization is close to saturation at the level of general class functions recognized by homology-based methods. Otherwise, at least some of the gap-filling gene candidates predicted by genome context analysis would have shared no homology with previously known components from the collection. Not a single example of that kind was observed in this study. Moreover, not a single gap has remained in any of the 17 reconstructed sugar utilization pathways clearly supporting the above interpretation.

Characteristic features of the *Shewanella *sugar catabolome contrasted with *E. coli *and other Enterobacteria are described below.

#### Carbohydrate uptake

strategies appear to be strikingly different between *Shewanella *and Enterobacteria. Overall, 34 gene families encoding novel versions of sugar uptake systems were identified in *Shewanella *spp. Most notably, in contrast to an extensive repertoire of 21 PTS systems actively used by *E. coli *for the uptake of various sugars [30], most *Shewanella *species contain only one PTS system of unclear physiological role. As already discussed, the lack of Pfk genes blocks the conventional glycolytic route in *Shewanella *making the ED pathway the only feasible way of hexose utilization. The inability of the latter pathway to sustain energy requirements of PTS systems (due to phosphoenolpyruvate regeneration balance) provides a rationale for their absence in *Shewanella *spp. Thus, the uptake of Glc, Man, Scr, Tre, Nag, and Aga in *E. coli *is mediated by dedicated PTS systems, whereas in *Shewanella *species these sugars are transported by predicted novel permeases of the GGP family, GlcP, ManP, ScrT^II^, TreT, NagP, AgaP^II^, respectively. A combination of inner membrane transporters from the GGP and several other sugar permease families with committed outer membrane transporters of the TBDT family appears to be the predominant strategy of sugar uptake in *Shewanella *(Fig. 2). Committed TBDT outer membrane transporters were mapped for 10 of the 17 reconstructed sugar utilization pathways in *Shewanella *species based on their occurrence within respective operons and regulons (Table 2). The predicted *Shewanella *TBDT transporters are functionally equivalent to sugar-specific outer membrane porins of Enterobacteria (e.g. BglH, ScrY, LamB, NanC). However, in contrast to porins mediating transport across the outer membrane by passive diffusion, TBDT transporters employ a unique energizing mechanism utilizing the TonB complex and the proton-motive force of the cytoplasmic membrane. It is tempting to speculate that a relative abundance of TBDT transporters in *Shewanella *and some other environmental Proteobacteria reflects their rather limited access to carbohydrates as compared to Enterobacteria.

#### Transcriptional regulation

is another highly variable aspect of the sugar utilization machinery. Indeed, 11 of the 17 transcriptional regulators tentatively associated with *Shewanella *sugar utilization pathways are nonorthologous to their counterparts previously characterized in *E. coli *and other species (Table 2). Moreover, 9 of these transcription factors were recruited from structurally unrelated protein families, and they are predicted to bind to completely distinct DNA motifs. For example, the transcriptional repressor NagR of the Nag utilization pathway in *Shewanella *belongs to the LacI family, whereas a functionally equivalent NagC regulator of *E. coli *belongs to the ROK family. Therefore, a functional repertoire of co-regulated genes appears to be generally better conserved between species than the respective transcription factors and their DNA binding motifs [31]. Members of the LacI family are most abundant among the regulators of sugar catabolism in *Shewanella*. They control 10 of the 17 reconstructed pathways, whereas the remaining pathways are regulated by the members of GntR and DeoR families (Fig. 3). The majority of genes from sugar catabolic pathways were identified as candidate members of respective sugar catabolic regulons in *Shewanella *genomes (see additional data file 3).

#### Sugar catabolic enzymes

are far less variable between species than associated transporters or regulators. In case of *Shewanella*, the most notable variations are associated with sugar phosphorylation, which is at least partially due to a functional replacement of the uptake-coupled phosphorylation (characterstic of PTS) by a combination of permease and kinase or, less frequently, phopshorylase (for disaccharides). Thus, in the aforementioned Nag and Aga pathways, novel sugar kinases NagK and AgaK combined with respective permeases (NagP and AgaP) are employed in *Shewanella *spp rather than canonical PTS systems. In a novel variant of Scr utilization pathway predicted and experimentally validated in *S. frigidimarina *(see additional data file 6), the hydrolysis of sucrose upon ScrT-mediated uptake and phosphorylation of its glucose moiety is performed by the sucrose phosphorylase (ScrP). This single-enzyme transformation is a functional replacement of two consecutive enzymatic reactions, phosphotransferase by PTS (ScrA) followed by sucrose-6-phosphate hydrolase (ScrB), in a canonical version of the pathway described in Enterobacteria [24]. Nonorthologous replacements appear to be quite common for sugar kinases that are known to occur in a number of distinct protein families. For example, the glucokinase Glk^II ^of *Shewanella *belongs to the ROK family, whereas its functional counterpart in *E. coli *and other Enterobacteria belongs to the bacterial glucokinase family.

## Discussion

Most heterotrophic bacteria are capable of utilizing at least some carbohydrates as a source of carbon and energy via a matching repertoire of sugar catabolic pathways. Such pathways that include specialized transporters and intracellular enzymes catalyzing biochemical transformations of a particular sugar into one of the common CCM intermediates often form operons controlled by committed transcription factors. Despite our advanced understanding of sugar catabolic pathways in a few model bacteria, their genomics-based projection across a variety of species from other taxonomic groups is quite challenging due to the intrinsic variability of the carbohydrate utilization machinery. To address this challenge we have established a subsystems-based comparative genomic approach (Fig. 1), and assessed its utility by building a genomic encyclopedia of carbohydrate catabolism (*sugar catabolome*) in the *Shewanella *genus. This analysis covered 19 complete genomes of diverse *Shewanella *species isolated from various aquatic habitats. The key components of our approach include: (i) using a collection of protein families capturing the current knowledge of the microbial carbohydrate utilization machinery for homology scanning of target genomes and preliminary identification of gene candidates; (ii) analyzing genomic and functional contexts to recognize functional equivalents of previously characterized genes and to predict novel genes and pathway variants; (iii) validating selected bioinformatic predictions by a combination of biochemical, genetic and physiological experiments.

One of the critical factors that contributed to the successful outcome of this analysis was the availability of a large set of completely sequenced genomes (largely due to the DOE Joint Genome Institute, http://www.jgi.doe.gov/) representing a substantial phylogenetic, geographic and ecophysiological diversity within an otherwise compact taxonomic group -- *Shewanella *genus [1]. This was particularly instrumental for mapping and prediction of regulons as well as for reliably establishing orthology relationships within protein families containing multiple paralogs. In addition to improving our knowledge of genomics, functional organization and evolution of the sugar catabolome, this study confirmed the efficiency of the established approach, which is scalable and applicable to other groups of microorganisms. A systematic application of this approach to a growing number of divergent bacterial lineages would allow us to rapidly expand the current genomic knowledgebase and establish the capability of highly accurate automated annotation and assertion of sugar catabolic pathways in all bacteria with completely sequenced genomes. Moreover, a fine-grain functional annotation of all protein families (including those with multiple branches and paralogs) that comprise carbohydrate utilization machinery in complete genomes will enable accurate recognition of the corresponding functions and pathway variants in metagenomic samples.

An overall internal consistency of the reconstructed catabolic machinery confirmed the efficiency of the bioinformatic workflow used in this study. The current version of the genomic encyclopedia of carbohydrate utilization pathways of the *Shewanella *genus has neither obvious gaps such as "missing genes" (required by functional context but not identified in the genomes), nor inconsistencies such as "disconnected genes" (those that were functionally assigned but lack any functional context). The only notable exception is an already mentioned putative PTS system whose function remains obscure despite its high similarity with *E. coli *glucose PTS. Additional support of the developed genomic reconstruction was provided by direct experimental testing of selected bioinformatic predictions using both *in vitro *assays of purified recombinant enzymes and *in vivo *phenotype assessment of gene knockouts in *Shewanella *and genetic complementation in *E. coli*. Despite the overall successful outcome and utility of these experiments, the scale of this reconstruction makes a traditional targeted verification of individual conjectures rather impractical. On the other hand, a predicted pattern of presence/absence of certain sugar utilization pathways within a representative group of species may be seamlessly translated to a predicted testable phenotype, the ability/inability of these species to metabolize respective sugars.

We assessed the panel of 14 *Shewanella *species for the aerobic growth on a series of 18 sugars used as a single source of carbon and energy (see additional data files 7 and 8). The results of the extended growth profiling in this study revealed a remarkable consistency between predicted and observed phenotypes for the entire matrix of tested strains and sugars despite some strain-to-strain variations in the growth rate and efficiency (Table 3). In addition, the absence of the D-xylose and D-fructose catabolic pathways in the entire *Shewanella *group was also confirmed by their inability to grow on either of these sugars as a carbon and energy source. The only two observed inconsistencies, the inability of *S. oneidensis *to grow on maltodextrin and of *S. frigidimarina *to grow on mannitol, can be rationalized by genetic rearrangements. Among the genes in the *mal *locus of *S. oneidensis *disrupted by insertion elements and frameshifts is an already mentioned transporter GlcP^Mal^. This observation also provided an explanation of the intrinsic inability of *S. oneidensis *to grow on Glc, in contrast to many other *Shewanella *spp, including ANA-3, where the role of this transporter in Glc utilization was experimentally confirmed. It is worth mentioning that our phenotype predictions are largely consistent with the results of earlier growth studies published for a limited set of strains and sugar substrates [13,15,32-35].

Analysis of the distribution of sugar utilization pathways within the *Shewanella *genus and a comparison with *E. coli *and other bacteria with different life-styles and natural environments yields interesting observations and conjectures about their adaptive evolution (Fig. 4). A clear distinction between the *core *and the *rare *pathways reflects an apparent difference in their evolutionary history. As mentioned above, the former group probably existed in a common ancestor of *Shewanella *spp. Moreover, some of these pathways appear to remain essential in most of the diverse *Shewanella *habitats as they were lost in only a few rare cases. Thus, the Nag pathway is preserved in 18 and the Grt pathway in 17 of the 19 compared species. This is reminiscent of the distribution of a recently described lactate utilization machinery [8], which is present in all but one species consistent with its proposed role of the main natural supplier of carbon and energy characteristic of the entire *Shewanella *genus. On the other hand, rare pathways (present in 1-4 species) have more likely originated via lateral gene transfer events, possibly from species sharing the same ecological niche. This may be illustrated by the example of the Gnt utilization pathway that is present in all 4 sequenced strains of *S. baltica *but not in any other *Shewanella *spp. A conservation of sequence and genomic arrangement within the Gnt and Rbs gene loci between some closely related *Shewanella *strains and distant members of Enterobacteriaceae, Pasteurellaceae and Vibrionaceae points to their likely acquisition via a relatively recent lateral gene transfer event (see additional data file 10).

## Conclusions

Overall, the results of this study revealed elements of conservation and variation that appear to be characteristic of the sugar utilization machinery. All reconstructed pathways show a strong tendency to be encoded within compact operons and regulons, and nearly all of the newly identified individual genes are at least distantly homologous (and functionally related) to those previously characterized in other species and included in our original collection of sugar utilization subsystems.

The reconstructed sugar catabolome in *Shewanella *spp utilizes 170 distinct proteins forming 17 peripheral sugar catabolic pathways and CCM (Table 2, Fig. 1). An apparent "core" subset of peripheral pathways for the utilization of Nag, Grt, Mal and Glc is conserved in >70% of compared *Shewanella *genomes. This level of conservation not only points to their ancestral origin but to an apparent importance of these pathways (and respective sugars) for the physiology of *Shewanella *spp in various ecosystems. All other peripheral pathways are present in <50% of the compared genomes, and among them, 9 pathways are present only in 1-4 species. The latter group seemingly originates from the lateral gene transfer and likely reflects adaptation to specific environmental conditions, which is particularly notable in *S. frigidimarina *and *S. pealeana*, each containing nonoverlapping sets of 3 "rare" pathways (Fig. 4). Even the most versatile *Shewanella *species implement only a fraction of the entire pan-*Shewanella *sugar catabolome reflective of their relatively "lean sugar diet".

## Methods

### The subsystems approach and the SEED platform

Our approach to the reconstruction of sugar catabolic pathways in a selected group of genomes was based on functional gene annotation and prediction using two principal comparative genomics techniques: (i) homology-based methods and (ii) genome context analysis. Both these methods are implemented in the SEED genomic platform http://theseed.uchicago.edu/FIG/ that combines a large and rapidly growing integration of >700 complete annotated genomes (mostly bacterial) with advanced tools for comparative analysis, gene annotation, genome context analysis and functional reconstruction based on subsystems technology [7]. New genomes are automatically annotated by the RAST server http://rast.nmpdr.org/, a new generation of subsystem-based genome annotation tools [36]. Subsystems in the SEED provide the framework for further improvement of these annotations and functional predictions. They are sets of functional roles that capture the current knowledge of cellular processes and metabolic pathways including interspecific variation. Each functional role is typically associated with a set of homologous genes that implement this role in specific organisms. In addition to homology-based analysis suggesting at least general class gene functional assignments, genome context analysis provides evidence of functional coupling between genes of known and unknown functions [10]. The most common type of functional coupling evidence comes from the tendency of functionally related genes (e.g., members of the same pathway) to be clustered on the chromosome. Other important types of evidence are domain fusion events, conservation of upstream regulatory sites (i.e., reg-ulons) and co-occurrence profiles of genes across a range of genomes. We used the tools in SEED and other public servers to compute and analyze all types of functional coupling evidence for each gene family in our analysis.

### Reconstruction of regulons

For identification of a candidate regulatory motif for a particular sugar catabolic pathway we started from a training set of potentially co-regulated genes participating in the pathway. Upstream regions of genes from the training set and their orthologs from multiple *Shewanella *genomes were used as an input for a DNA motif detection algorithm. A simple iterative procedure implemented in the program SignalX (as described previously in [31]) was used for construction of a common transcription factor-binding motif in sets of upstream gene fragments. Each genome encoding the studied transcription factor was scanned with the constructed profile using the GenomeExplorer software [37], and genes with candidate regulatory sites in the upstream regions were selected [38]. The threshold for the site search was defined as the lowest score observed in the training set. Among new candidate members of a regulon, only genes having candidate sites conserved in at least two other genomes were retained for further analysis. We also included new candidate regulon members that are functionally related to the reconstructed sugar catabolic pathways. Sequence logos for derived regulatory motifs were drawn using the WebLogo package http://weblogo.berkeley.edu[39]. The details of reconstructed regulons are captured and displayed in the specialized database RegPrecise http://regprecise.lbl.gov[40].

### Workflow

Reconstruction of metabolic and regulatory pathways involved in the carbohydrates utilization was performed for 19 species of the *Shewanella *genus with completely sequenced genomes uploaded from Genbank and integrated in the SEED genomic platform. The overall workflow is illustrated by Fig. 1. First we performed a survey of all prokaryotic genes known to be involved or potentially involved in utilization of mono- and di-saccharides. A collection of ~480 FIGfams from 35 SEED sugar metabolic subsystems were classified by their general functional role (i.e. sugar transport, transcriptional regulation, biochemical transformation, and upstream/auxiliary) (see additional data file 1). Each FIGfam comprises a functionally uniform group of orthologous proteins in related organisms. This extensive collection was then used for homology searches against 19 *Shewanella *genomes resulting in identification of numerous FIGfams potentially implicated in sugar metabolism. Manual curation of the identified *Shewanella *FIGfams using the SEED and other genomic resources and tools (see below) rejected many of them as well as identified some additional candidate FIGfams based on genome context analysis. As a result of this iterative process we have identified ~170 FIGfams present in at least one *Shewanella *genome and tentatively assigned a role in utilization of a paticular sugar substrate (see additional data file 2). The identified FIGfams were used for metabolic reconstruction of *Shewanella *sugar catabolic pathways using the subsystems-based approach. The refined functional annotations were combined in the aggregated SEED subsystem 'Sugar catabolome in *Shewanella *species'. Besides SEED, we routinely use other bioinformatic tools and databases featuring: genomes (Genbank), gene annotations (UniProt, IMG), primary literature (PubMed), reactions and pathways (KEGG, BioCyc), conserved domains and motifs (COG, PFAM, ProDom), distant homology searches and alignments (PsiBlast, FFAS, T-Coffee), genome context and occurence profiles (STRING, Microbes on Line), transcriptional regulation (RegTransBase, RegulonDB), protein localization prediction (TMPRED, SignalP).

### Experimental validation of functional predictions

#### Bacterial strains, plasmids, and reagents

*E. coli *strains DH5α (Invitrogen, Carlsbad, CA) and the knockout mutant Δ*bglF *from the KEIO collection (a kind gift from Dr. Mori) [41], were used for complementation analyses. *E. coli *BL21/DE3 (Gibco-BRL, Rockville, MD) was used for protein overexpression and purification. For expression of genes in *E. coli*, a pBAD-TOPO vector containing the arabinose promoter (Invitrogen, Carlsbad, CA) or a pET-derived vector containing the T7 promoter, His_6 _tag, and tobacco etch virus-protease cleavage site were used. Enzymes for PCR and DNA manipulations were from New England Biolabs Inc. (Beverly, MA). Plasmid purification kits were from Promega (Madison, WI). PCR purification kits and nickel-nitrilotriacetic acid (Ni-NTA) resin were from QIAGEN Inc. (Valencia, CA). Oligonucleotides for PCR and sequencing were synthesized by Sigma-Genosys (Woodlands, TX). All other chemicals, including sucrose, cellobiose, NADH, ATP, phosphenolpyruvate, lactate dehydrogenase, pyruvate kinase, D-glucose, 2-deoxy-D-glucose, D-mannose, D-galactose, D-allose, D-fructose, L-sorbose, D-tagatose, D-glucosamine, D-mannosamine, D-galactosamine, *N-*acetylglucosamine, *N-*acetylmannosamine, *N-*acetylgalactosamine were purchased from Sigma-Aldrich (St. Louis, MO).

#### PCR amplification and cloning

The 2.9-kb fragment from *Shewanella frigidimarina *NCIMB400, which contains two divergently transcribed genes, *scrT^II ^*and *scrP *(Sfri_3989 and Sfri_3990, respectively) and the intergenic region, was amplified using the primers: 5'-gcgTTATTTAGCGTCTGCGGTCAACAAATG (forward-1) and 5'-gagTTATCCGCTGTGTTTAGCCAGTAAATC (reverse-1). For cloning the fragment containing *scrT^II ^*only and the intergenic region, the primers of forward-1 and 5'-CGCTATACGATCTACGTAAGTGATCAGCTG were used. For cloning the fragment containing *scrP *only and the intergenic region, the primers of 5'-CTCATTATCCTTCATATGGTTAACCATAC and reverse-1 were used.

The 2.7-kb fragment from *Shewanella baltica *OS155, which contains the *bglA*^*I*^and *bglT *genes (Sbal_0544 and Sbal_0545, repectively), was amplified using the primers: 5'-gaggaataataaATGAAAATATCTTTACCAAAG (forward-2) and 5'-cacTTAGCTGGCTCTATTTAATTCCAG (reverse-2). For cloning Sbal_0544 only, the primers of forward-2 and 5'-gccTTATTTATTATTGTTATTAGTAAAGTGAGC were used. For cloning Sbal_0545 only, the primers of 5'-gaggaataataaATGATTAGCATAAAAGAAAAAATAG and reverse-2 were used. Nucleotides not present in the original sequence are shown in lowercase. PCR amplification was performed using the genomic DNAs of *S. frigidimarina *NCIMB400 and *S. baltica *OS155. The PCR fragments as describe above were cloned into the pBAD-TOPO expression vector.

The *glk*^II ^gene from *S. baltica *OS155 (*Sbal_1134*) was amplified using the primers: 5'-ggcgc**acATGT**TACGAATTGGTATCGATCTTG (forward-3) and 5'-gcaac**gtcgac**TTAGCGTCCCCACAACCAAGC (reverse-3). Introduced restriction sites (*Pci*I for forward-3 primer and *Sal*I for reverse-3 primer) are shown in boldface. PCR fragment was cloned into the pET-derived expression vector cleaved by *Nco*I and *Sal*I. Selected clones were confirmed by DNA sequence analysis.

#### Protein purification

Recombinant protein of *glk*^II ^(*Sbal_1134*) from *S. baltica *OS155 was overexpressed as N-terminal fusion with a His_6 _tag in *E. coli *strain BL21/DE3. Cells were grown on LB media to OD_600 _= 0.8 at 37°C, induced by 0.2 mM IPTG, and harvested after 12 h shaking at 20°C. Protein purification was performed using rapid Ni-NTA agarose minicolumn. Briefly, harvested cells were resuspended in 20 mM HEPES buffer pH 7 containing 100 mM NaCl, 0.03% Brij 35, and 2 mM β-mercaptoethanol supplemented with 2 mM phenylmethylsulfonyl fluoride and a protease inhibitor cocktail (Sigma-Aldrich). Lysozyme was added to 1 mg/mL, and the cells were lyzed by freezing-thawing followed by sonication. After centrifugation at 18,000 rpm, the Tris-HCl buffer (pH 8) was added to the supernatant (50 mM, final concentration), and it was loaded onto a Ni-NTA agarose column (0.2 ml). After washing with the starting buffer containing 1 M NaCl and 0.3% Brij-35, bound proteins were eluted with 0.3 ml of the starting buffer containing 250 mM imidazole. Protein size, expression level, distribution between soluble and insoluble forms, and extent of purification were monitored by SDS-PAGE.

#### Complementation analyses

In all complementation analyses, cells were pre-cultured on LB media to exponential growth phase, harvested by centrifugation, and washed for three times with M9 minimal media without any carbon sources. All cultures were started with the same optical density at 600 nm (OD_600 nm _= 0.03), and performed at 37°C in triplicates in 200 μl of the respective media. The cell growth was monitored spectrophotometrically at 600 nm using a microplate reader (ELx808, BioTek Inc., Winnoski, Vermont).

Recombinant proteins of *S. frigidimarina *NCIMB400 ScrT^II ^(*Sfri_3989*) only, ScrP (*Sfri_3990*) only, and ScrT^II^-ScrP were expressed in *E. coli *K-12 strain DH5α under the control of endogenous promoter in the intergenic region. The empty pBAD-TOPO vector was expressed in the same strain and used as a negative control. The complementation analysis was performed on M9 minimal media supplemented with 50 μg/ml of thiamine and 20 mM of sucrose.

Recombinant proteins of *S. baltica *OS155 BglA^I ^(*Sbal_0544*) only, BglT (*Sbal_0545*) only, and BglA^I^-BglT were expressed under the control of arabinose promoter in *E. coli *Δ*bglF *mutant. The empty pBAD-TOPO vector was expressed in the same strain and used as a negative control. The complementation analysis was performed on M9 minimal media supplemented with 0.15% of L-arabinose and 20 mM of cellobiose.

#### In vitro enzyme assays

Glucokinase activity was assayed by coupling the formation of ADP to the oxidation of NADH to NAD^+ ^via pyruvate kinase and lactate dehydrogenase and monitored at 340 nm. Briefly, 0.1-0.2 μg of purified glucokinase was added to 200 μL of reaction mixture containing 50 mM Tris buffer (pH 7.5), 10 mM MgSO_4_, 1.2 mM ATP, 1.2 mM phosphoenolpyruvate, 0.3 mM NADH, 1.2 U of pyruvate kinase, 1.2 U of lactate dehydrogenase, and 5 mM D-glucose at 37°C. No activity was detected in a control experiment, in which an unrelated gene (SO3505) was expressed in the same vector and purified in parallel. The substrate specificities were examined by using the same assay method and exchanging glucose for 5 mM of other potential substrates: 2-deoxyglucose, D-mannose, D-galactose, D-allose, D-fructose, L-sorbose, *N-*acetyl-D-mannosamine, *N-*acetyl-D-glucosamine, *N-*acetyl-D-galactosamine, D-galactosamine, D-glucosamine, D-mannosamine. In the coupled assays, the change in NADH absorbance was monitored at 340 nm using a Beckman DTX-880 multimode microplate reader. An NADH extinction coefficient of 6.22 mM^-1^cm^-1 ^was used for rate calculation.

#### Phenotypic analysis of Shewanella spp

Total 14 *Shewanella *strains were tested for their ability to grow on 20 different carbon sources as a sole carbon and energy source. D-/L-lactate mixture was used as a control. Growth conditions and other details of two different experimental techniques used for growth phenotype analysis are provided in additional files 7 and 8.

#### In-frame deletion mutagenesis

In-frame deletion mutagenesis of *glyT *(SO1771) or *nagP *(SO3503) was performed using previously published method [42] with minor modifications. Upstream and downstream fragments flanking the target locus were PCR amplified using *S. oneidensis *MR-1 genomic DNA and fused via overlap extension PCR. The fusion PCR amplicon was ligated into XcmI-digested pDS3.0. The resulting recombinant plasmids were used to transform *E. coli *ß-2155 or WM3063 and subsequently transferred to *S. oneidensis *strain MR-1 by conjugation. The primary integrants were selected by plating on LB medium containing 7.5 μg/ml gentamycin. The screening for a second round of homologous recombination to remove the plasmid sequence was accomplished by counter-selection on LB medium with 5% sucrose. Colonies growing in the presence of sucrose were screened for sensitivity to gentamycin (7.5 μg/ml) and then screened for deletion of the gene of interest using PCR. The resulting PCR amplicon was then used as the template for DNA sequencing of the deleted and flanking regions involved in the recombination events (ACGT, Inc. Wheeling, IL). In order to validate that the observed phenotype could be attributed to the targeted deletion, complementing plasmids were constructed by inserting appropriate genes downstream to the lac promoter encoded in pBBR1MCS-5.

## List of abbreviations

PTS: phosphotransferase system; FIGFAMS: isofunctional protein families in the SEED database; CCN: central carbon metabolism; ED pathway: Entner-Doudoroff pathway; PP pathway: pentose phosphate pathway; TBDT: TonB-dependent outer membrane transporter; Aga: *N-*acetylgalactosamine; Ara: L-arabinose; Bgl: β-glucoside (cellobiose); Gal: D-galactose; Glc: D-glucose; Gnt: D-gluconate; Grt: D-glycerate; Mal: maltodextrin; Man: mannosides; Mtl: mannitol; Nag: *N-*acetylglucosamine; Nan: sialic acids; Rbs: D-ribose; Scr: sucrose; Tre: trehalose; Xlt: xylitol.

## Authors' contributions

DAR, CY, and AO conceived and supervised the research, and wrote the manuscript. DAR performed comparative genomic analysis to infer novel metabolic pathways and regulons. RO and OPZ performed computational similarity searches, gene annotation and subsystem encoding in the SEED database. CY, XL, and IAR carried out genetic complementation experiments. MFR and SR produced targeted gene knockout strains in *Shewanella*. YW, AYO, and SR performed phenotypic characterization of *Shewanella *strains. JKF and KHN contributed to the development of the manuscript and design of the study. All authors read and approved the final manuscript.

## Supplementary Material

Additional file 1 **Collection of protein families involved in carbohydrate utilization in bacteria**. Click here for file

Additional file 2 **Summary on distribution of carbohydrate utlization genes in *Shewanella *spp**. Click here for file

Additional file 3 **Complete distribution of sugar catabolome genes in *Shewanella *genomes**. Click here for file

Additional file 4 **Description of *rare *sugar utilization pathways in *Shewanella***. Click here for file

Additional file 5 **Predicted novel functional roles for isofunctional protein families from *Shewanella *sugar catabolome**. Click here for file

Additional file 6 **Experimental verification of novel enzymes, transporters, and regulators involved in sugar utilization in *Shewanella***. A. Phenotypic characterization of *glcP*^Mal ^(*Shewana3_2310*) for its involvement in glucose utilization in *Shewanella *sp. ANA-3; B. Phenotypic characterization of *nagP *(*SO3503*) for its involvement in *N-*acetylglucosamine (Nag) utilization in *Shewanella oneidensis *MR-1; C. Phenotypic characterization of *grtP *(*SO1771*) for its involvement in D-glycerate; D. Complementation of the *E. coli *cellobiose utilization by the *bglA-bglT *(*Sbal_1133-1132*) genes from *S. baltica *OS155; E. Substrate specificity of *Shewanella baltica *OS155 Glk^II ^(Sbal_1134) kinase; F. Growth of *E. coli *DH5a strain (Scr-) containing heterologously expressed sucrose utilization genes *scrTII-scrP *(*Sfri_3989-3990*) from *S. frigidimarina*. Click here for file

Additional file 7 **Growth phenotypes of *Shewanella *on various carbon sources determined by manual assay**. Click here for file

Additional file 8 **Growth phenotypes of *Shewanella *on various carbon sources determined by microplate assay using Bioscreen C MBR system**. Click here for file

Additional file 9 **Comparison of reconstructed sugar utilization pathways in *Shewanella *and Enterobacteria**. A. *N-*acetylglucosamine (Nag) and chitin utilization pathways; B. D-glycerate (Grt) and glucarate/galactarate utilization pathways; C. β-glucoside (Bgl) utilization pathway; D. Sucrose (Scr) utilization pathway; E. L-arabinose (Ara) and arabinosides utilization pathways. Click here for file

Additional file 10 **The gluconate (A) and ribose (B) utilization gene loci in some closely related *Shewanella *strains and other γ-proteobacteria**. Click here for file
