# Supplementary material for: Genomic encyclopedia of sugar utilization pathways in the Shewanella genus
Source: BMC Genomics. 2010 Sep 13;11:494. doi: 10.1186/1471-2164-11-494 (PMC2996990; doi:10.1186/1471-2164-11-494)
Supplement: Additional file 4 — Description of rare sugar utilization pathways in Shewanella. [file 1471-2164-11-494-S4.DOC]

**Additional file 4 - Description of “rare” sugar utilization pathways in *Shewanella*.**

***Gluconate*** (Gnt) utilization gene locus, encoding the gluconate transporter GntU, gluconokinase GntK, and the LacI-type transcriptional regulator GntR, was identified only in four *S. baltica* strains (Fig. 3B, see additional data file 2). The *gntU, gntK* and *gntR* genes in *S. baltica* are orthologs of the previously characterized Gnt utilization genes from *E. coli* [1]. Tandem GntR-binding sites identified in the common upstream region of the *gntK* and *gntU* genes in *S. baltica* resemble the consensus binding motif of the gluconate repressor GntR from *E. coli* (Fig. 3B).

The growth phenotype analysis of 10 *Shewanella* species is fully consistent with the genomic reconstruction: only *S. baltica* was able to grow on D-gluconate as a sole carbon and energy source (see additional data file 7 and Table 3).

The phylogenetic analysis suggests that the Gnt catabolic gene cluster was likely acquired by a common ancestor of the analyzed *S. baltica* strains from other γ-proteobacteria. The closest homologs of *gnt* genes from *S. baltica* were identified in the genomes of bacteria from the orders Pasteurellales and Enterobacteriales (see additional data file 10). Ecophysiological consequence of likely acquisition of the Gnt utilization genes by *S. baltica* strains (isolated from sea water in the Baltic sea) is yet to be elucidated.

***N-acetylgalactosamine*** (Aga) utilization gene cluster identified in four *Shewanella* species (*S. amazonensis*, the species MR-4, MR-7 and ANA-3) includes an ortholog of the previously characterized *E. coli* DeoR-type transcriptional regulator AgaR [2]. Candidate AgaR-binding sites were identified upstream of the divergently transcribed *agaROZSKA^II^P* and *omp^Aga^* operons (Fig. 3B; see also additional data file 3). The reconstructed Aga catabolic pathway in *Shewanella* involves a single *E. coli*-like enzyme, tagatose-6-phosphate kinase AgaZ, and five novel functional roles, namely AgaP, Omp^Aga^, AgaK, AgaA^II^, and AgaS (Fig. 2, see also additional data file 2). The predicted *N-*acetylgalactosamine permease AgaP belongs to the GGP sugar transporter family and is a close paralog of the *N-*acetylglucosamine permease NagP in *Shewanella* (50% identity). Omp^Aga^ is the predicted outer membrane Aga transporter from the TBDT family. The predicted Aga kinase AgaK is a novel ROK-family kinase homologous to the *Shewanella* glucokinase Glk^II^ (35% similarity). AgaA^II^ is a close paralog (50% similarity) of the Nag-6-phosphate deacetylase NagA from *Shewanella*. The predicted galactosamine-6P isomerase AgaS belongs to the phosphosugar isomerase protein family, and is similar to the *Shewanella* glucosamine-6-phosphate deaminase NagB^II^. The functional role of the *agaO* gene encoding an outer membrane lipoprotein oxidoreductase belonging to the Gfo/Idh/MocA family in the Aga pathway is unknown.

The growth phenotype characterization of 10 *Shewanella* species demonstrated that only two of them are able to grow on *N-*acetylgalactosamine as a sole carbon and energy source (see additional data file 7). These results are consistent with the distribution of the Aga utilization genes in the analyzed *Shewanella* genomes (Table 3).

Phylogenetic analysis suggests that several components of the novel Aga catabolic pathway (AgaP, AgaK, AgaA) are present only in the *Shewanella* genus and were likely emerged via gene duplication followed by their functional divergence. In contrast, closest orthologs of other components (AgaR, AgaS and AgaZ) were identified in the Enterobacteriales and Vibrionales groups. This reconstruction suggests that the evolutionary scenario for the Aga catabolic pathway in *Shewanella* includes both emergence of new genes via gene duplication and gene acquisition via lateral gene transfer (LGT). Ecophysiological importance of the Aga utilization pathway for these four *Shewanella* species that were isolated from various aquatic sources, such as the Black sea or Amazon river delta, is not clear. One possibility is that they colonize aquatic animals and utilize Aga from the host intestinal mucin.

***Trehalose*** (Tre) utilization genes were identified in three *Shewanella* species. The conventional Tre catabolic pathway in *E. coli* uses a trehalose-specific PTS transporter and a trehalose-6-phosphate hydrolase [3]. The reconstructed Tre utilization pathway in *S. frigidimarina* involves two novel trehalose transporters (TreT and Omp^Tre^) and cytoplasmic trehalase enzyme TreF (which catalyzes trehalose hydrolysis into two molecules of β-D-glucose (Table 2, Fig. 2)). The predicted trehalose transporter TreT in *Shewanella* belongs to the GGP family of sugar transporters and is most similar to the predicted *Shewanella* sucrose transporter ScrT^II^ (27% identity). The predicted TBDT Omp^Tre^ is presumably involved in the uptake of trehalose into the periplasm. Two other *Shewanella* spp, *S. woodyi* and *S. baltica* OS223, have TreT and Omp^Tre^ orthologs accompanied by the predicted trehalose phosphorylase TreP and a paralog of β-phosphoglucomutase Pgm. These constitute an alternative pathway of Tre utilization (Fig. 2; see also additional data file 2). TreP is similar to kojibiose phosphorylase from *Thermoanaerobacter brockii* (Swiss-Prot accession Q8L163), which hydrolyzes this rare disaccharide in the presence of inorganic phosphate to form D-glucose and D-glucose-1-phosphate, which is further converted to glucose-6-phosphate by Pgm.

The Tre catabolic gene loci in *Shewanella* contain a novel LacI-type transcriptional regulator TreR^II^, which is a nonorthologous replacement of the previously characterized TreR repressors from other bacteria. A comparative genomic reconstruction of the TreR^II^ regulon allowed us to predict its candidate binding sites located upstream of the divergently transcribed *omp^Tre^* and *treR^II^* genes in all three *Shewanella*, as well as upstream of the *treT-treF* operon in *S. frigidimarina* and the *treT<>treP-pgm* divergon in *S. woodyi* and *S. baltica* OS223 (Fig. 3B; see also additional data file 3).

The results of growth phenotype profiling of 14 *Shewanella* species on trehalose are consistent with the genomic reconstruction of the Tre catabolic pathway, which appears to be present only in *S. frigidimarina* and *S. baltica* OS223 (Table 3).

Phylogenetic analysis of the *treT* genes suggests that, though these genes have multiple orthologs within the Alteromonadales lineage, the *treT* genes from *Shewanella* are not monophyletic, suggesting their independent acquisition by LGT. The *S. frigidimarina* *treT* gene is most similar to an ortholog from *Pseudoalteromonas atlantica*, whereas the respective genes from *S. baltica* and *S. woodyi* are most similar to an ortholog from *Colwellia psychrerythraea*. The observed splitting of the *treT* genes in the phylogenetic tree is in agreement with the observed differences in their genomic context and, consequently, in the respective variants of the Tre catabolic pathway (utilizing either TreP or TreF). From ecophysiological perspective, many saline-water organisms synthesize trehalose for osmoprotection, providing a possible source of this disaccharide for its subsequent degradation via the Tre catabolic pathways in *Shewanella* and other marine bacteria.

Two distinct ***mannoside*** (Man) utilization gene loci were tentatively identified in two *Shewanella* species, *S. amazonensis* and *Shewanella* sp. MR-7 (termed *man-I* and *man-II*). The known mannose utilization pathway in *E. coli* includes a mannose-specific PTS transporter ManXYZ and a mannose-6-phosphate isomerase ManA. Among the analyzed *Shewanella* genomes, only two *S. putrefaciens* strains, CN-32 and W3-18-1, possess orthologs of the *manXYZ* operon, though the first gene in this operon is interrupted by a transposase, suggesting that this PTS^Man^ system is not functional. Moreover, all analyzed *Shewanella* genomes lack a *manA* ortholog.

The *man-I* locus of *S. amazonensis* and *Shewanella* sp. MR-7 contains the candidate *mnnA1-mnnA2-manP^I^-manK-manI* operon and the divergently located *manR^I^* gene (Fig. 3B, see also additional data file 2). The *mnnA* genes encode proteins from the α-1,2-mannosidase family with candidate signal peptides suggesting their extracytoplasmic localization. The functional roles of the hypothetical genes *manP^I^, manK, manI* and *manR^I^* were predicted based on tentative reconstruction of the mannoside utilization pathway (Table 2, Fig. 2; see additional data file 5). A predicted mannose permease ManP from the GGP family is a close paralog of the predicted glucose permeases, GlcP^Bgl^ and GlcP^Mal^ (55% identity). A newly identified mannose isomerase ManI belongs to the *N-*acylglucosamine 2-epimerase family, and it is similar (30% identity) to a recently characterized mannose isomerase YihS from *E. coli* [4]. A predicted fructokinase ManK is similar (37% identity) to the fructokinase ScrK from *Shewanella* species. The mannoside utilization pathway reconstructed in *Shewanella* involves hydrolysis of mannose oligosaccharides in the periplasm, permease-mediated uptake of mannose, its intracellular conversion to fructose, and final phosphorylation to produce fructose-6-phosphate (Fig. 2). The *man-I* genetic locus in *Shewanella* was predicted to be under transcriptional control of a novel LacI-type regulator ManR^I^ with unique DNA-binding sites (Fig. 3B).

In addition to *man-I*, *S. amazonensis* has a second mannoside catabolic locus (named *man-II*), which involves multiple mannosidase genes, paralogs of the *manP^I^-manK-manI* genes, a different LacI-type regulatory gene *manR^II^*, and a candidate mannoside-specific TBDT gene *omp*^Man^ (Fig. 3B). ManR^II^ presumably controls most operons within the *man-II* gene locus by binding to its candidate binding sites that have a consensus motif different from that of ManR^I^.

Phylogenetic analysis of the Man catabolic genes suggests that this novel Man pathway variant is restricted to the Alteromonadales lineage, since orthologs of the *man* genes were only identified in *Pseudoalteromonas atlantica*, and *Colwellia psychrerythraea*. These bioinformatic predictions remain to be tested experimentally.

***Xylitol*** (Xlt) utilization gene cluster, a *xltR<>xylDB-xltABC* divergon, is novel system found only in *S. pealeana* and *S. halifaxensis* (see additional data file 2). It encodes orthologs of xylitol dehydrogenase XylD and xylulokinase XylB from Enterobacteria [5], as well as novel ABC-type xylitol transporter XltABC and LacI-type transcriptional regulator XltR (Fig. 2). The predicted xylitol transporter XltABC in *Shewanella* is similar to the ribose transporter RbsABC from *E. coli* (32% identity). Comparative genomic reconstruction of a novel XltR regulon allowed us to predict its candidate binding sites located in tandem within common regulatory region of *xylD* and *xylR* in both *Shewanella* genomes (Fig. 3B). No experimental work was performed in this study with any of the two species, *S. pealeana* and *S. halifaxensis,* containing the xylitol utilization pathway.

Phylogenetic analysis of the *xlt* genes from two closely related *Shewanella* spp. suggests their likely acquisition via LGT from the Enterobacteriales lineage. From ecophysiological perspective, the acquisition of Xlt pathway can be advantageous for animal-associated microorganisms, such as *S. pealeana* (isolated from squid), since Xlt is known to be contained in animal tissues [6].

***Ribose*** (Rbs) utilization gene cluster was found in two *Shewanella* genomes, *S. pealeana* and *S. halifaxensis* (see additional data file 2). The *rbsDACBKR* operon is similar to the ribose catabolic operon from *E. coli* and encodes the ABC-type ribose transport system RbsABCD, ribokinase RbsK and the LacI-type transcriptional regulator RbsR (Fig. 2). Candidate RbsR-binding sites identified upstream of the *rbs* operons in two *Shewanella* spp resemble the consensus sequence of RbsR from *E. coli* (Fig. 3B). No experimental work was performed in this study with any of the two species, *S. pealeana* and *S. halifaxensis,* containing the ribose utilization pathway, though the previously published experimental data confirm their ability to grown on ribose [7, 8].

Phylogenetic analysis of the Rbs utilization genes suggests that the *rbs* gene cluster was acquired by a common ancestor of *S. pealeana* and *S. halifaxensis* via LGT from the Vibrionales lineage. *Vibrio* spp. inhabit animal-associated ecological niches in seas and oceans and are known as pathogens or commensals in the microflora of marine animals. From ecophysiological perspective, the acquisition of Rbs pathway can be advantageous for animal-associated microorganisms, such as *S. pealeana* (isolated from squid), since Rbs is abundant in animal cells as an important metabolic precursor.

***Sialic acid***, or *N-*acetylneuraminic acid (Nan) utilization gene cluster was identified only in a single *Shewanella* genome, *S. pealeana* (see additional data file 2). Nan is a nine-carbon monosaccharide that is often produсed in eukaryotes. It is catabolized by many commensal and pathogenic bacteria [9]. The Nan utilization gene locus in *S. pealeana* contains orthologs of the Nan catabolic (*nanEKA*) and regulatory (*nanR*) genes of *E. coli* (Fig. 3B, see also additional data file 2). In addition, the *nan* gene cluster encodes paralogs of the NagA and NagB enzymes that are shared with the Nag pathway and involved in the final steps of the Nan utilization pathway (Fig. 2). Two novel functional roles identified in the *Shewanella* Nan utilization pathway are the candidate Nan transporters NanP and Omp^Nan^ (Table 2). NanP is from the sodium:solute symporter superfamily (SSF), and it is similar to proline (PutP) and panthotenate (PanF) symporters. Orthologs of *nanP* are present within the Nan utilization loci in other bacterial genomes (e.g. in *Salmonella* and *Staphylococcus*). NanP is not homologous to the known sialic acid transporter NanT from *E. coli*. The predicted outer membrane transporter Omp^Nan^ in *Shewanella* is a functional equivalent of the Nan-inducible outer membrane porin NanC from *E. coli* [10]. The *omp^Nan^* gene is followed by the *nanM* gene encoding a periplasmic sialic acid mutarotase, which accelerates the equilibration of the α- and β-anomers of Nan [11]. Candidate NanR-binding sites identified upstream of the *nanPEK-nagB2*, *nagA2-nanA*, *omp^Nan^-nanM*, and *nanR* genes have a consensus sequence which is similar to that of NanR in *E. coli* (Fig. 3B). The ability of *S. pealeana* to grow on sialic acid was not tested in this study.

Phylogenetic analysis identified similar *nan* gene clusters in a single bacterium from the Alteromonadales lineage, *Pseudoalteromonas haloplanktis*, and in multiple species from the Enterobacteriales and Vibrionales lineages, suggesting their likely acquisition via LGT. The Nan catabolic pathway could be advantageous for marine animal-associated *S. pealeana* species because of sialic acids are abundant components of mucoproteins and glycoproteins, especially in animal tissue and blood.

***Alginate*** (Alg) utilization gene locus was identified only in one of the analyzed *Shewanella* genomes, *S. frigidimarina*. Alginate is a polysaccharide composed of β-D-mannuronate and α-L-guluronic acid residues and is a major cell wall constituent of brown seaweed, which commonly occurs in cold waters like those found off the coast of Aberdeen, Scottland where this strain was isolated.

This gene locus encodes two different alginate lyases AlgL1 and AlgL2, a hypothetical pectin utilization protein KdgF, 2-keto-3-deoxygluconate kinase KdgK, and two novel genes predicted to encode a mannuronate transporter AlgT and transcriptional regulator AlgR (Fig. 3B, see also additional data file 2). *S. frigidimarina* alginate lyases have candidate signal peptide cleavage sites, and they are likely secreted into the periplasm (Fig. 2). The predicted D-mannuronate transporter AlgT is similar to the D-galacturonate permease ExuT from *E. coli* (35% identity). The candidate Alg utilization regulator AlgR belongs to the GntR family, and it is similar to the regulator of D-galacturonate utilization ExuR from *E. coli* (35% identity). Candidate AlgR binding site identified upstream of the *algL1-algL2-kdgF-algT-kdgK* operon in *S. frigidimarina* resembles the ExuR binding site consensus of *E. coli* [12]. The ability of *S. frigidimarina* to grow on alginate is anticipated, but was not tested in this study.

Phylogenetic analysis of the Alg utilization genes suggests their likely acquisition by *S. frigidimarina* from other Alteromonadales species via LGT. For instance, the closest orthologs of *kdgK* were identified in *Pseudoalteromonas* and *Alteromonas* spp. These microorganisms, like *S. frigidimarina*, being isolated from various locations in the World Ocean, are characterized by their intrinsic ability to colonize algae, suggesting that the alginate catabolic gene cluster could be advantageous in this connection.

***Mannitol*** (Mtl) utilization pathway in *E. coli* and other Enterobacteria involves a mannitol-specific PTS transporter MtlA and mannitol-1-phosphate dehydrogenase MtlD [13]. A novel variant of the Mtl catabolic pathway was proposed based on the analysis of a gene cluster that was identified in a single *Shewanella* genome, *S. frigidimarina* (Fig. 3B, see also additional data file 2). This pathway in *S. frigidimarina* is proposed to proceed via a nonphosphorylating uptake by Mtl permease MtlP, followed by use of Mtl dehydrogenase MtlD^II^ and fructokinase MtlZ^II^. We predict that it is regulated by the novel DeoR-type transcriptional regulator MtlR^II^ (Table 2, Fig. 2). MtlD^II^ is similar (38% identity) to a Mtl dehydrogenase MtlK previously characterized in *Rhodobacter sphaeroides* [14]. The predicted fructokinase MtlZ^II^ belongs to the PfkB family of sugar kinases, and it shows ~30% similarity to the fructokinase ScrK from *E. coli*. The predicted mannitol permease MtlP belongs to the SSF transporter superfamily and it is most similar to the cytosine permease CodB from *E. coli* (23% identity). A comparative genomic reconstruction of the MtlR^II^ regulon allowed us to predict its candidate binding site located upstream of the *mtlP* gene (Fig. 3B). Phenotypic characterization of *Shewanella* showed a failure to grow on Mtl as a single carbon and energy source for all 10 analyzed species (Table 3). The inability of *S. frigidimarina* (type strain, NCIMB 400) to grow on mannitol could be attributed to the fact that its *mtl* gene locus is interrupted by a transposase inserted in opposite orientation immediately after the *mtlP* gene (Fig. 3B). The previous phenotypic analysis of *S. frigidimarina* sp. nov. (type strain, ACAM 591) demonstrated its ability to grow on mannitol as a sole carbon and energy source [15].

Phylogenetic analysis of *mtl* genes suggests their likely acquisition by *S. frigidimarina* from other Alteromonadales species via LGT. For instance, the closest orthologs of the *S. frigidimarina* *mtl* genes were identified in *Pseudoalteromonas haloplanktis*, a psychrophilic bacterium isolated from coastal sea water in Antarctic. Ecophysiological advantage of Mtl catabolic pathway for these marine bacteria could be related to their ability to colonize common brown algae that are characterized by a high accumulation of mannitol [16].

***Fructose*** (Fru) and ***xylose*** (Xyl) utilization pathways are absent from *Shewanella* spp. A genomic reconstruction of carbohydrate utilization pathways in 19 *Shewanella* species has not identified any candidate genes for utilization of D-xylose or D-fructose. Although some *Shewanella* spp have fructokinase genes in the context of either the sucrose or mannoside utilization pathways, we were unable to find candidate fructose transporters in any of the analyzed *Shewanella* species. Consistent with this analysis phenotypic profiling of 14 *Shewanella* species confirmed their inability to grow on either D-xylose or D-fructose as a carbon and energy source (see additional data file 7).

***Several putative carbohydrate utilization genes*** identified in *Shewanella* genomes have not been assigned a specific function in any sugar utilization pathway (see additional data files 3 and 5). As already discussed, most of *Shewanella* species have orthologs of the general PTS system genes *ptsH*, *ptsI*, and *crr* and the glucose-specific PTS transporter *ptsG* of *E. coli*, although their physiological role remains unclear.

Among candidate carbohydrate utilization genes conserved in most *Shewanella* genomes are three genes encoding putative sugar kinases of unknown specificity. The *SO0810* gene, a homolog of *E. coli* ribokinase RbsK from the PfkB family is located in an operon with a nucleoside hydrolase (SO0811). Altogether these two genes and the nucleoside-specific channel protein Tsx (SO3141) likely comprise the nucleoside utilization pathway. This conjecture, which is consistent with the known ability of *S. oneidensis* to grow on inosine [17] and DNA [18], remains a subject of further experimental testing. The *SO4458* and *SO1389* genes encoding hypothetical sugar kinases from the PfkB and ROK families, respectively, have not been assigned to any specific metabolic pathway due to the lack of any suggestive genomic context.

All four *S. baltica* strains and *S. woodyi* possess a hypothetical sugar utilization gene cluster (e.g., *Sbal_1406-1411*) encoding a hypothetical sugar kinase from the ROK family, a paralog of *N-*acetylglucosamine transporter NagP from the GGP family, a paralog of β-hexosaminidase Hex, a hypothetical polysaccharide deacetylase, and a hypothetical transcriptional regulator from the AraC family. The presence of Hex and NagP paralogs points to a likely functional association with the Nag pathway, however the exact functions of these proteins could not be proposed without additional experiments.

**References**

1. Tong S, Porco A, Isturiz T, Conway T: **Cloning and molecular genetic characterization of the *Escherichia coli* gntR, gntK, and gntU genes of GntI, the main system for gluconate metabolism**. *J Bacteriol* 1996, **178**:3260-3269.

2. Ray WK, Larson TJ: **Application of AgaR repressor and dominant repressor variants for verification of a gene cluster involved in N-acetylgalactosamine metabolism in *Escherichia coli* K-12**. *Mol Microbiol* 2004, **51**:813-826.

3. Boos W, Ehmann U, Forkl H, Klein W, Rimmele M, Postma P: **Trehalose transport and metabolism in *Escherichia coli***. *J Bacteriol* 1990, **172**:3450-3461.

4. Itoh T, Mikami B, Hashimoto W, Murata K: **Crystal structure of YihS in complex with D-mannose: structural annotation of *Escherichia coli* and *Salmonella enterica* yihS-encoded proteins to an aldose-ketose isomerase**. *J Mol Biol* 2008, **377**:1443-1459.

5. Doten RC, Mortlock RP: **Inducible xylitol dehydrogenases in enteric bacteria**. *J Bacteriol* 1985, **162**:845-848.

6. Lin ECC: **Xylitol is in Nature**. *BioScience* 1977, **27**:4.

7. Leonardo MR, Moser DP, Barbieri E, Brantner CA, MacGregor BJ, Paster BJ, Stackebrandt E, Nealson KH: ***Shewanella pealeana* sp. nov., a member of the microbial community associated with the accessory nidamental gland of the squid Loligo pealei**. *Int J Syst Bacteriol* 1999, **49**:1341-1351.

8. Zhao JS, Manno D, Leggiadro C, O'Neil D, Hawari J: ***Shewanella halifaxensis* sp. nov., a novel obligately respiratory and denitrifying psychrophile**. *Int J Syst Evol Microbiol* 2006, **56**:205-212.

9. Vimr ER, Kalivoda KA, Deszo EL, Steenbergen SM: **Diversity of microbial sialic acid metabolism**. *Microbiol Mol Biol Rev* 2004, **68**:132-153.

10. Condemine G, Berrier C, Plumbridge J, Ghazi A: **Function and expression of an N-acetylneuraminic acid-inducible outer membrane channel in Escherichia coli**. *J Bacteriol* 2005, **187**:1959-1965.

11. Severi E, Muller A, Potts JR, Leech A, Williamson D, Wilson KS, Thomas GH: **Sialic acid mutarotation is catalyzed by the *Escherichia coli* beta-propeller protein YjhT**. *J Biol Chem* 2008, **283**:4841-4849.

12. Rodionov DA, Mironov AA, Rakhmaninova AB, Gelfand MS: **Transcriptional regulation of transport and utilization systems for hexuronides, hexuronates and hexonates in gamma purple bacteria**. *Mol Microbiol* 2000, **38**:673-683.

13. Otte S, Lengeler JW: **The *mtl* genes and the mannitol-1-phosphate dehydrogenase from *Klebsiella pneumoniae* KAY2026**. *FEMS Microbiol Lett* 2001, **194**:221-227.

14. Brunker P, Altenbuchner J, Mattes R: **Structure and function of the genes involved in mannitol, arabitol and glucitol utilization from *Pseudomonas fluorescens* DSM50106**. *Gene* 1998, **206**:117-126.

15. Bowman JP, McCammon SA, Nichols DS, Skerratt JH, Rea SM, Nichols PD, McMeekin TA: ***Shewanella gelidimarina* sp. nov. and *Shewanella frigidimarina* sp. nov., novel Antarctic species with the ability to produce eicosapentaenoic acid (20:5 omega 3) and grow anaerobically by dissimilatory Fe(III) reduction**. *Int J Syst Bacteriol* 1997, **47**:1040-1047.

16. Yamaguchi T IT, Nisizawa K: **Pathway of mannitol formation during photosynthesis in brown algae**. *Plant and Cell Physiology* 1969, **10**:425-440.

17. Driscoll ME, Romine MF, Juhn FS, Serres MH, McCue LA, Beliaev AS, Fredrickson JK, Gardner TS: **Identification of diverse carbon utilization pathways in *Shewanella oneidensis* MR-1 via expression profiling**. *Genome Inform* 2007, **18**:287-298.

18. Pinchuk GE, Ammons C, Culley DE, Li SM, McLean JS, Romine MF, Nealson KH, Fredrickson JK, Beliaev AS: **Utilization of DNA as a sole source of phosphorus, carbon, and energy by *Shewanella* spp.: ecological and physiological implications for dissimilatory metal reduction**. *Appl Environ Microbiol* 2008, **74**:1198-1208.
