# Supplementary material for: Genomic encyclopedia of sugar utilization pathways in the Shewanella genus
Source: BMC Genomics. 2010 Sep 13;11:494. doi: 10.1186/1471-2164-11-494 (PMC2996990; doi:10.1186/1471-2164-11-494)
Supplement: Additional file 6 — Experimental verification of novel enzymes, transporters, and regulators involved in sugar utilization in Shewanella. A. Phenotypic characterization of glcPMal (Shewana3_2310) for its involvement in glucose utilization in Shewanella sp. ANA-3; B. Phenotypic characterization of nagP (SO3503) for its involvement in N-acetylglucosamine (Nag) utilization in Shewanella oneidensis MR-1; C. Phenotypic characterization of grtP (SO1771) for its involvement in D-glycerate; D. Complementation of the E. coli cellobiose utilization by the bglA-bglT (Sbal_1133-1132) genes from S. baltica OS155; E. Substrate specificity of Shewanella baltica OS155 GlkII (Sbal_1134) kinase; F. Growth of E. coli DH5a strain (Scr-) containing heterologously expressed sucrose utilization genes scrTII-scrP (Sfri_3989-3990) from S. frigidimarina. [file 1471-2164-11-494-S6.PPT]

## Slide 1
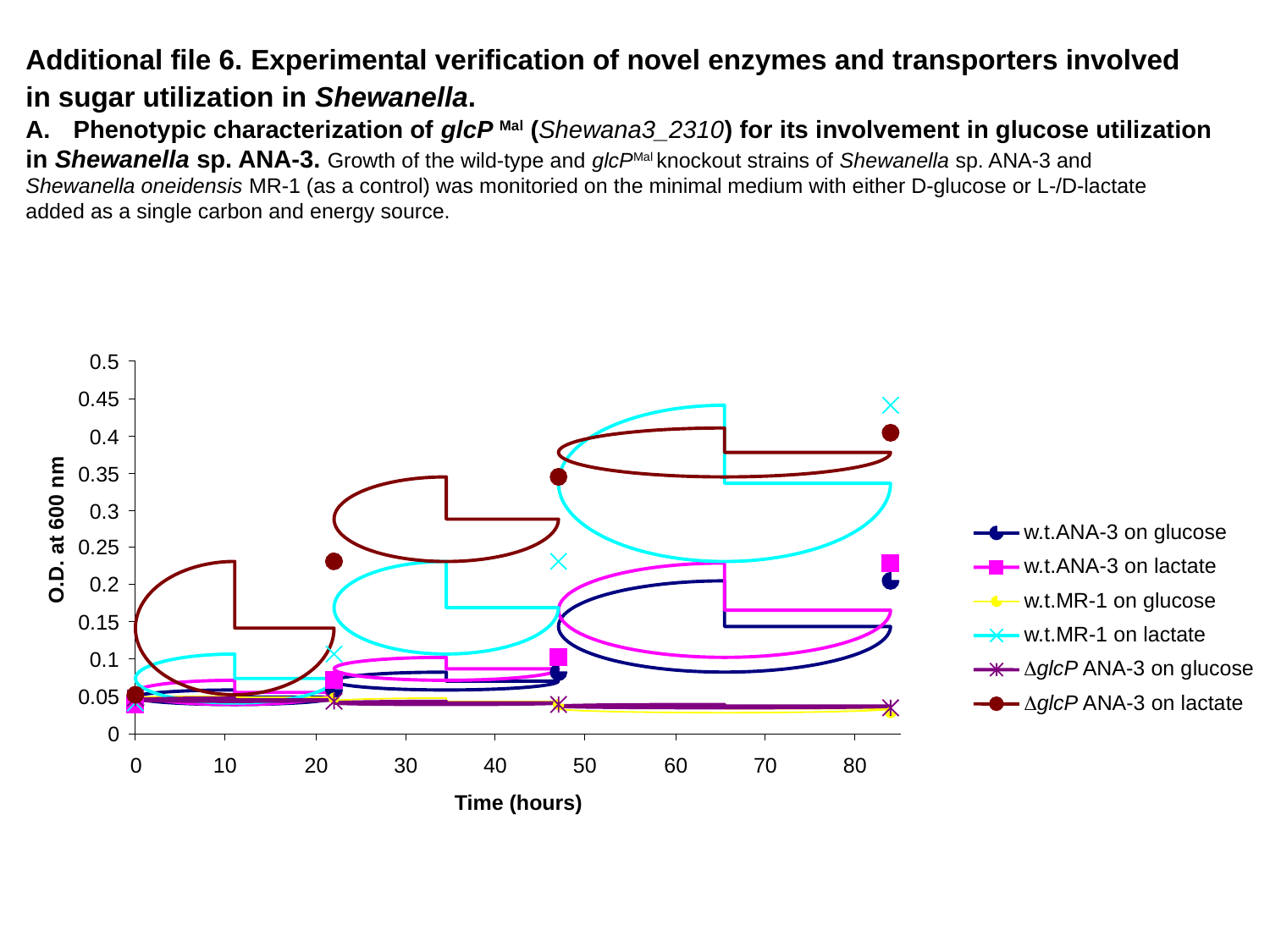

Additional file 6. Experimental verification of novel enzymes and transporters involved
in sugar utilization in Shewanella.
Phenotypic characterization of glcP Mal (Shewana3_2310) for its involvement in glucose utilization
in Shewanella sp. ANA-3. Growth of the wild-type and glcPMal knockout strains of Shewanella sp. ANA-3 and
Shewanella oneidensis MR-1 (as a control) was monitoried on the minimal medium with either D-glucose or L-/D-lactate
added as a single carbon and energy source.
0.5
0.45
0.4
0.35
0.3
O.D. at 600 nm
w.t.ANA-3 on glucose
0.25
w.t.ANA-3 on lactate
0.2
w.t.MR-1 on glucose
0.15
w.t.MR-1 on lactate
0.1
ΔglcP ANA-3 on glucose
0.05
ΔglcP ANA-3 on lactate
0
0
10
20
30
40
50
60
70
80
Time (hours)

## Slide 2
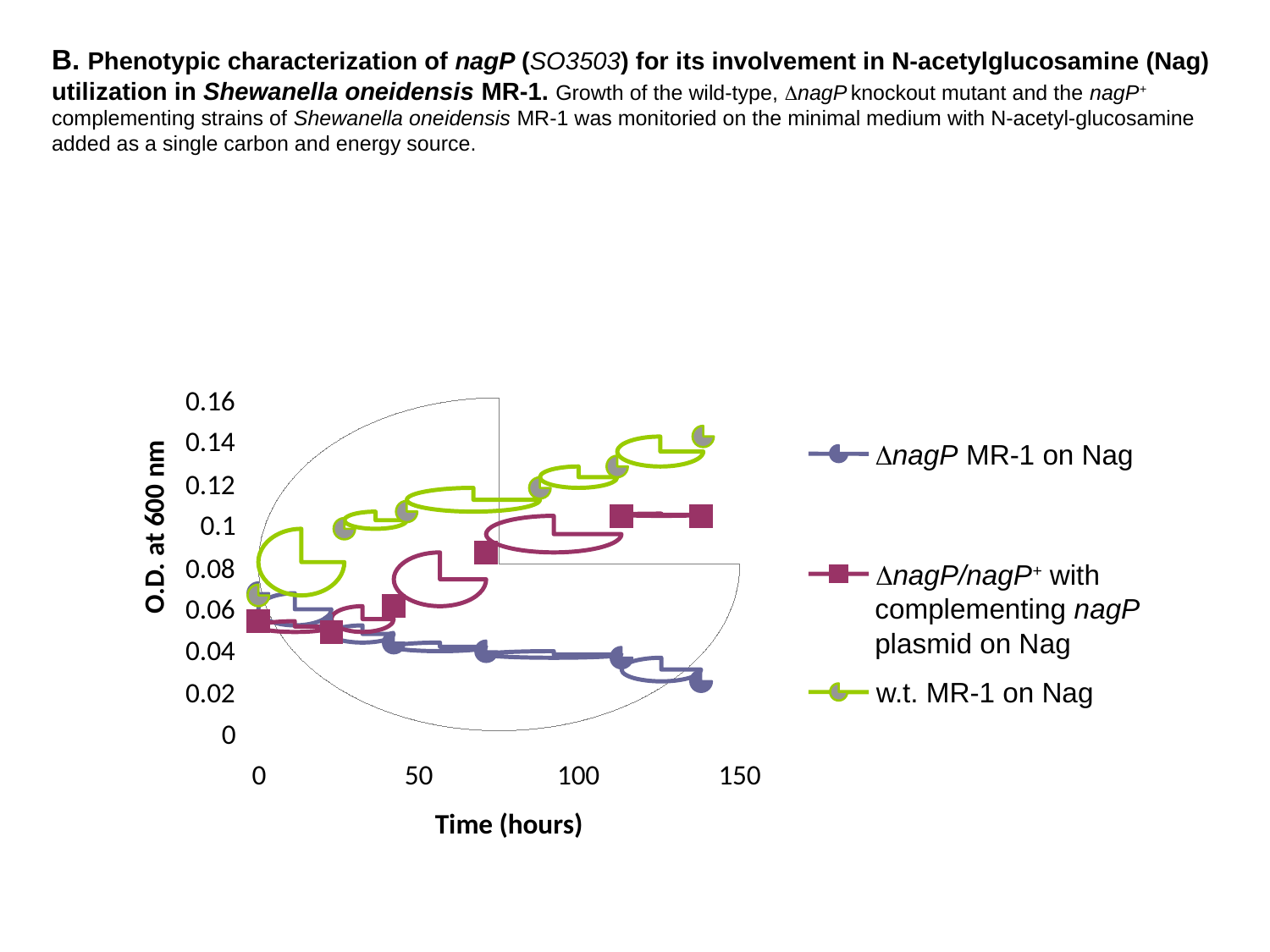

B. Phenotypic characterization of nagP (SO3503) for its involvement in N-acetylglucosamine (Nag)
utilization in Shewanella oneidensis MR-1. Growth of the wild-type, ΔnagP knockout mutant and the nagP+
complementing strains of Shewanella oneidensis MR-1 was monitoried on the minimal medium with N-acetyl-glucosamine
added as a single carbon and energy source.
0.16
0.14
ΔnagP MR-1 on Nag
0.12
0.1
O.D. at 600 nm
0.08
ΔnagP/nagP+ with
0.06
complementing nagP
plasmid on Nag
0.04
0.02
w.t. MR-1 on Nag
0
0
50
100
150
Time (hours)

## Slide 3
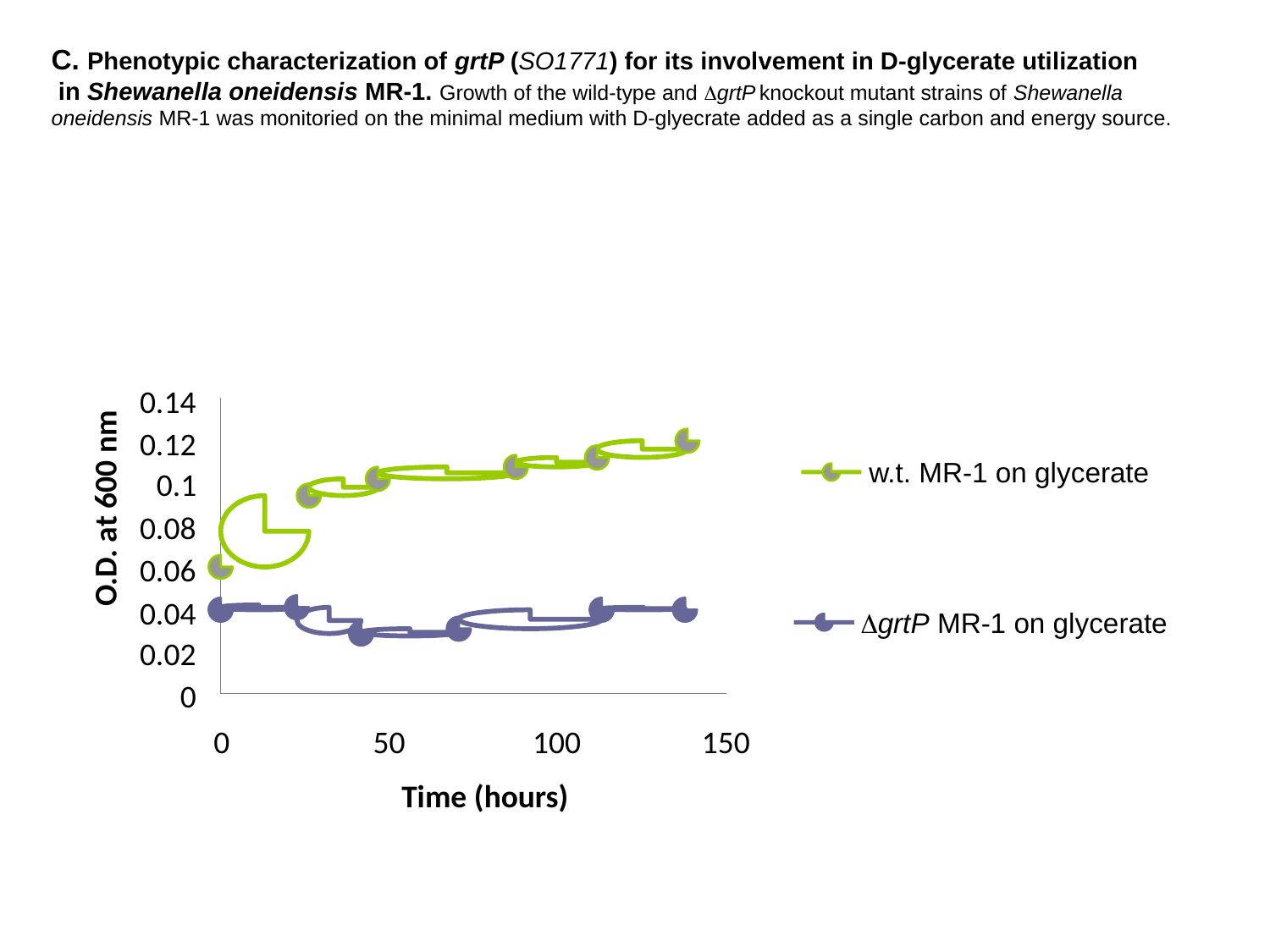

C. Phenotypic characterization of grtP (SO1771) for its involvement in D-glycerate utilization
 in Shewanella oneidensis MR-1. Growth of the wild-type and ΔgrtP knockout mutant strains of Shewanella
oneidensis MR-1 was monitoried on the minimal medium with D-glyecrate added as a single carbon and energy source.
0.14
0.12
w.t. MR-1 on glycerate
0.1
O.D. at 600 nm
0.08
0.06
0.04
ΔgrtP MR-1 on glycerate
0.02
0
0
50
100
150
Time (hours)

## Slide 4
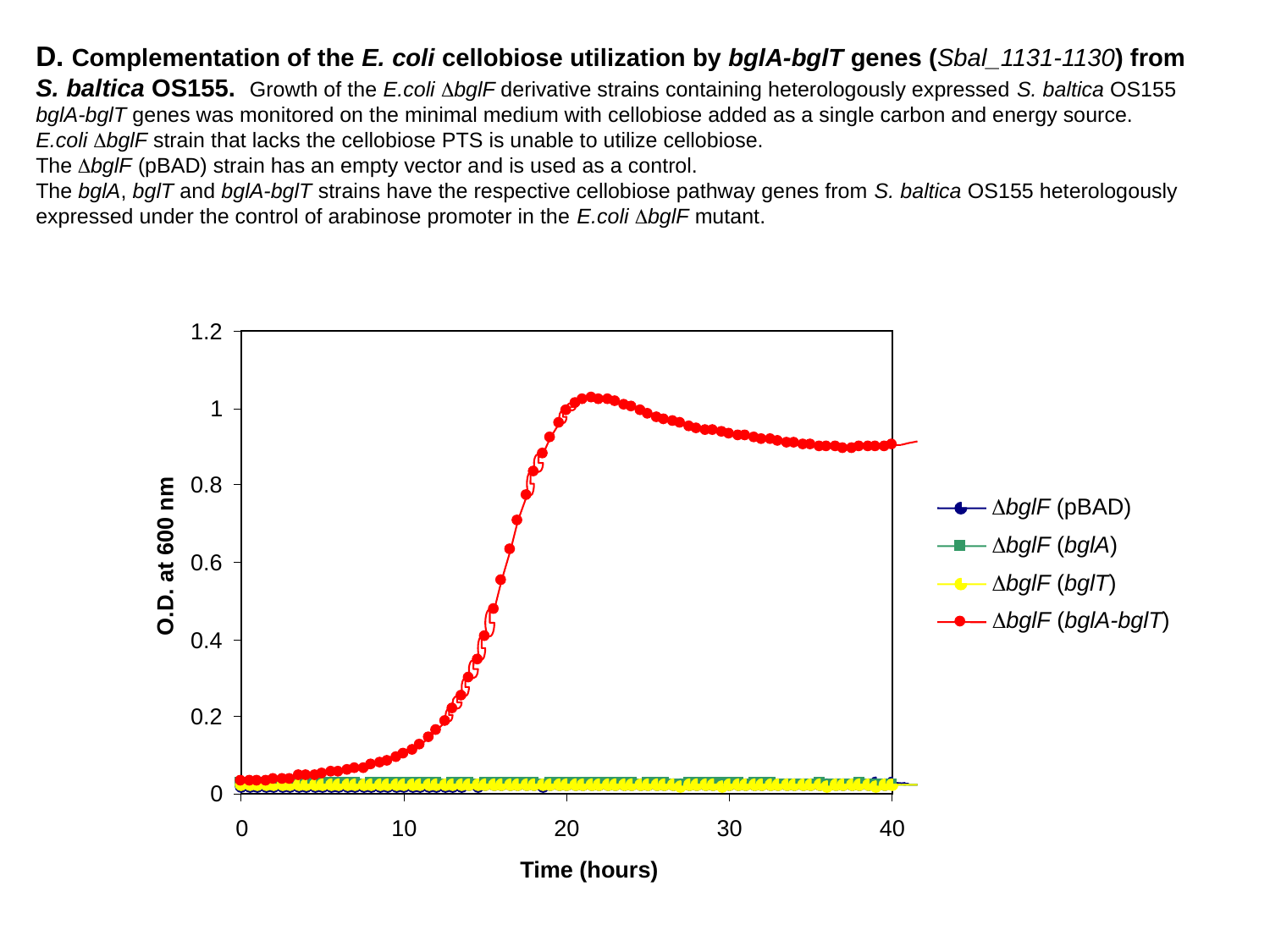

D. Complementation of the E. coli cellobiose utilization by bglA-bglT genes (Sbal_1131-1130) from
S. baltica OS155. Growth of the E.coli ΔbglF derivative strains containing heterologously expressed S. baltica OS155
bglA-bglT genes was monitored on the minimal medium with cellobiose added as a single carbon and energy source.
E.coli ΔbglF strain that lacks the cellobiose PTS is unable to utilize cellobiose.
The ΔbglF (pBAD) strain has an empty vector and is used as a control.
The bglA, bglT and bglA-bglT strains have the respective cellobiose pathway genes from S. baltica OS155 heterologously
expressed under the control of arabinose promoter in the E.coli ΔbglF mutant.
1.2
1
0.8
ΔbglF (pBAD)
ΔbglF (bglA)
O.D. at 600 nm
0.6
ΔbglF (bglT)
ΔbglF (bglA-bglT)
0.4
0.2
0
0
10
20
30
40
Time (hours)

## Slide 5
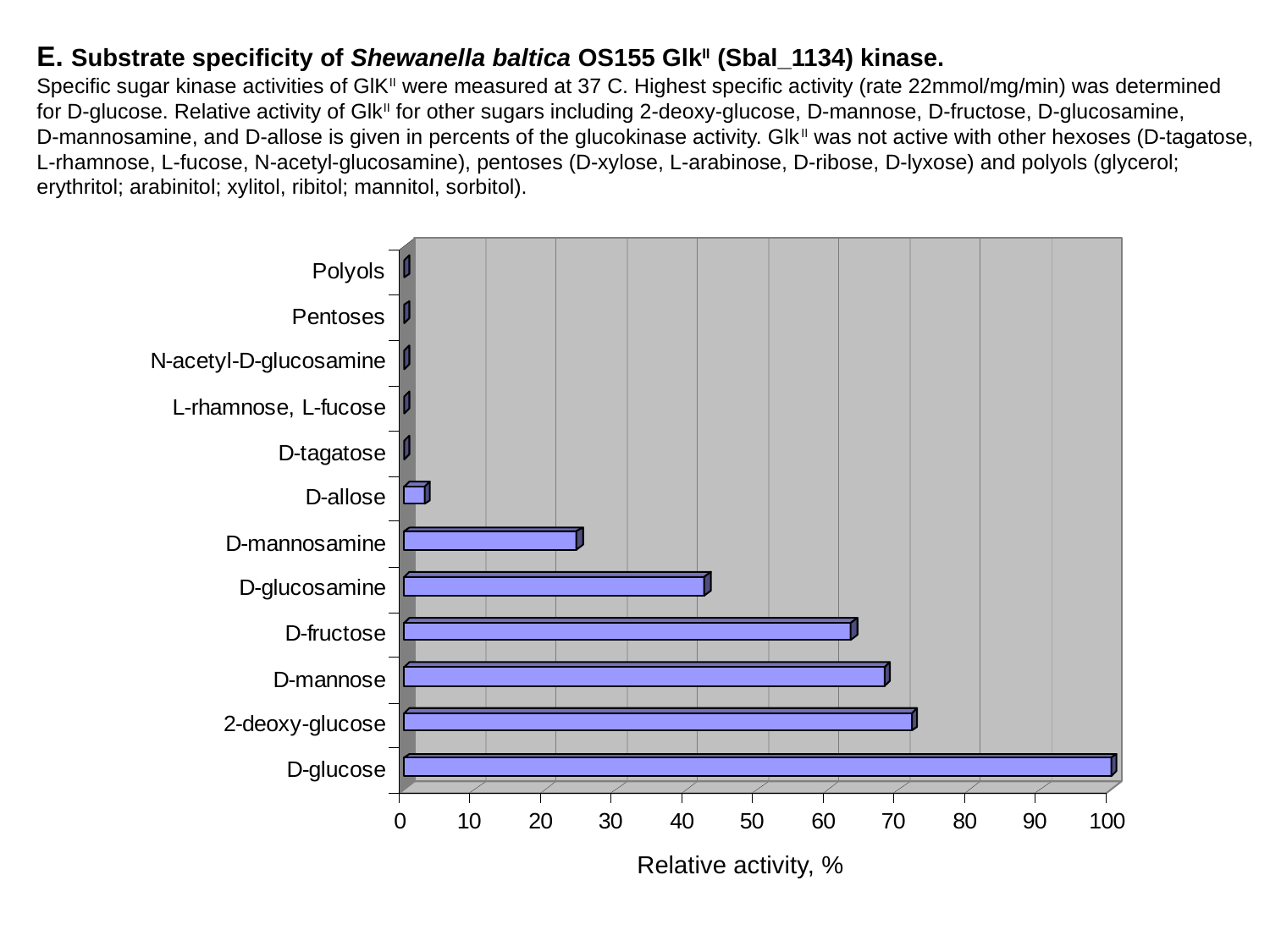

E. Substrate specificity of Shewanella baltica OS155 GlkII (Sbal_1134) kinase.
Specific sugar kinase activities of GlKII were measured at 37 C. Highest specific activity (rate 22mmol/mg/min) was determined
for D-glucose. Relative activity of GlkII for other sugars including 2-deoxy-glucose, D-mannose, D-fructose, D-glucosamine,
D-mannosamine, and D-allose is given in percents of the glucokinase activity. GlkII was not active with other hexoses (D-tagatose,
L-rhamnose, L-fucose, N-acetyl-glucosamine), pentoses (D-xylose, L-arabinose, D-ribose, D-lyxose) and polyols (glycerol;
erythritol; arabinitol; xylitol, ribitol; mannitol, sorbitol).
Relative activity, %

## Slide 6
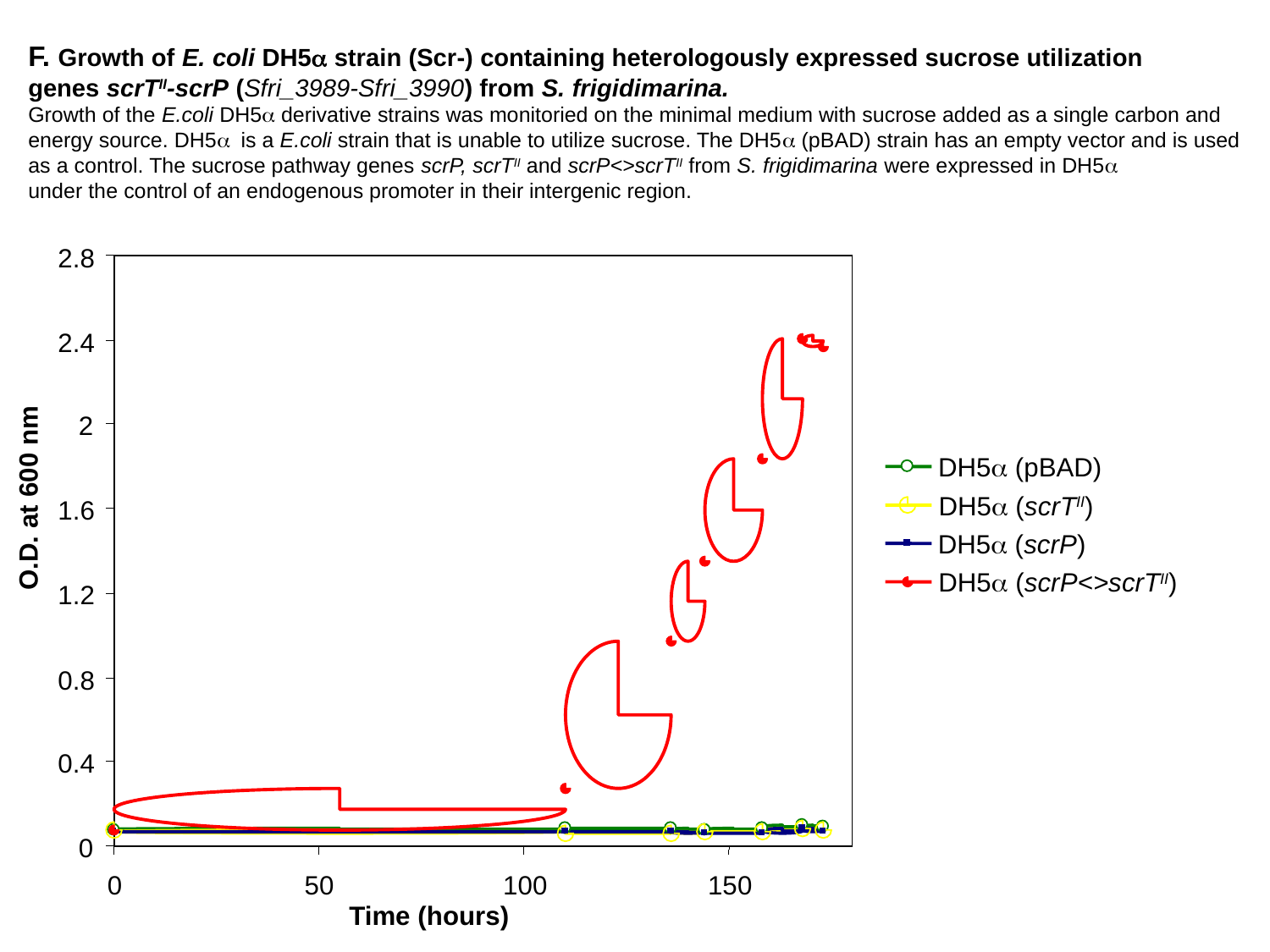

F. Growth of E. coli DH5α strain (Scr-) containing heterologously expressed sucrose utilization
genes scrTII-scrP (Sfri_3989-Sfri_3990) from S. frigidimarina.
Growth of the E.coli DH5α derivative strains was monitoried on the minimal medium with sucrose added as a single carbon and
energy source. DH5α is a E.coli strain that is unable to utilize sucrose. The DH5α (pBAD) strain has an empty vector and is used
as a control. The sucrose pathway genes scrP, scrTII and scrP<>scrTII from S. frigidimarina were expressed in DH5α
under the control of an endogenous promoter in their intergenic region.
2.8
2.4
2
DH5α (pBAD)
O.D. at 600 nm
DH5α (scrTII)
1.6
DH5α (scrP)
DH5α (scrP<>scrTII)
1.2
0.8
0.4
0
0
50
100
150
Time (hours)
