# Supplementary material for: Genomic encyclopedia of sugar utilization pathways in the Shewanella genus
Source: BMC Genomics. 2010 Sep 13;11:494. doi: 10.1186/1471-2164-11-494 (PMC2996990; doi:10.1186/1471-2164-11-494)
Supplement: Additional file 7 — Growth phenotypes of Shewanella on various carbon sources determined by manual assay. [file 1471-2164-11-494-S7.PPT]

## Slide 1
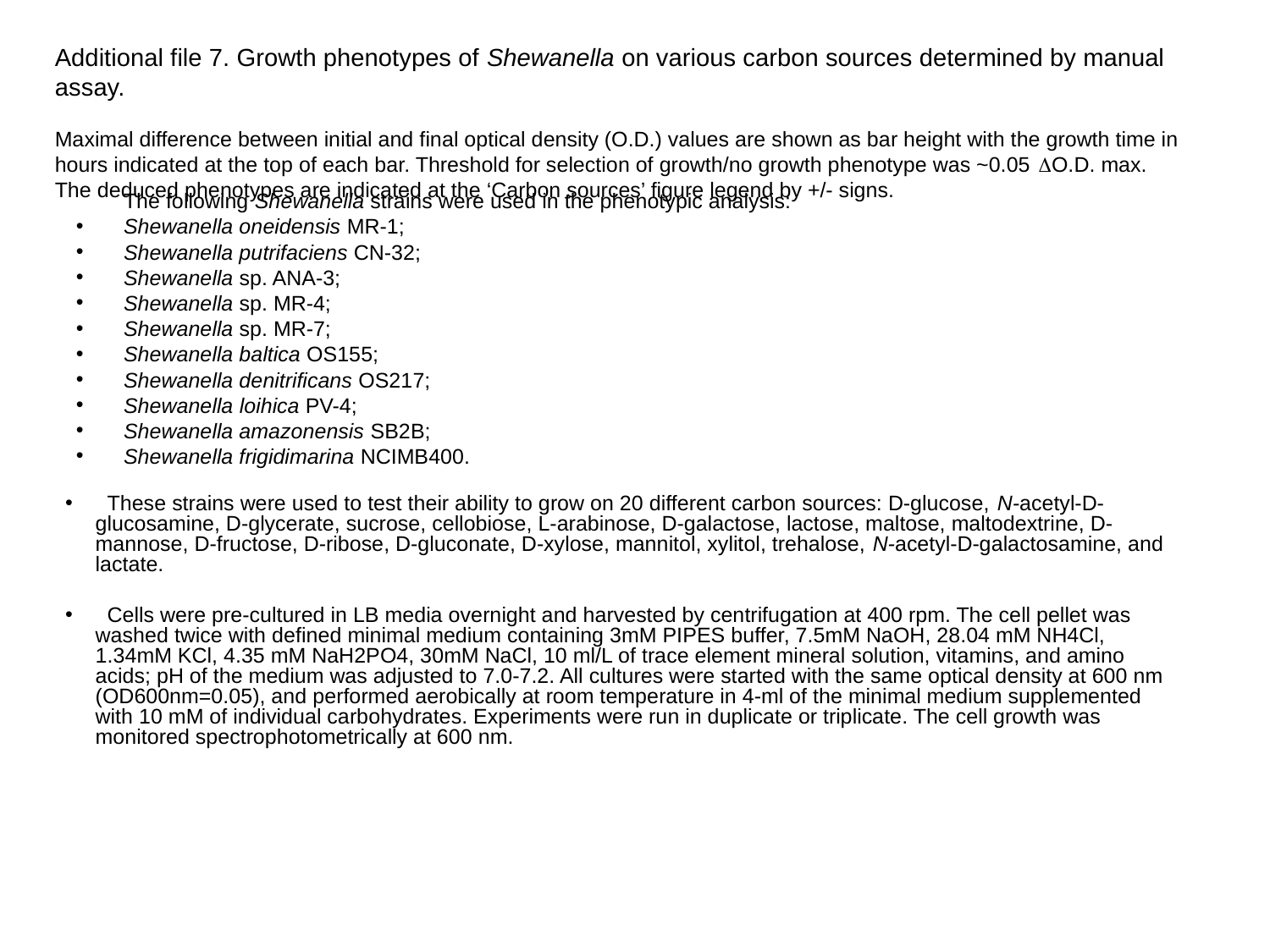

Additional file 7. Growth phenotypes of Shewanella on various carbon sources determined by manual assay.
Maximal difference between initial and final optical density (O.D.) values are shown as bar height with the growth time in
hours indicated at the top of each bar. Threshold for selection of growth/no growth phenotype was ~0.05 ΔO.D. max.
The deduced phenotypes are indicated at the ‘Carbon sources’ figure legend by +/- signs.
# The following Shewanella strains were used in the phenotypic analysis:
Shewanella oneidensis MR-1;
Shewanella putrifaciens CN-32;
Shewanella sp. ANA-3;
Shewanella sp. MR-4;
Shewanella sp. MR-7;
Shewanella baltica OS155;
Shewanella denitrificans OS217;
Shewanella loihica PV-4;
Shewanella amazonensis SB2B;
Shewanella frigidimarina NCIMB400.
 These strains were used to test their ability to grow on 20 different carbon sources: D-glucose, N-acetyl-D-glucosamine, D-glycerate, sucrose, cellobiose, L-arabinose, D-galactose, lactose, maltose, maltodextrine, D-mannose, D-fructose, D-ribose, D-gluconate, D-xylose, mannitol, xylitol, trehalose, N-acetyl-D-galactosamine, and lactate.
 Cells were pre-cultured in LB media overnight and harvested by centrifugation at 400 rpm. The cell pellet was washed twice with defined minimal medium containing 3mM PIPES buffer, 7.5mM NaOH, 28.04 mM NH4Cl, 1.34mM KCl, 4.35 mM NaH2PO4, 30mM NaCl, 10 ml/L of trace element mineral solution, vitamins, and amino acids; pH of the medium was adjusted to 7.0-7.2. All cultures were started with the same optical density at 600 nm (OD600nm=0.05), and performed aerobically at room temperature in 4-ml of the minimal medium supplemented with 10 mM of individual carbohydrates. Experiments were run in duplicate or triplicate. The cell growth was monitored spectrophotometrically at 600 nm.

## Slide 2
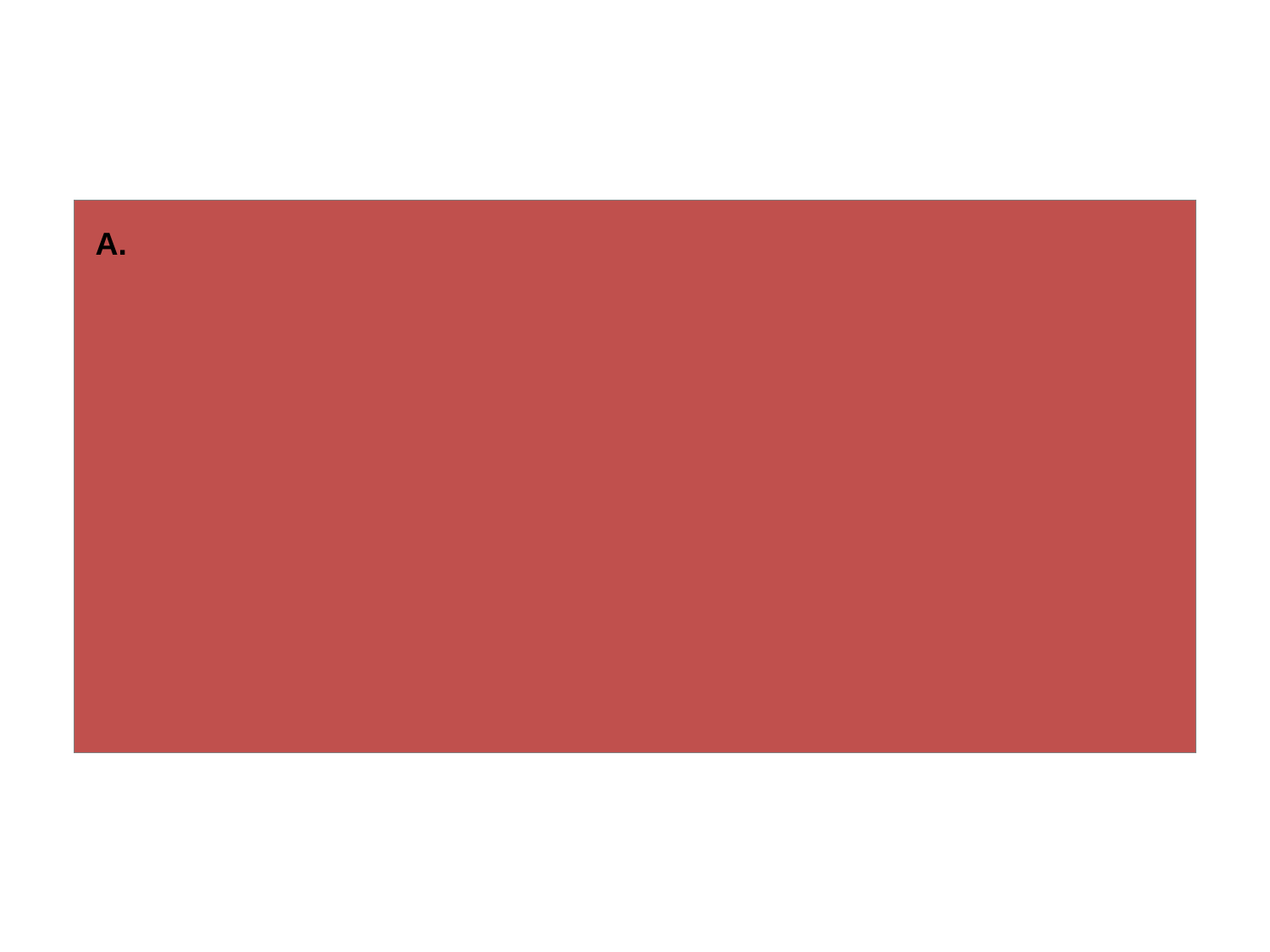

A.

## Slide 3
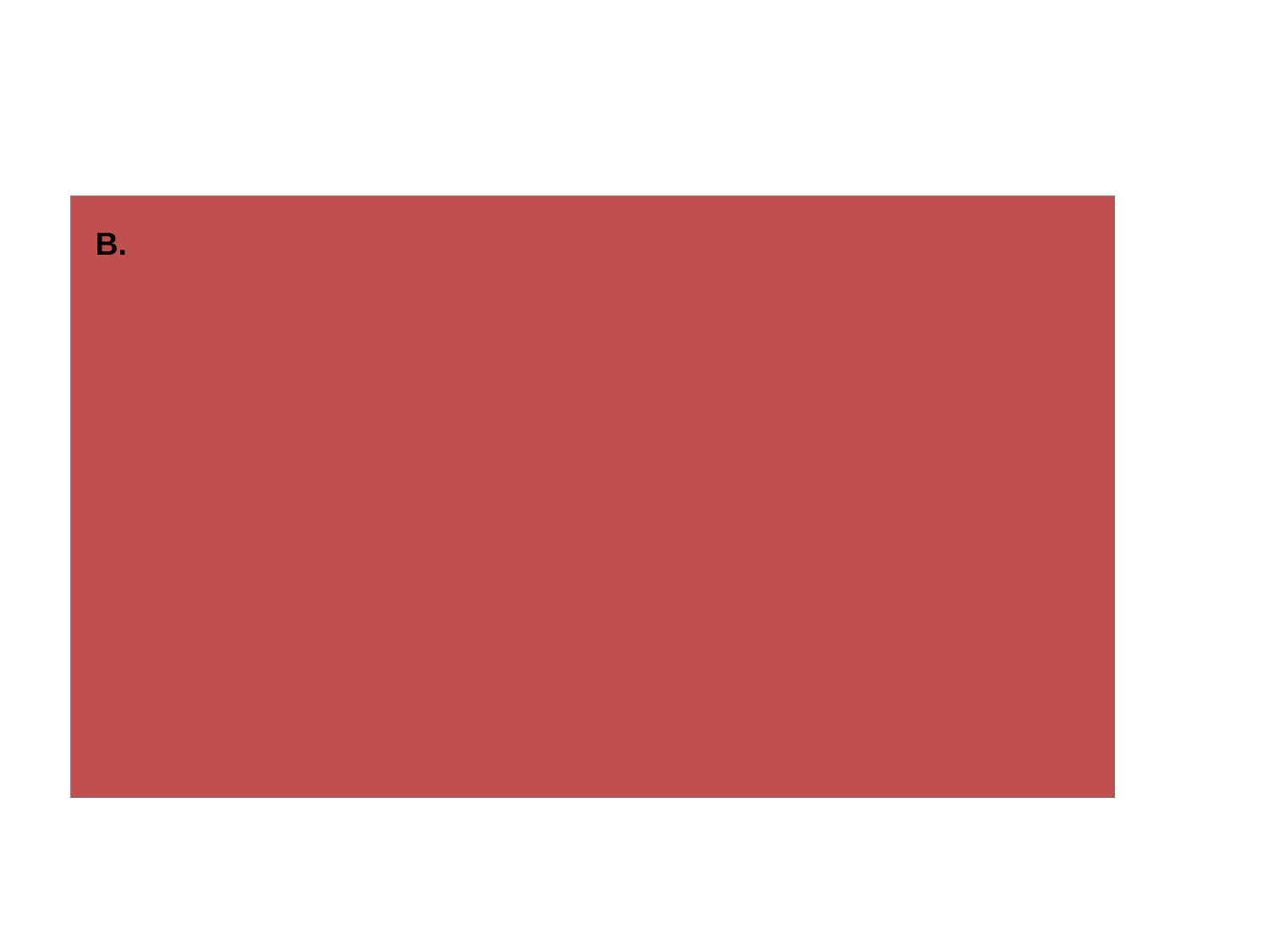

B.

## Slide 4
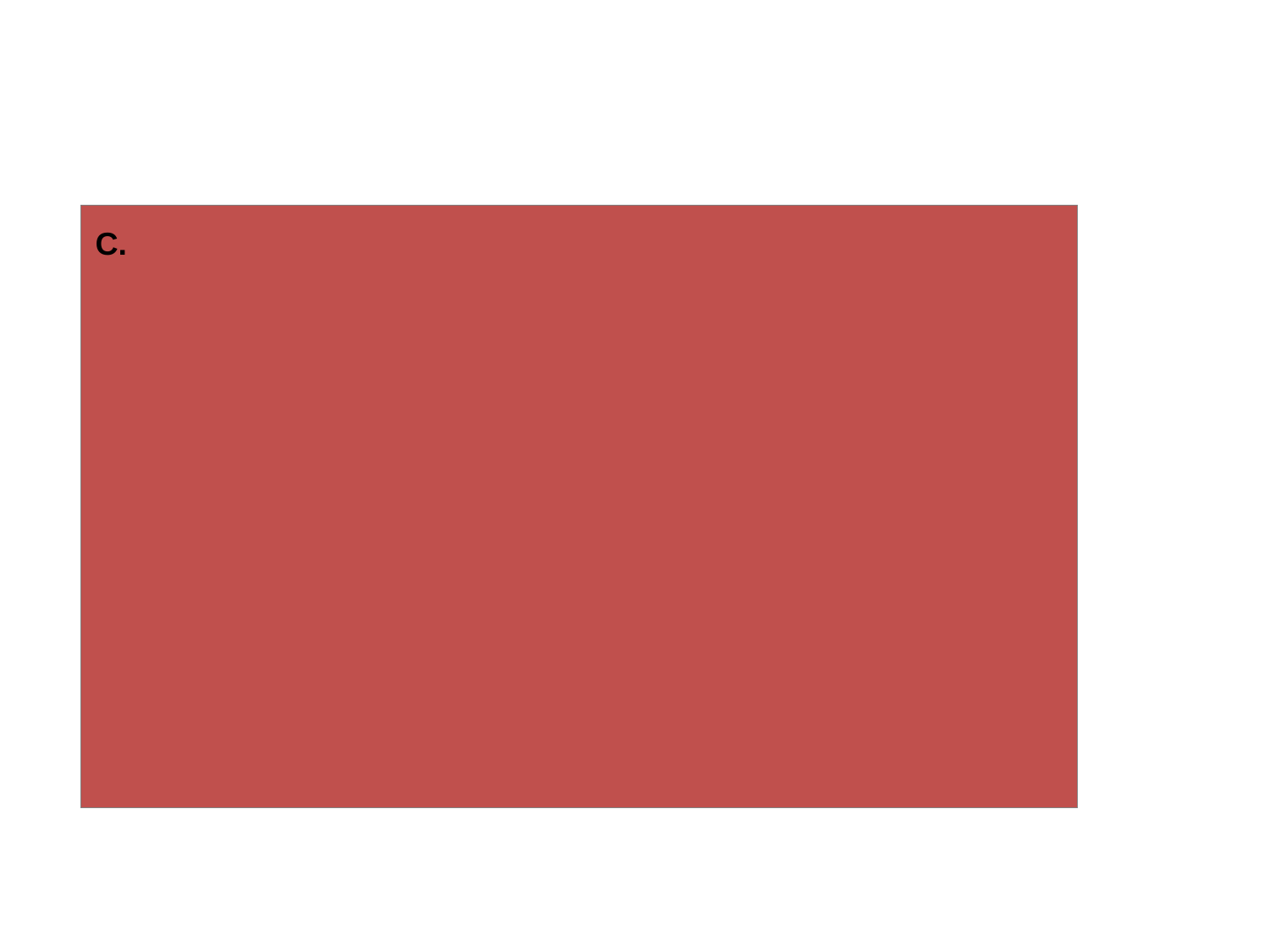

C.

## Slide 5
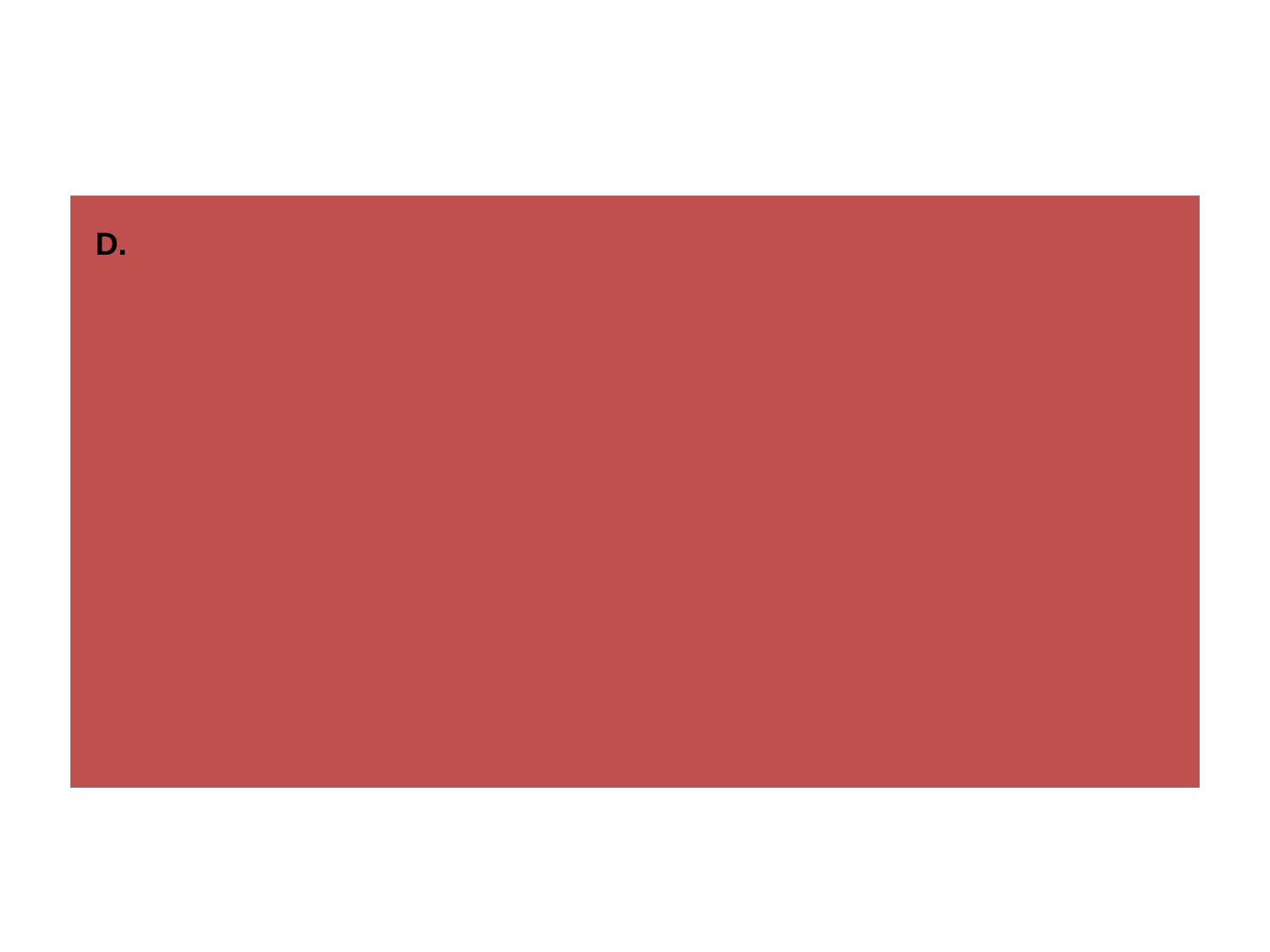

D.

## Slide 6
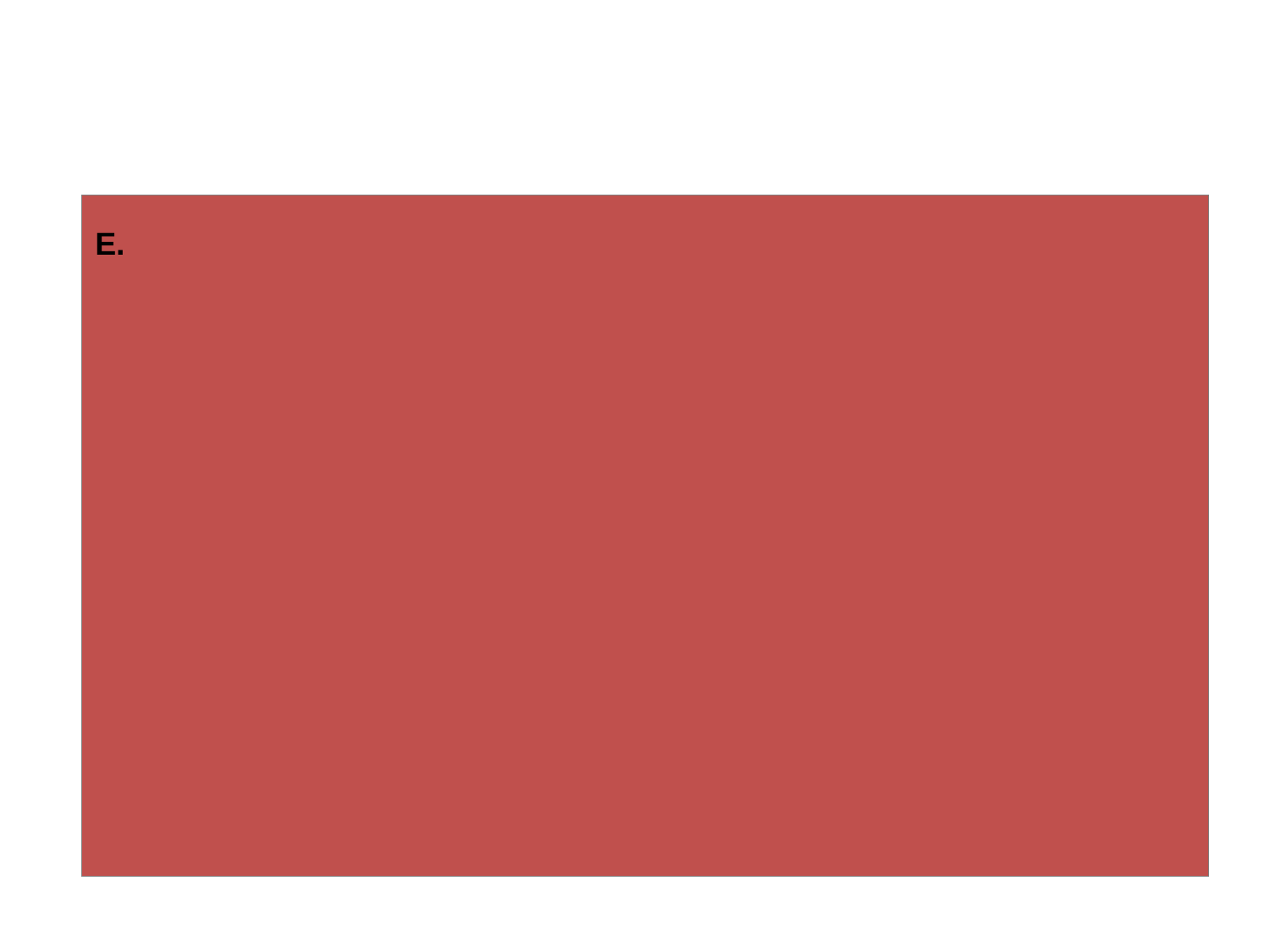

E.

## Slide 7
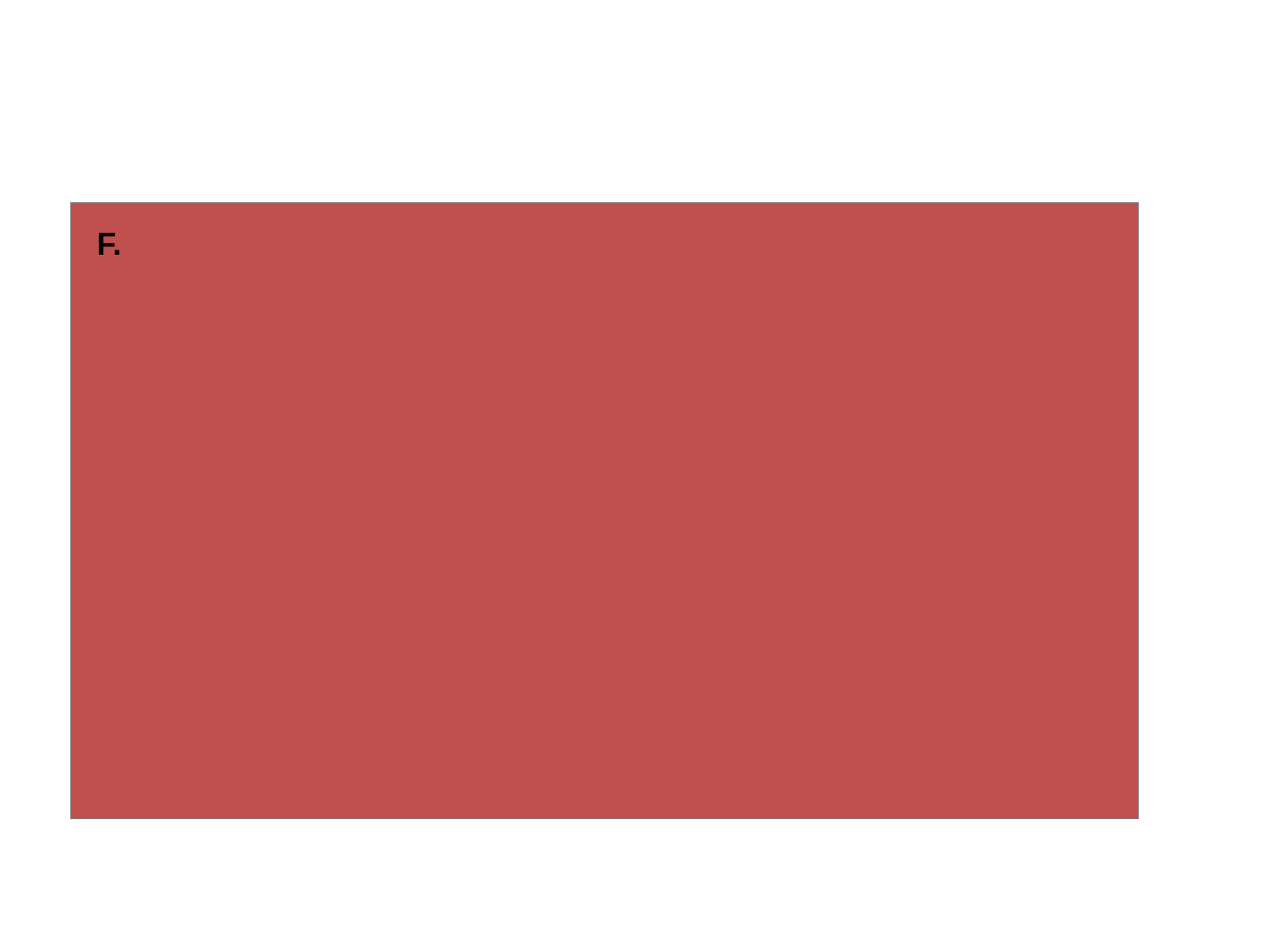

F.

## Slide 8
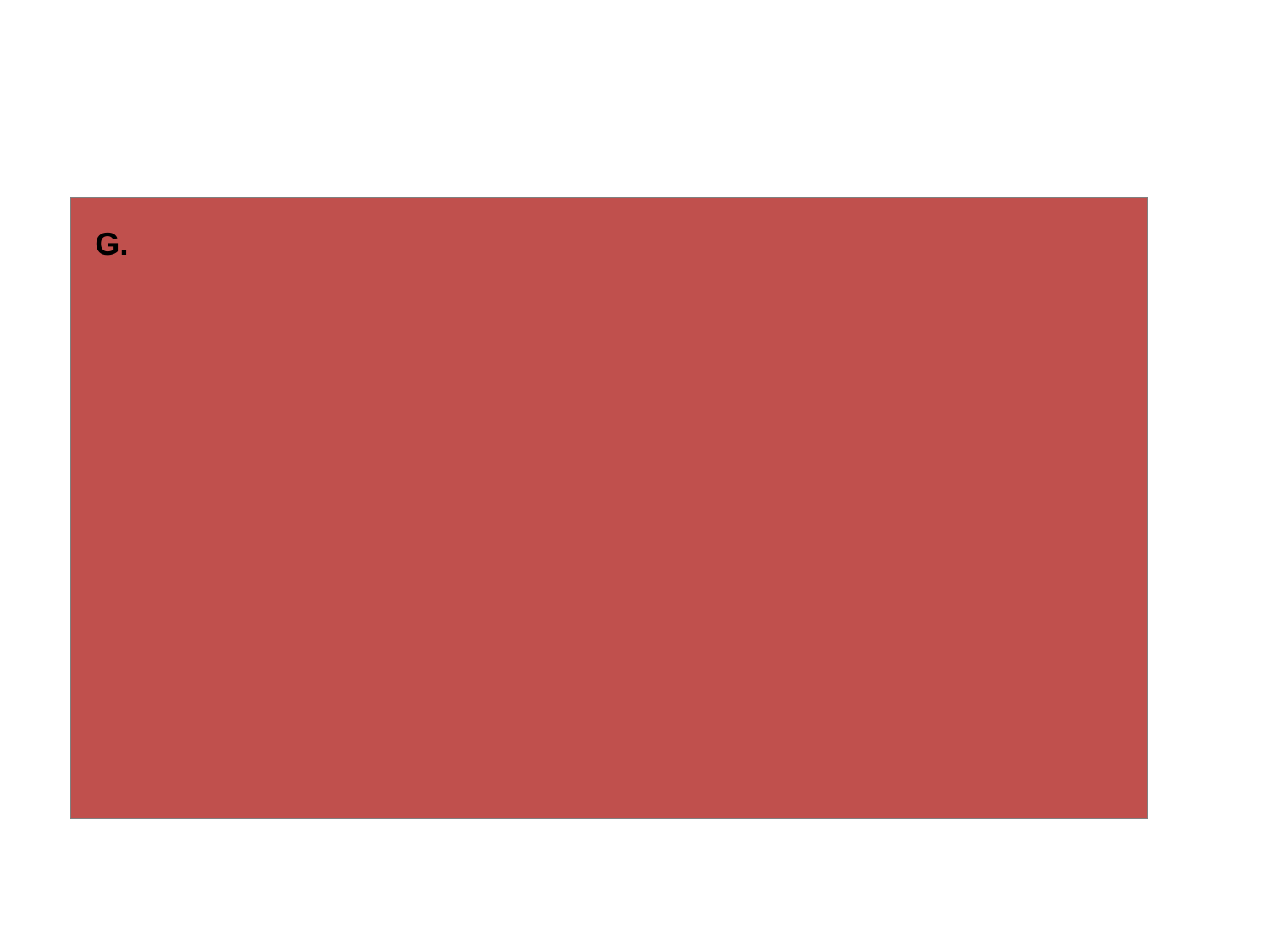

G.

## Slide 9
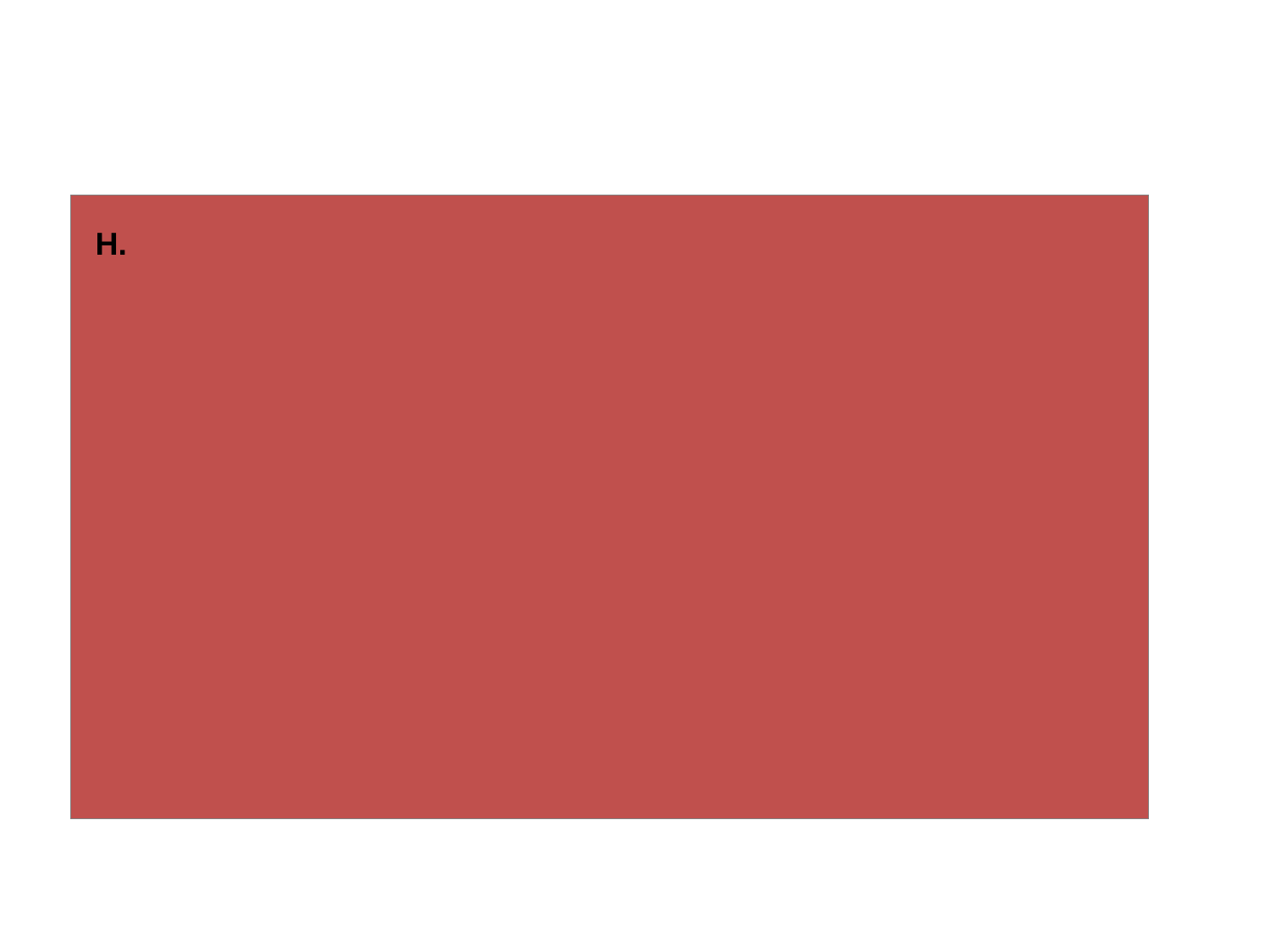

H.

## Slide 10
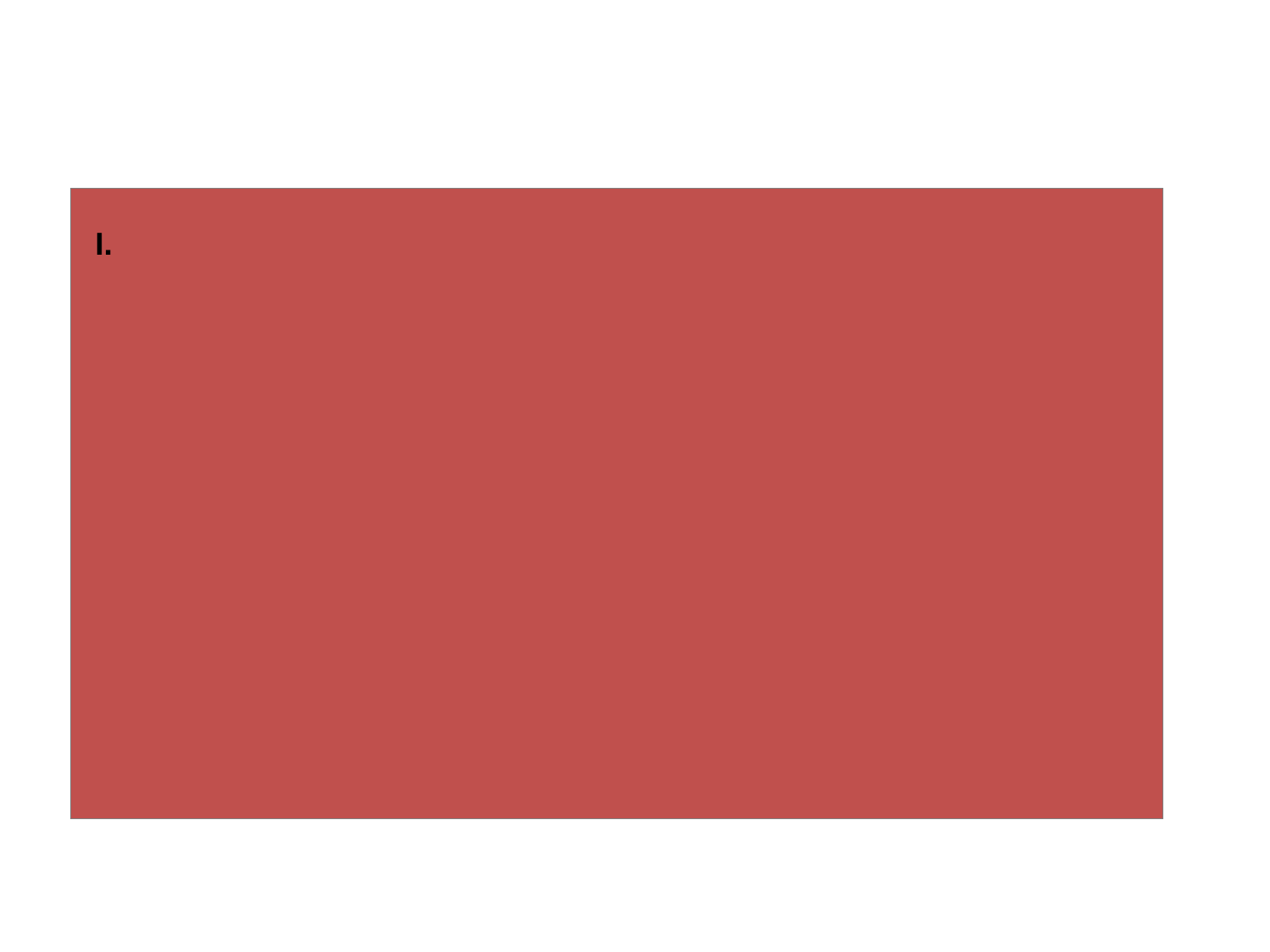

I.

## Slide 11
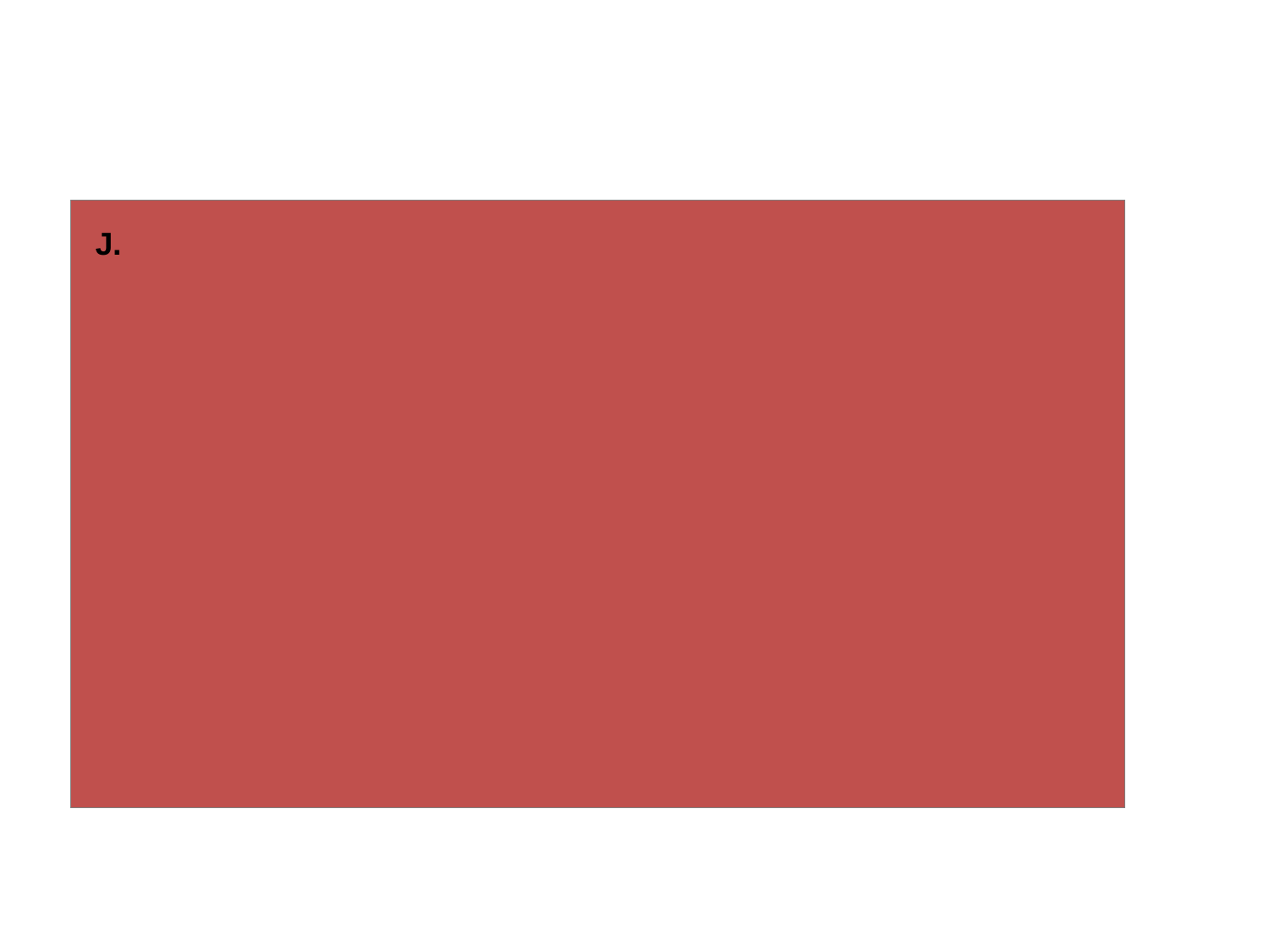

J.
