# Supplementary material for: Genomic encyclopedia of sugar utilization pathways in the Shewanella genus
Source: BMC Genomics. 2010 Sep 13;11:494. doi: 10.1186/1471-2164-11-494 (PMC2996990; doi:10.1186/1471-2164-11-494)
Supplement: Additional file 8 — Growth phenotypes of Shewanella on various carbon sources determined by microplate assay using Bioscreen C MBR system. [file 1471-2164-11-494-S8.PPT]

## Slide 1
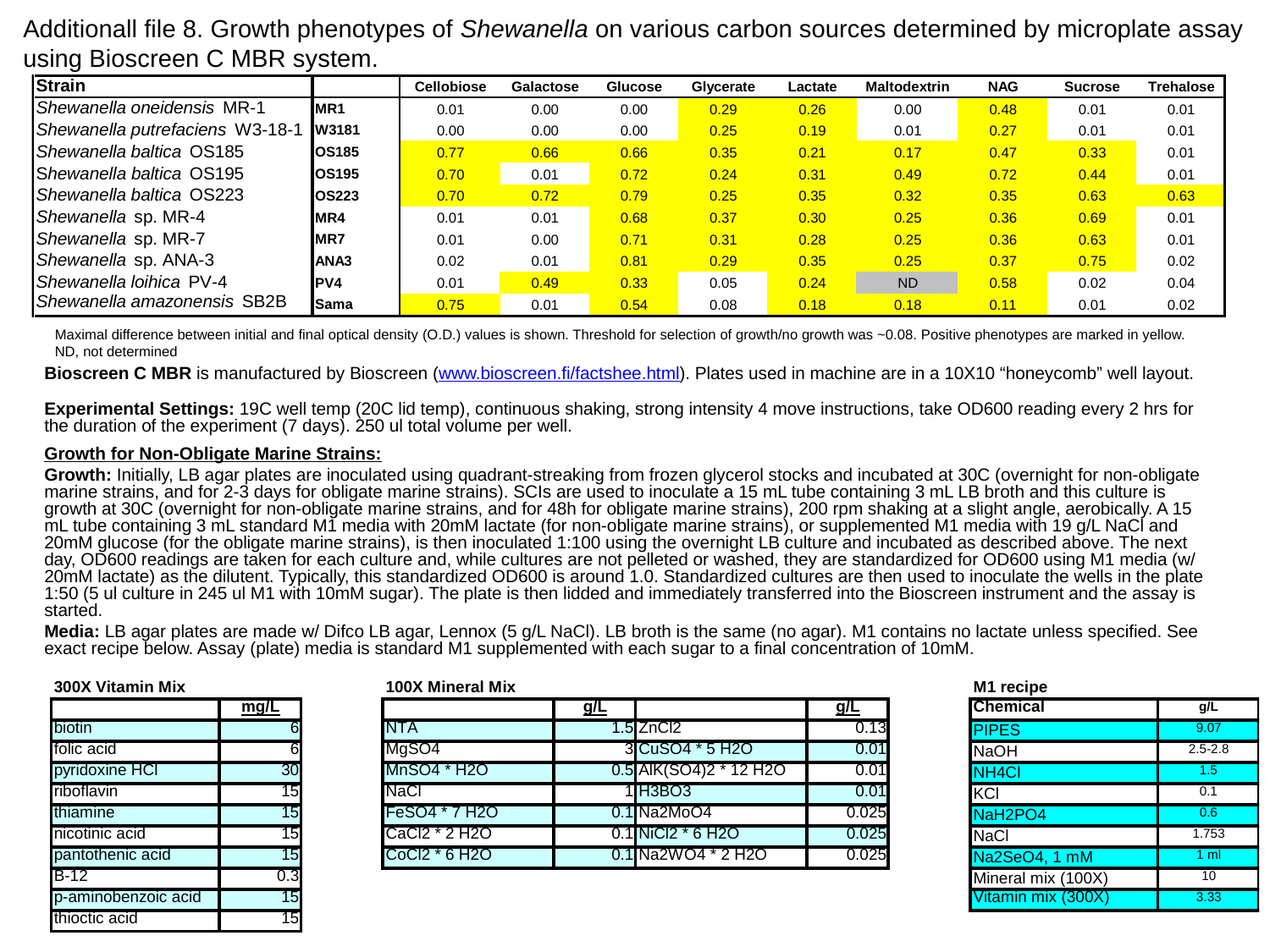

Additionall file 8. Growth phenotypes of Shewanella on various carbon sources determined by microplate assay using Bioscreen C MBR system.
Maximal difference between initial and final optical density (O.D.) values is shown. Threshold for selection of growth/no growth was ~0.08. Positive phenotypes are marked in yellow.
ND, not determined
Bioscreen C MBR is manufactured by Bioscreen (www.bioscreen.fi/factshee.html). Plates used in machine are in a 10X10 “honeycomb” well layout.
Experimental Settings: 19C well temp (20C lid temp), continuous shaking, strong intensity 4 move instructions, take OD600 reading every 2 hrs for the duration of the experiment (7 days). 250 ul total volume per well.
Growth for Non-Obligate Marine Strains:
Growth: Initially, LB agar plates are inoculated using quadrant-streaking from frozen glycerol stocks and incubated at 30C (overnight for non-obligate marine strains, and for 2-3 days for obligate marine strains). SCIs are used to inoculate a 15 mL tube containing 3 mL LB broth and this culture is growth at 30C (overnight for non-obligate marine strains, and for 48h for obligate marine strains), 200 rpm shaking at a slight angle, aerobically. A 15 mL tube containing 3 mL standard M1 media with 20mM lactate (for non-obligate marine strains), or supplemented M1 media with 19 g/L NaCl and 20mM glucose (for the obligate marine strains), is then inoculated 1:100 using the overnight LB culture and incubated as described above. The next day, OD600 readings are taken for each culture and, while cultures are not pelleted or washed, they are standardized for OD600 using M1 media (w/ 20mM lactate) as the dilutent. Typically, this standardized OD600 is around 1.0. Standardized cultures are then used to inoculate the wells in the plate 1:50 (5 ul culture in 245 ul M1 with 10mM sugar). The plate is then lidded and immediately transferred into the Bioscreen instrument and the assay is started.
Media: LB agar plates are made w/ Difco LB agar, Lennox (5 g/L NaCl). LB broth is the same (no agar). M1 contains no lactate unless specified. See exact recipe below. Assay (plate) media is standard M1 supplemented with each sugar to a final concentration of 10mM.
