# Supplementary material for: Genomic encyclopedia of sugar utilization pathways in the Shewanella genus
Source: BMC Genomics. 2010 Sep 13;11:494. doi: 10.1186/1471-2164-11-494 (PMC2996990; doi:10.1186/1471-2164-11-494)
Supplement: Additional file 9 — Comparison of reconstructed sugar utilization pathways in Shewanella and Enterobacteria. A. N-acetylglucosamine (Nag) and chitin utilization pathways; B. D-glycerate (Grt) and glucarate/galactarate utilization pathways; C. β-glucoside (Bgl) utilization pathway; D. Sucrose (Scr) utilization pathway; E. L-arabinose (Ara) and arabinosides utilization pathways. [file 1471-2164-11-494-S9.PDF]

Additional file 9. Comparison of reconstructed sugar utilization pathways in *Shewanella* and Enterobacteria.

A. N-acetylglucosamine (Nag) and chitin utilization pathways.

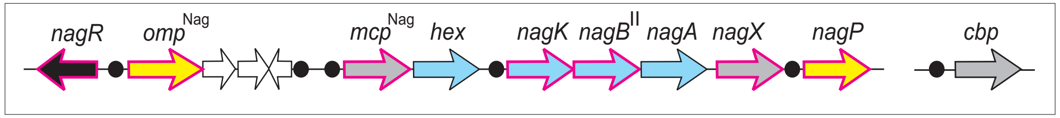

[*Shewanella oneidensis*]

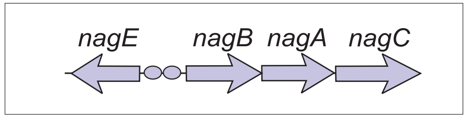

[*Escherichia coli*]

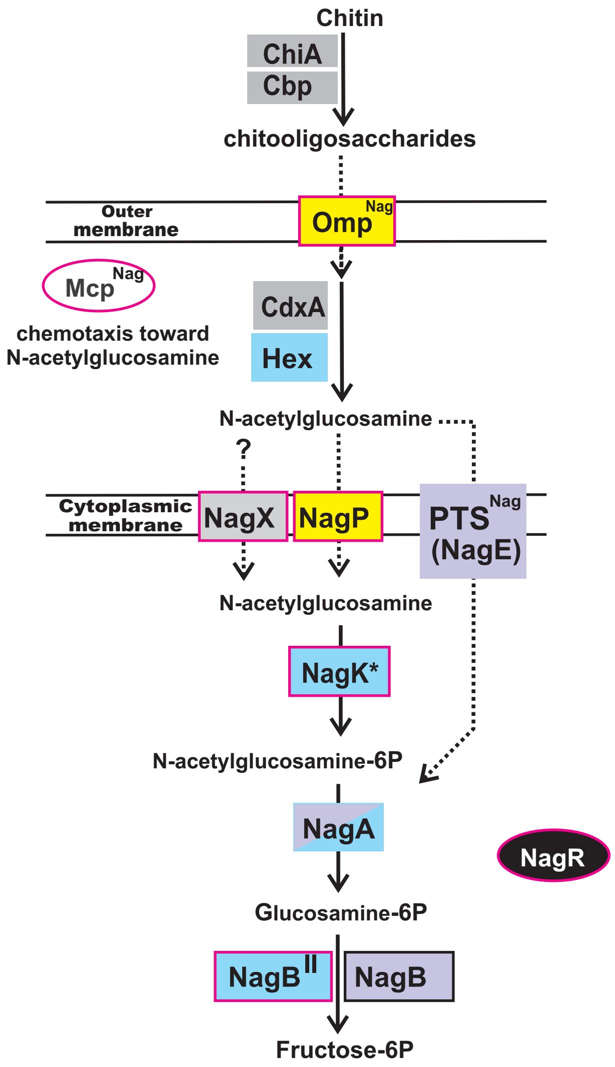

Known N-acetylglucosamine utilization pathway in *E.coli*

nagE	PTS system, N-acetylglucosamine-specific IIABC component
nagA	N-acetylglucosamine-6-phosphate deacetylase (EC 3.5.1.25)
nagB	Glucosamine-6-phosphate deaminase (EC 3.5.99.6)
nagC	N-acetylglucosamine-6P-responsive transcriptional repressor NagC, ROK family

Novel N-acetylglucosamine and chitin utilization pathway in *Shewanella* spp.

omp Nag	TonB-dependent outer membrane transporter for chitooligosaccharides [SO3514]
nagP	N-acetyl glucosamine transporter, NagP [SO3503]
nagK	N-acetylglucosamine kinase of eukaryotic type (EC 2.7.1.59) [SO3507]
nagA	N-acetylglucosamine-6-phosphate deacetylase (EC 3.5.1.25) [SO3505]
nagB II	Glucosamine-6-phosphate deaminase [isomerizing], alternative (EC 3.5.99.6) [SO3506]
hex	Beta-hexosaminidase (EC 3.2.1.52) [SO3509]
nagR	Transcriptional regulator of N-acetylglucosamine utilization, LacI family [SO3516]
nagX	N-acetylglucosamine related transporter, NagX [SO3504]
mcp Nag	N-acetylglucosamine-regulated methyl-accepting chemotaxis protein [SO3510]
cbp	Chitin binding protein [SO1072]
cdxA	Chitodextrinase precursor (EC 3.2.1.14) [Sbal_2491]
chiA	Chitinase (EC 3.2.1.14) [Sbal_3083]

novel functional roles/genes predicted in this study

● predicted and validated NagR binding sites

○ known NagC binding sites

B. D-glycerate (Grt) and D-glucarate/D-galactarate utilization pathways.

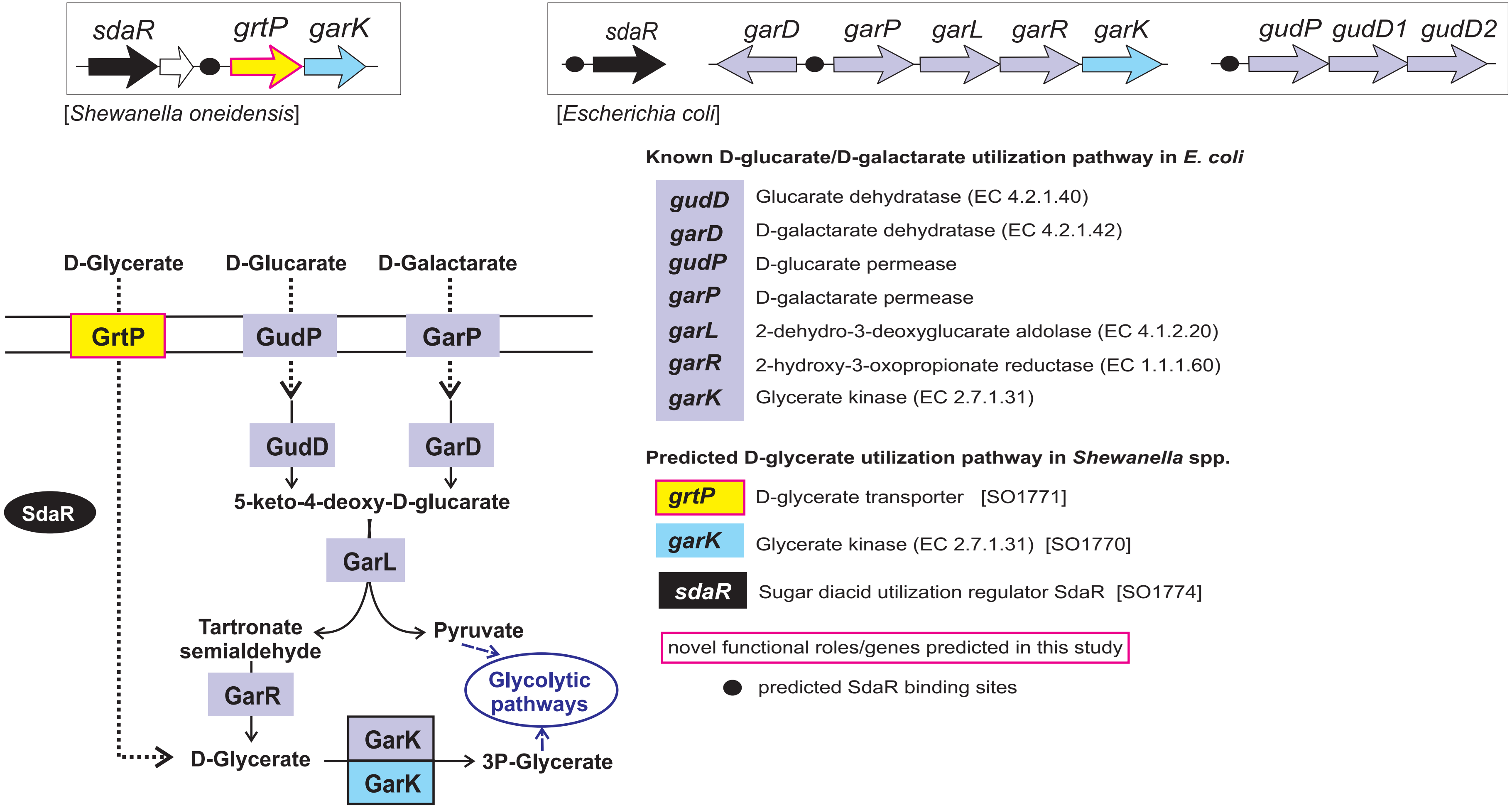

C. β-glucoside (Bgl) utilization pathway.

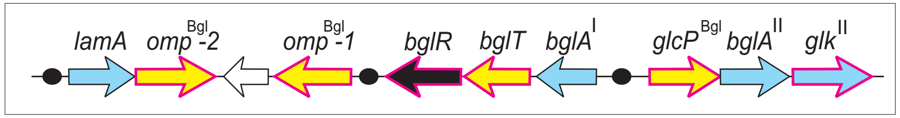

[*Shewanella amazonensis*]

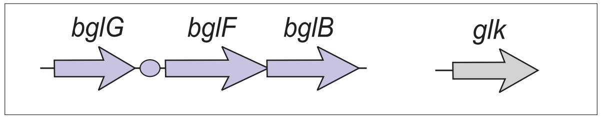

[*Escherichia coli*]

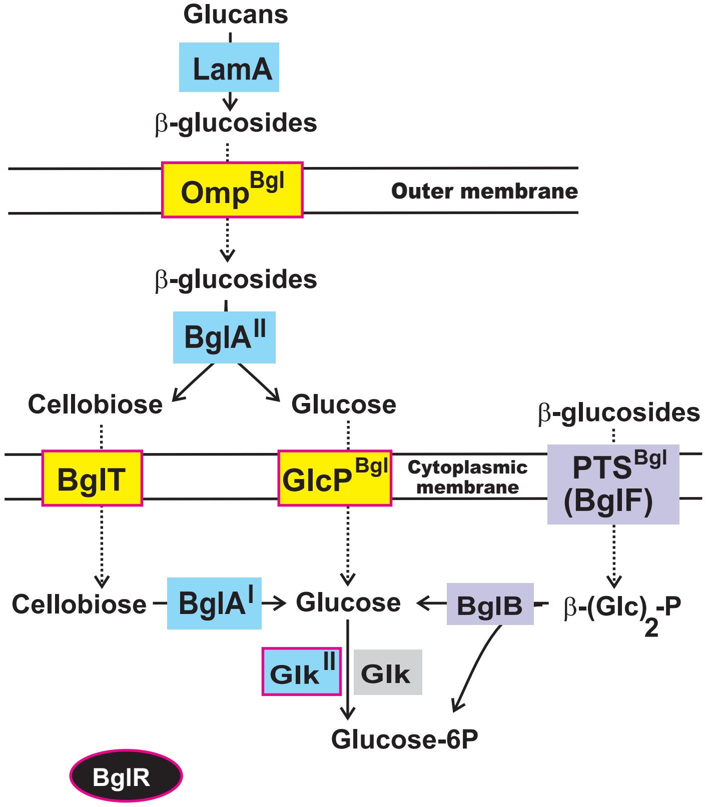

Known cellobiose utilization pathway in Enterobacteria (cryptic operon in *E.coli*)

bglG	Beta-glucoside *bgl* operon antiterminator, BglG family
bglF	PTS system, β-glucoside-specific IIABC component
bglB	6-phospho-β-glucosidase (EC 3.2.1.86)
glk	Glucokinase (EC 2.7.1.2)

Predicted β-glucoside/cellobiose utilization pathway in *Shewanella* spp.

omp ***Bgl***	TonB-dependent outer membrane transporter for β-glucosides [Sfri_1313]
bglT	β-glucoside transporter, GPH family [Sfri_1315]
glcP ***Bgl***	Glucose transporter in β-glucoside utilization gene cluster [Sfri_1317]
lamA	β-glucanase precursor (EC 3.2.1.73) [Sfri_1319]
bglA **II**	Periplasmic β-glucosidase (EC 3.2.1.21) [Sfri_1318]
bglA **I**	Cytoplasmic β-glucosidase (EC 3.2.1.21) [Sfri_1317]
bglR	Transcriptional regulator of β-glucosides utilization, LacI family [Sfri_1314]
glk **II**	Glucokinase, ROK family (EC 2.7.1.2) [Sfri_1321]

novel functional roles/genes predicted in this study

● predicted BglR binding sites ○ known BglG binding sites

D. Sucrose (Scr) utilization pathway.

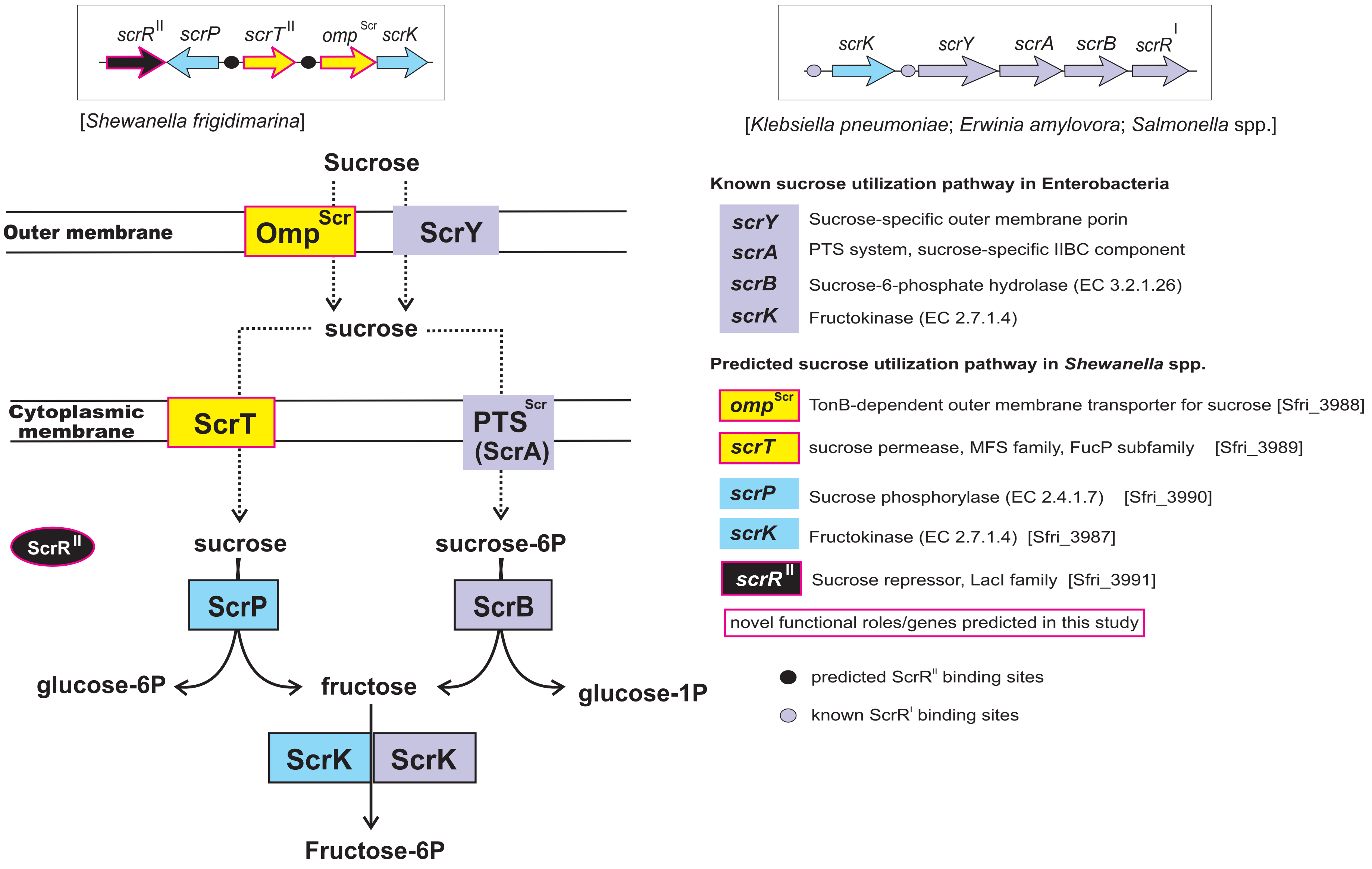

E. L-arabinose (Ara) and arabinosides utilization pathways.

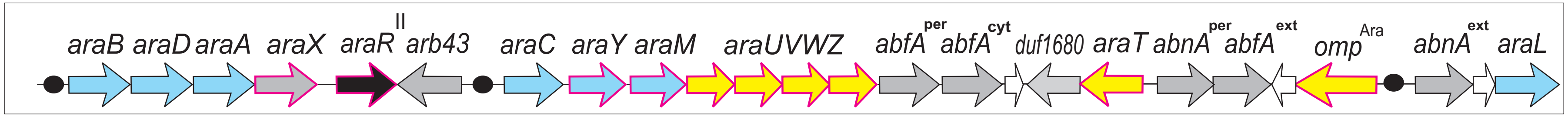

[*Shewanella* sp. MR-4]

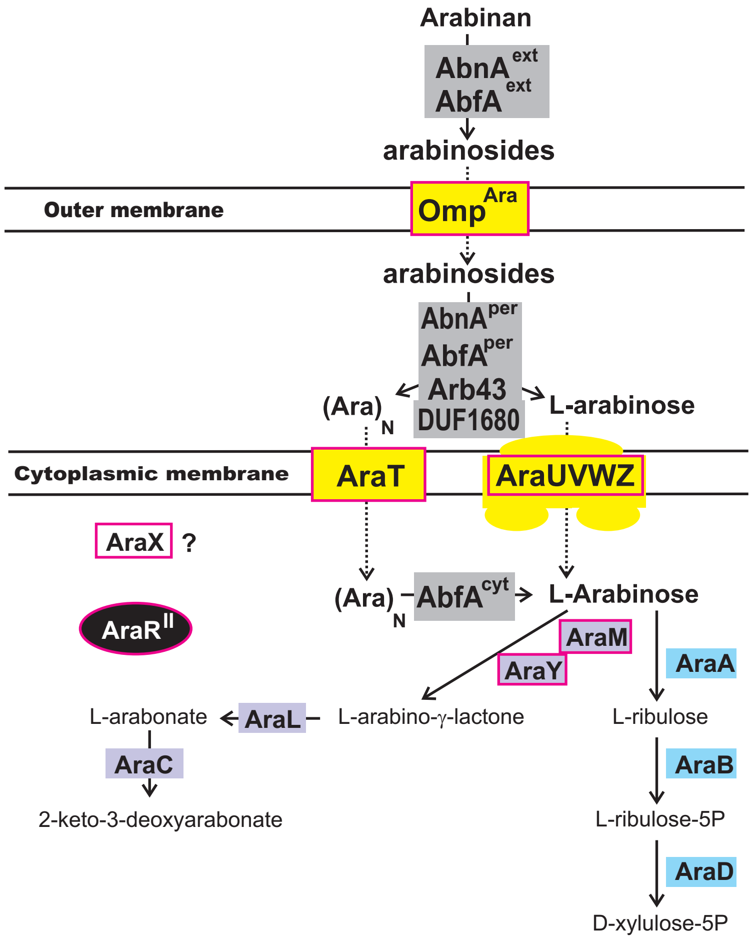

Extracellular secreted Ara hydrolases

abnA^{ext}	Arabinan endo-1,5-alpha-L-arabinosidase (EC 3.2.1.99) [Shewmr4_1999]
abfA^{ext}	Alpha-L-arabinofuranosidase II precursor (EC 3.2.1.55) [Shewmr4_1996]

Periplasmic Ara hydrolases

abnA^{per}	Arabinan endo-1,5-alpha-L-arabinosidase (EC 3.2.1.99) [Shewmr4_1995]
abfA^{per}	Alpha-L-arabinofuranosidase II precursor (EC 3.2.1.55) [Shewmr4_1990]
arb43	Hypothetical glycoside hydrolase, family 43, similar to arabinosidase [Shewmr4_1982]
duf1680	Hypothetical periplasmic glycoside hydrolase, family DUF1680 [Shewmr4_1993]

Cytoplasmic Ara hydrolase

abfA^{cyt}	Alpha-L-arabinofuranosidase II precursor (EC 3.2.1.55) [Shewmr4_1991]

Transporters

omp^{Ara}	TonB-dependent outer membrane transporter for arabinosides [Shewmr4_1998]
araT	Possible GPH family transporter (TC 2.A.2) for arabinosides [Shewmr4_1994]
araUVWZ	Predicted L-arabinose ABC transport system [Shewmr4_1986-87-88-89]

Conventional Ara catabolic pathway (the same as in *E. coli*, *B. subtilis*)

araB	Ribulokinase (EC 2.7.1.16) [Shewmr4_1977]
araD	L-ribulose-5-phosphate 4-epimerase (EC 5.1.3.4) [Shewmr4_1978]
araA	L-arabinose isomerase (EC 5.3.1.4) [Shewmr4_1979]

Alternative Ara catabolic pathway (AraL and AraC are the same as in *Azospirillum brasiliense*)

araY	predicted L-arabinose 1-dehydrogenase (EC 1.1.1.46) [Shewmr4_1984]
araC	L-arabonate dehydratase (EC 4.2.1.25) [Shewmr4_1983]
araL	L-arabinolactonase (EC 3.1.1.15) [Shewmr4_2001]
araM	L-arabinose-specific 1-epimerase (mutarotase) [Shewmr4_1985]

Hypothetical roles and transcriptional regulators:

araX	Hypothetical phosphoglucomutase family hydrolase in arabinose utilization cluster [Shewmr4_1980]
araR^{II}	Transcriptional regulator of the arabinose operon in Shewanella, GntR family [Shewmr4_1981]

● predicted AraR^{II} binding sites

novel functional roles/genes predicted in this study
