# Supplementary material for: Genomic encyclopedia of sugar utilization pathways in the Shewanella genus
Source: BMC Genomics. 2010 Sep 13;11:494. doi: 10.1186/1471-2164-11-494 (PMC2996990; doi:10.1186/1471-2164-11-494)

Additional file 10. The gluconate (A) and ribose (B) utilization gene loci in some closely related *Shewanella* strains and other γ-proteobacteria.

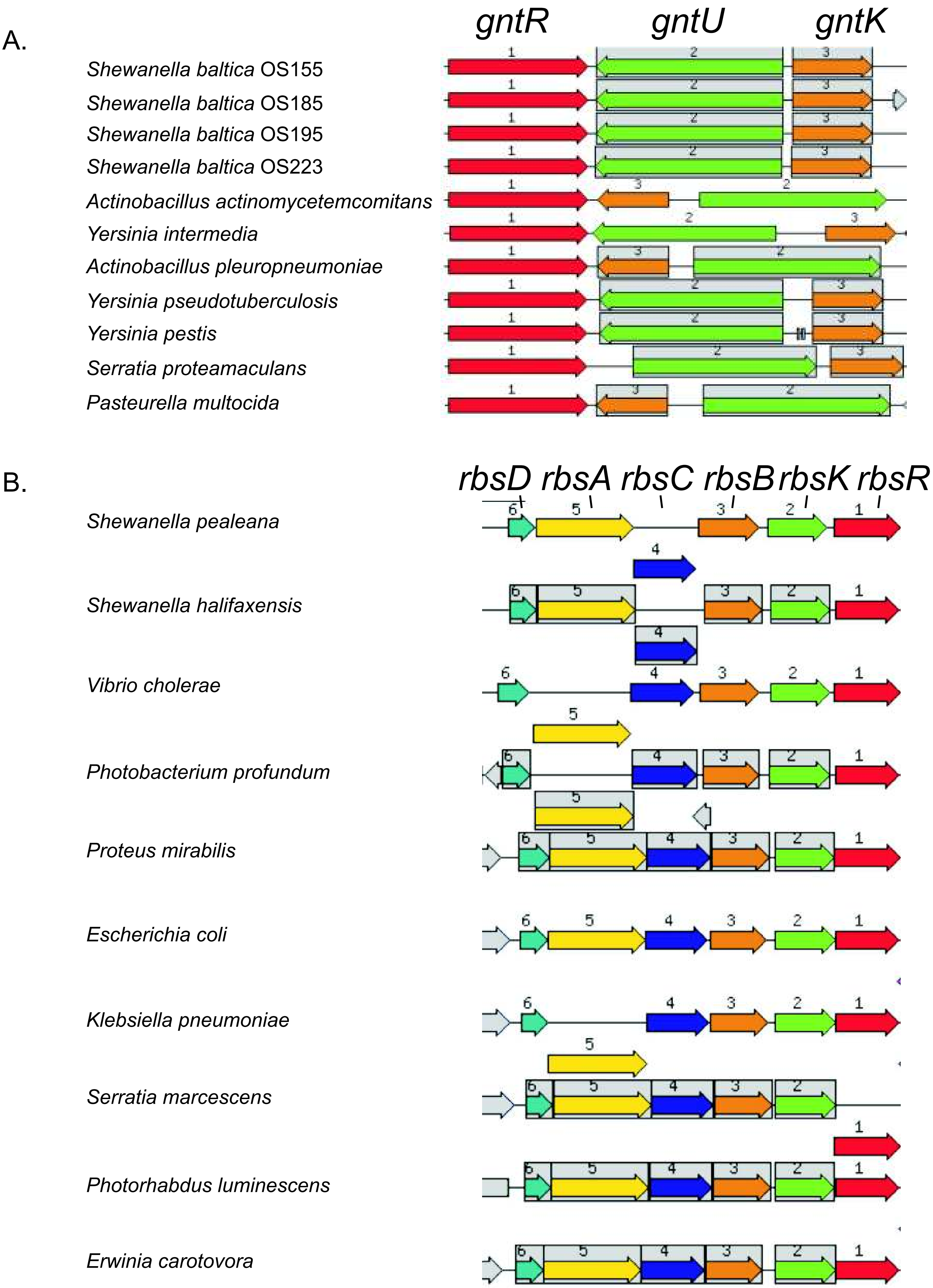

Supplement: Additional file 10 — The gluconate (A) and ribose (B) utilization gene loci in some closely related Shewanella strains and other γ-proteobacteria. [file 1471-2164-11-494-S10.PDF]
